# Periodontitis in patients with diabetes and its association with diabetes-related complications. A register-based cohort study

**DOI:** 10.1136/bmjopen-2024-087557

**Published:** 2024-07-04

**Authors:** Anna Trullenque-Eriksson, Cristiano Tomasi, Katarina Eeg-Olofsson, Tord Berglundh, Max Petzold, Jan Derks

**Affiliations:** 1 Department of Periodontology, Institute of Odontology, Sahlgrenska Academy, University of Gothenburg, Gothenburg, Sweden; 2 Swedish National Diabetes Register, Gothenburg, Sweden; 3 Institute of Medicine, Sahlgrenska Academy, University of Gothenburg, Gothenburg, Sweden

**Keywords:** ORAL MEDICINE, DIABETES & ENDOCRINOLOGY, GENERAL MEDICINE (see Internal Medicine), Diabetes & endocrinology

## Abstract

**Abstract:**

**Objective:**

To evaluate the association between type 1 diabetes (T1D)/type 2 diabetes (T2D) and periodontitis and assess the influence of periodontitis on diabetes-related complications.

**Design:**

Observational study; longitudinal analysis of register data.

**Setting:**

Swedish primary care centres, hospitals and dental clinics reporting to nationwide healthcare registers (2010–2020).

**Participants:**

28 801 individuals with T1D (13 022 women; mean age 42 years) and 57 839 individuals without diabetes (non-T1D; 26 271 women; mean age 43 years). 251 645 individuals with T2D (110 627 women; mean age 61 years) and 539 805 individuals without diabetes (non-T2D; 235 533 women; mean age 60 years). Diabetes and non-diabetes groups were matched for age, gender and county of residence.

**Main outcome measures:**

Prevalent periodontitis, diabetes-related complications (retinopathy, albuminuria, stroke and ischaemic heart disease) and mortality.

**Results:**

Periodontitis was more common among T2D (22%) than non-T2D (17%). Differences were larger in younger age groups (adjusted RR at age 30–39 years 1.92; 95% CI 1.81 to 2.03) and exacerbated by poor glycaemic control. Periodontitis prevalence was 13% in T1D and 11% in non-T1D; only the subgroup with poor glycaemic control was at higher risk for periodontitis. Periodontitis was associated with a higher incidence of retinopathy (T1D: HR 1.08, 95% CI 1.02 to 1.14; T2D: HR 1.08, 95% CI 1.06 to 1.10) and albuminuria (T1D: HR 1.14, 95% CI 1.06 to 1.23; T2D: HR 1.09, 95% CI 1.07 to 1.11). Periodontitis was not associated with a higher risk for stroke, cardiovascular disease or higher mortality in T1D/T2D.

**Conclusions:**

The association between T2D and periodontitis was strong and exacerbated by poor glycaemic control. For T1D, the association to periodontitis was limited to subgroups with poor glycaemic control. Periodontitis contributed to an increased risk for retinopathy and albuminuria in T1D and T2D.

**STRENGTHS AND LIMITATIONS OF THIS STUDY**

- The population-wide approach, including over 800 000 individuals, allowed for solid estimates from real-world data.
- The longitudinal register data covered a 10-year period.
- By matching several registers, we were able to adjust for socioeconomic parameters.
- The periodontitis case definition was based exclusively on clinical recordings of periodontal probing depth.
- Information on tobacco smoking and body mass index were not available for the control group.

## Introduction

 Periodontitis is a highly prevalent oral disorder characterised by soft tissue inflammation, loss of periodontal attachment and, ultimately, tooth loss. The typical age of onset lies between 20 and 30 years, and, in its severe forms, periodontitis affects roughly 10% of adults worldwide.^1^,^3^ Observational data suggest that periodontitis is associated with type 2 diabetes (T2D) and contributes to diabetes-related complications.^4 5^ This association was highlighted in a recent statement by WHO.^6^ However, corresponding data on the association between type 1 diabetes (T1D) and periodontitis are highly limited.^4 7 8^

Register-based research offers the possibility for longitudinal evaluations of population data. There are numerous, well-established healthcare registers in Sweden and their completeness in terms of target populations is generally high.^9^ The Swedish National Diabetes Register (NDR) includes 85% of all Swedish adults diagnosed with T2D, while the completeness for T1D is >90%.^10^ From 2010 and onwards, the Swedish Quality Registry for Caries and Periodontal Disease (SKaPa) administers data with a high level of completeness based on the daily, automated retrieval of information from electronic patient dental records. To exemplify, during the period 2020–2022, findings from routine dental examinations were recorded for 3.3 million adults.^11^ Access to additional population registries, providing medical and socioeconomic data, allows for robust and detailed descriptions of populations with or without diabetes.

The aim of the present register-based study was to evaluate the association between diabetes (T1D and T2D) and periodontitis on a population level. A further aim was to assess the influence of periodontitis on diabetes-related complications.

## Methods

### Study design and participants

This retrospective study was based on longitudinal data obtained from multiple Swedish national registers. Using the Swedish NDR, we identified one cohort of individuals with T1D (diagnosis by 2020 and ≥1 prescription of insulin, National Prescribed Drug Registry) and a second cohort with T2D (diagnosis by 2020). Individuals aged ≥18 years in 2010 were considered (online supplemental appendix p. 5). For each cohort, a control sample was randomly selected from a non-diabetes population identified in the Swedish Total Population Register (control/case ratio 2:1; matched for age, gender and county of residence). From these four cohorts, individuals without at least one entry in the national dental registry SKaPa (ie, no dental examination during the period 2010–2020) were excluded (online supplemental tables A1-A6).

### Patient and public involvement

There was no direct patient involvement in this study. No funds or time were allocated to patient and public involvement.

### Data sources and outcome measures

The occurrence of periodontitis and tooth loss (registered tooth extractions, regardless of the reason for extraction) was assessed annually over the 10-year study period in the SKaPa register. A periodontitis case was defined by the presence of ≥3 teeth with probing depths of ≥6 mm, assessed by a dental professional during a routine clinical examination any time between 2010 and 2020 (online supplemental appendix p 6). Third molars were not considered for either outcome.

We used blood haemoglobin A1c (HbA1c) levels to describe glycaemic control (NDR), which was categorised as good (HbA1c <52 mmol/mol for ≥75% of their observation period) or poor (HbA1c >62 mmol/mol for≥75% of their observation period) based on longitudinal readings (at least 5 years). We also explored median glycaemic control as a continuous parameter (online supplemental appendix p 7).

Data on diabetes-related complications were obtained from the NDR (retinopathy and albuminuria) and the National Patient Register (stroke and ischaemic heart disease). Information on mortality was extracted from the Cause of Death Register and socioeconomic parameters were retrieved from the Longitudinal Integrated Database for Health Insurance and Labour Market Studies (online supplemental appendix pp 5–7). Datasets from the different registers were merged using unique national personal identity numbers as identifiers.

The study was approved by the Swedish Ethical Review Authority (Dnr: 2019–04140).

### Statistical analysis

Probability estimates for prevalent periodontitis among individuals with T1D, T2D and their respective controls were obtained through multiple logistic regression models. Adjusted risk ratios (RR) were recalculated from ORs based on *margins*.^12^ The annual incidence rates for tooth loss and their ratios (IRR) were estimated with Poisson regression models. The extent of tooth loss (no tooth extractions, 1–4 extractions or ≥5 extractions) over the 10-year period was analysed using multinomial logistic regression models (RR). Analyses were repeated, comparing subgroups of T1D and T2D by glycaemic control, using their matched control groups as references.

Within the T1D and T2D cohorts, the effect of periodontitis (at any time during the observation period) on diabetes-related complications and on mortality was described by HR obtained through Cox regression analyses. To account for age and cohort effects, we also used Poisson-based age-period-cohort (APC) modelling^13^ (*poprisktime*^14^ and *apcspline*^15^ functions) to estimate IRRs.

Age categorisation was based on age in 2010 (the start of the observation period). Gender (referring to legal sex, as recorded in the registers) was accounted for, either as a covariate or by stratification. We adjusted for level of education and income due to observed socioeconomic imbalances (table 1). Hence, all models were adjusted for age (and its interaction with diabetes), gender, level of education and income.

**Table 1:** Individuals with type 1 and type 2 diabetes and their respective matched controls without diabetes

	Group			
	No diabetes		Type 1 diabetes	
Gender				
Female	26 271	45.4%	13 022	45.2%
Male	31 568	54.6%	15 779	54.8%
Age in 2010	42.9	(16.9)	42.4	(16.5)
Year of birth				
1981–1992	15 984	27.6%	8071	28.0%
1971–1980	10 066	17.4%	5134	17.8%
1961–1970	11 227	19.4%	5757	20.0%
1951–1960	9 253	16.0%	4635	16.1%
1941–1950	7 545	13.0%	3571	12.4%
≤1940	3 764	6.5%	1633	5.7%
Education (latest available)				
Up to lower secondary education	7818	13.6%	4083	14.2%
Upper secondary to post-secondary education <2 years	29 831	51.8%	15 214	53.1%
Post-secondary ≥2 years to tertiary education	19 965	34.7%	9362	32.7%
Annual income (SEK; latest available)	300 900	(275 600)	266 700	(271 600)
Years in lowest fifth percentile of income (2005–2019)	0.7	(1.9)	0.8	(2)
0	45 123	78.0%	22 128	76.8%
1–4 years	9923	17.2%	5081	17.6%
≥5 years	2793	4.8%	1589	5.5%
Systemic conditions (2005–2020)*				
Certain infectious and parasitic diseases (A00–B99)	1702	2.9%	10 768	37.4%
Neoplasms (C00–D48)	15 418	26.7%	10 423	36.2%
Cancer (C00–C97)	6079	10.5%	2684	9.3%
Endocrine, nutritional and metabolic diseases (E00–E90)	7910	13.7%	28 664	99.5%
Obesity (E66)	1761	3%	2205	7.7%
Diseases of the circulatory system (I00–I99)	14 314	24.7%	14 465	50.2%
Ischaemic heart diseases (I20–I25)	2648	4.6%	3000	10.4%
Stroke (I60, I61, I63, I64 and G45)	1298	2.2%	1186	4.1%
Diseases of the genitourinary system (N00–N99)	1	0.0%	9	0.0%
Nephritis, nephrotic syndrome and nephrosis (N00–N07, N17–N19, N25–N27)	1	0.0%	8	0.0%

	Group			
	No diabetes		Type 2 diabetes	
Gender				
Female	235 533	43.6%	110 627	44.0%
Male	304 272	56.4%	141 018	56%
Age in 2010	60.1	(13.3)	60.7	(13.1)
Year of birth				
1981–1992	10 348	1.9%	4 217	1.7%
1971–1980	28 120	5.2%	11 576	4.6%
1961–1970	75 431	14.0%	33 536	13.3%
1951–1960	124 714	23.1%	58 104	23.1%
1941–1950	168 295	31.2%	80 540	32.0%
≤1940	132 897	24.6%	63 672	25.3%
Education (latest available)				
Up to lower secondary education	134 919	25.1%	80 780	32.4%
Upper secondary to post-secondary education <2 years	262 874	48.9%	124 894	50.2%
Post-secondary ≥2 years to tertiary education	139 526	26.0%	43 312	17.4%
Annual income (SEK; latest available)	197 400	(202 400)	168 500	(133 200)
Years in lowest fifth percentile of income (2005–2019)	0.5	(1.9)	0.8	(2.3)
0	464 008	86%	203 105	80.7%
1–4 years	53 916	10%	32 265	12.8%
≥5 years	21 880	4.1%	16 267	6.5%
Systemic conditions (2005–2020)*				
Certain infectious and parasitic diseases (A00–B99)	37 332	6.9%	35 971	14.3%
Neoplasms (C00–D48)	208 763	38.7%	104 478	41.5%
Cancer (C00–C97)	121 118	22.4%	57 907	23.0%
Endocrine, nutritional and metabolic diseases (E00–E90)	110 280	20.4%	186 300	74.0%
Obesity (E66)	11 641	2.2%	30 423	12.1%
Diseases of the circulatory system (I00–I99)	253 533	47%	170 748	67.9%
Ischaemic heart diseases (I20–I25)	64 083	11.9%	56 417	22.4%
Stroke (I60, I61, I63, I64 and G45)	31 514	5.8%	23 098	9.2%
Diseases of the genitourinary system (N00–N99)	18	0%	34	0%
Nephritis, nephrotic syndrome and nephrosis (N00–N07, N17–N19 and N25–N27)	12	0%	24	0%

Categorical data are presented as frequencies and percentages. The continuous variables age and number of years in the lowest fifth percentile of income are presented as mean (SD); income is presented as median (IQR).

* Systemic conditions exclude diagnoses only registered in primary care. International Classification of Diseases 10th revision codes provided in parentheses.

All estimates were accompanied by 95% CIs. Full regression models are provided in online supplemental appendix. Multiple sensitivity analyses were carried out (online supplemental tables A7-A8; online supplemental appendix pp. 14–27) and we explored alternative case definitions for periodontitis. All analyses were performed in Stata/SE V.17.0.

## Results

**Figure 1:**
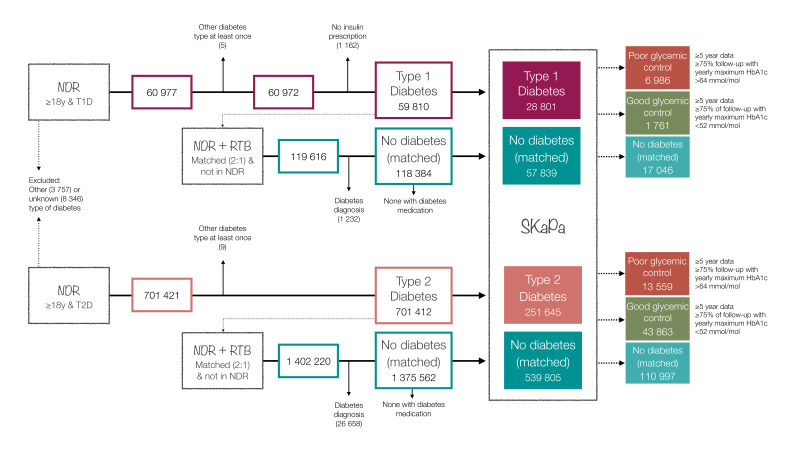
Sample description (number of individuals in the different cohorts and subgroups). For the period 2010–2020, mean follow-up time within SKaPa was 6.1±3.6 years for T1D, 6.1±3.7 years non-T1D, 5.5±3.7 years for T2D and 5.7±3.7 years for non-T2D; mean follow-up time within NDR was 8.4±2.5 years for T1D and 5.7±3.5 years for T2D. See also online supplemental appendix (pp 5, 8–13). NDR, National Diabetes Register; RTB, Swedish Total Population Register; SKaPa, Swedish Quality Registry for Caries and Periodontal Disease; T1D, type 1 diabetes; T2D, type 2 diabetes.

The number of individuals in each cohort in the final dataset was 28 801 (T1D), 57 839 (non-T1D controls), 251 645 (T2D) and 539 805 (non-T2D controls), respectively (figure 1; table 1; online supplemental appendix pp 8–13).

### Diabetes and periodontitis

**Figure 2:**
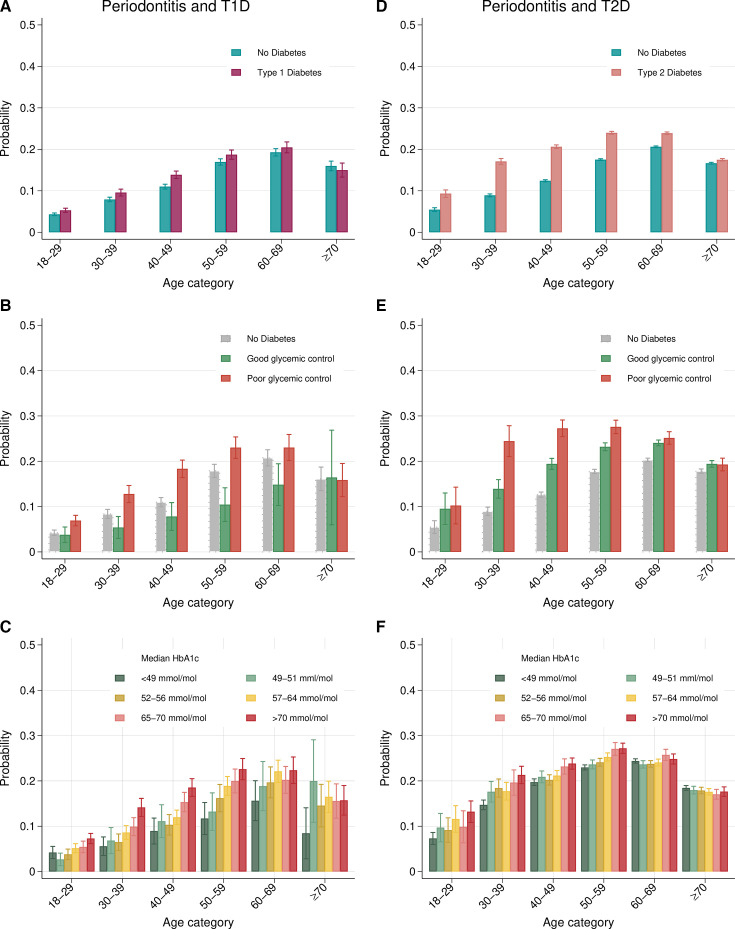
Probability estimates for periodontitis by age category. Periodontitis and T1D models are based on (A) 85 042 individuals (T1D 28 241 and non-T1D 56 801), (B) 25 302 individuals (good glycaemic control 1740, poor glycaemic control 6812, no diabetes 16 750) and (C) 28 130 individuals with T1D. Periodontitis and T2D models are based on (D) 770 672 individuals (T2D 243 900 and non-T2D 526 772), (E) 163 981 individuals (good glycaemic control 42 660, poor glycaemic control 13 038 and no diabetes 108 283) and (F) 240 307 individuals with T2D. Models were adjusted for age (categorical), gender, level of education and income.

Periodontitis was more common in T1D and T2D than in their respective control groups (T1D: 12.6%, 95% CI 12.2 to 13.0; non-T1D: 11.1%, 95% CI 10.9 to 11.4; T2D: 21.6%, 95% CI 21.5 to 21.8; non-T2D: 16.8%, 95% CI 16.7 to 16.9), with RRs of 1.13 (95% CI 1.09, 1.18) and 1.26 (95% CI 1.24, 1.27), respectively. The association with periodontitis was stronger for T2D, as evidenced by risk differences of up to 8% (figure 2 and online supplemental figures A1-A4; online supplemental tables A9-A10; online supplemental appendix pp 28–33).

Glycaemic control had a decisive impact on associations with periodontitis. Poor glycaemic control was associated with an increased risk for periodontitis by up to 67% in T1D (overall RR 1.37, 95% CI 1.28 to 1.47; RR at 40–49 years 1.67, 95% CI 1.45 to 1.92) and up to 172% in T2D (overall RR 1.38, 95% CI 1.33 to 1.43; RR at age 30–39 years 2.72, 95% CI 2.29 to 3.23) compared with non-T1D and non-T2D, respectively (figure 2 and online supplemental figures A5-A8; online supplemental tables A11-A12; online supplemental appendix pp 34–39). Good glycaemic control was associated with a lower risk for periodontitis in T1D (RR 0.71, 95% CI 0.60 to 0.84), but not in T2D (RR 1.23, 95% CI 1.20 to 1.26).

Prevalence and RRs by age category and gender are presented in online supplemental tables A9, A10. Differences in terms of the prevalence of periodontitis between the diabetes cohorts and matched controls were most prominent in younger age categories. Overall, periodontitis was more frequent among males than females. The relative effect of diabetes in young females was stronger than in young males, as illustrated by greater RRs for both T1D and T2D.

Tooth loss was more common in T1D and T2D than in their respective control groups (T1D: 33.9%, 95% CI 33.4 to 34.5; non-T1D: 29.0%, 95% CI 28.6 to 29.4; T2D: 46.2%, 95% CI 46.0 to 46.4; non-T2D: 37.8%, 95% CI 37.7 to 38). Incident tooth loss was greater in T1D (IRR 1.28; 95% CI 1.26, 1.30) and T2D (IRR 1.37; 95% CI 1.36 to 1.38) than in control groups (figure 3, online supplemental figures A9-A12; online supplemental appendix pp 40–45). Poor glycaemic control was associated with a higher risk for tooth loss in T1D and T2D compared with non-T1D/non-T2D. Well-controlled individuals were at higher risk only in T2D (figure 3 and online supplemental figures A1-A18; online supplemental appendix pp 46–51).

**Figure 3:**
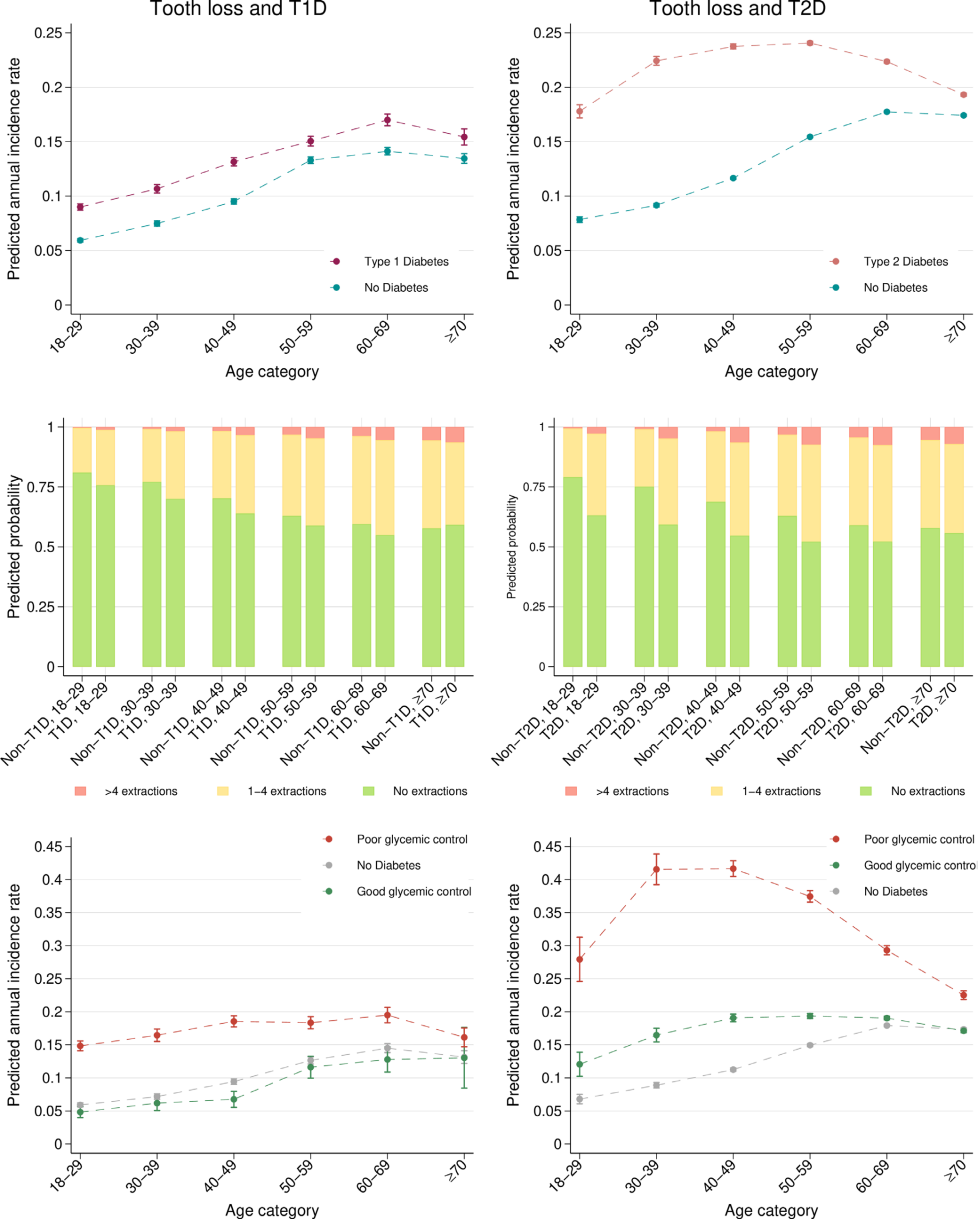
Estimated annual incidence rates of tooth loss and probability estimates for the extent of tooth loss during the 10-year observation period by age category. The tooth loss and T1D models are based on 86 273 individuals (T1D 28 659 and non-T1D 57 614) and 25 683 individuals (good glycaemic control 1756, poor glycaemic control 6947 and no diabetes 16 980). The tooth loss and T2D models are based on 786 305 individuals (T2D 248 986 and non-T2D 537 319) and 167 362 individuals (good glycaemic control 43 544, poor glycaemic control 13 321 and no diabetes 110 497). Models were adjusted for age (categorical), gender, level of education and income.

### Diabetes-related complications

In both T1D and T2D, periodontitis was associated with a higher risk for retinopathy (T1D HR 1.08, 95% CI 1.02 to 1.14; T2D HR 1.08, 95% CI 1.06 to 1.10) and albuminuria (T1D HR 1.14, 95% CI 1.06 to 1.23; T2D HR 1.09, 95% CI 1.07 to 1.11), as confirmed by both adjusted Cox regression analyses and APC modelling (tables2  3 and online supplemental tables A13-A14; online supplemental figures A19-A38; online supplemental appendix pp 52–63). Periodontitis was not associated with a higher risk for ischaemic heart disease, stroke or death in either T1D or T2D.

**Table 2:** Diabetes-related complications in individuals with type 1 diabetes (comparing periodontitis to no periodontitis).

		Age						
		**18–29**	**30–39**	**40–49**	**50–59**	**60–69**	**≥70**	**Overall**
Retinopathy(2010–2020)	No periodontitis	5342/7418 (72.0%)	3611/4528 (79.7%)	3935/4778(82.4%)	2976/3613 (82.4%)	2273/2668(85.2%)	981/1204(81.5%)	19 118/24 209(79.0%)
	Periodontitis	313/404(77.5%)	366/456(80.3%)	644/781(82.5%)	766/868(88.2%)	625/742(84.2%)	209/255(82.0%)	2923/3506(83.4%)
	Adjusted HR (95% CI)***:*** 1.08 (1.02 to 1.14)							
Albuminuria(2010–2020)	No periodontitis	1135/7426(15.3%)	962/4531(21.2%)	1353/4782(28.3%)	1280/3617(35.4%)	1202/2697(44.6%)	660/1238(53.3%)	6592/24 291(27.1%)
	Periodontitis	91/403(22.6%)	132/459(28.8%)	276/783(35.2%)	340/870(39.1%)	314/748(42.0%)	135/258(52.3%)	1288/3521(36.6%)
	Adjusted HR (95% CI): 1.14 (1.06 to 1.23)							
Ischaemic heart disease(2010–2020)	No periodontitis	22/7594(0.3%)	82/4595(1.8%)	345/4855(7.1%)	630/3673(17.2%)	694/2748(25.3%)	490/1342(36.5%)	2263/24 807(9.1%)
	Periodontitis	2/415(0.5%)	13/465(2.8%)	69/790(8.7%)	141/880(16.0%)	168/757(22.2%)	97/268(36.2%)	490/3575(13.7%)
	Adjusted HR (95% CI): 0.96 (0.86 to 1.08)							
Stroke(2010–2020)	No periodontitis	21/7594(0.3%)	57/4595(1.2%)	138/4855(2.8%)	184/3673(5.0%)	205/2748(7.5%)	197/1342(14.7%)	802/24 807(3.2%)
	Periodontitis	3/415(0.7%)	8/465(1.7%)	28/790(3.5%)	43/880(4.9%)	59/757(7.8%)	40/268(14.9%)	181/3575(5.1%)
	Adjusted HR (95% CI): 1.05 (0.89 to 1.25)							
Mortality(2010–2020)	No periodontitis	67/7594(0.9%)	63/4595(1.4%)	163/4855(3.4%)	251/3673(6.8%)	466/2748(17.0%)	638/1342(47.5%)	1648/24 807(6.6%)
	Periodontitis	3/415(0.7%)	6/465(1.3%)	24/790(3.0%)	75/880(8.5%)	117/757(15.5%)	118/268(44.0%)	343/3575(9.6%)
	Adjusted HR (95% CI): 0.91 (0.81 to 1.02)							

**Table 3:** Diabetes-related complications in individuals with type 2 diabetes (comparing periodontitis to no periodontitis).

		Age						
		**18–29**	**30–39**	**40–49**	**50–59**	**60–69**	**≥70**	**Overall**
Retinopathy(2010–2020)	No periodontitis	688/2748 (25.0%)	2260/7520 (30.1%)	7315/22 012 (33.2%)	13 520/37 659 (35.9%)	21 636/53 201 (40.7%)	18 037/41 983 (43.0%)	63 456/165 123(38.4%)
	Periodontitis	89/299 (29.8%)	551/1561 (35.3%)	2272/5950 (38.2%)	4927/12 221 (40.3%)	7391/17 205 (43.0%)	4257/9438 (45.1%)	19 487/46 674(41.8%)
	Adjusted HR (95% CI): 1.08 (1.06 to 1.10)							
Albuminuria(2010–2020)	No periodontitis	823/3096 (26.6%)	2207/8200 (26.9%)	6895/23 378 (29.5%)	12 793/39 270 (32.6%)	22 009/55 027 (40.0%)	22 212/44 491 (49.9%)	66 939/173 462 (38.6%)
	Periodontitis	89/348 (25.6%)	545/1718 (31.7%)	2138/6296 (34.0%)	4852/12 826 (37.8%)	7859/17 860 (44.0%)	5214/9973 (52.3%)	20 697/49 021 (42.2%)
	Adjusted HR (95% CI): 1.09 (1.07 to 1.11)							
Ischaemic heart disease(2010–2020)	No periodontitis	26/3757 (0.7%)	284/9363 (3.0%)	2005/25 967 (7.7%)	6323/42 932 (14.7%)	13 444/59 634 (22.5%)	17 581/51 519 (34.1%)	39 663/193 172 (20.5%)
	Periodontitis	6/390(1.5%)	91/1930 (4.7%)	675/6884 (9.8%)	2351/13 851 (17.0%)	4504/19 182 (23.5%)	3554/11 080 (32.1%)	11 181/53 317 (21.0%)
	Adjusted HR (95% CI): 0.96 (0.94 to 0.99)							
Stroke(2010–2020)	No periodontitis	23/3757 (0.6%)	131/9363 (1.4%)	678/25 967 (2.6%)	1852/42 932 (4.3%)	4623/59 634 (7.8%)	7480/51 519 (14.5%)	14 787/193 172 (7.7%)
	Periodontitis	2/390(0.5%)	23/1930 (1.2%)	182/6884 (2.6%)	669/13 851 (4.8%)	1597/19 182 (8.3%)	1625/11 080 (14.7%)	4098/53 317 (7.7%)
	Adjusted HR (95% CI): 0.99 (0.95 to 1.03)							
Mortality(2010–2020)	No periodontitis	29/3757 (0.8%)	115/9363 (1.2%)	516/25 967 (2.0%)	1967/42 932 (4.6%)	6283/59 634 (10.5%)	20 617/51 519 (40.0%)	29 527/193 172 (15.3%)
	Periodontitis	6/390(1.5%)	19/1930 (1.0%)	134/6884 (1.9%)	619/13 851 (4.5%)	1989/19 182 (10.4%)	3606/11 080 (32.5%)	6373/53 317 (12.0%)
	Adjusted HR (95% CI): 0.81 (0.79 to 0.83)							

## Discussion

In this large population-based register study, we demonstrated an association between T2D and periodontitis. The association was strongest in younger age categories and exacerbated by poor glycaemic control. For T1D, only the subgroup with poor glycaemic control was at higher risk for periodontitis. Our results also indicated that periodontitis contributes to some diabetes-related complications, namely retinopathy and nephropathy (albuminuria). Periodontitis was not, however, associated with a higher risk for ischaemic heart disease, stroke or death in T1D/T2D.

Our findings on the association between T2D and periodontitis are in line with previous evidence and position papers presented by dental and medical professional associations.^16^ Evidence on the relationship between T1D and periodontitis is limited, as illustrated by study samples including no more than a few hundred patients.^57 17^,^19^ In a study on 1114 cases and 7253 controls without diabetes, Sun *et al*^20^ reported an adjusted HR of 1.7 for periodontitis in young individuals with T1D (20–40 years).^20^ Our study not only included a considerably larger number of individuals but also covered a larger age span. In this sense, the new evidence from the present study suggests that the association between T1D and periodontitis was largely dependent on glycaemic control rather than the diagnosis per se. In addition, good glycaemic control in this group may reflect healthcare attitudes in general, extending to better compliance levels also in terms of oral care. Regardless of potential mechanisms, the present data confirm the relevance of glycaemic control for oral and periodontal health.^21 22^

The association between periodontitis and diabetes (T1D and T2D) in the present study was most prominent up to the age of 50 years, particularly among females. Interestingly, other studies demonstrated that associations between periodontitis and cardiovascular diseases were also stronger in younger groups^23^ and females.^24^ We speculate that the interplay between the two conditions at a later stage in life may be masked by the accumulation of additional risk factors. The observed gender effect is not understood.

Previous evidence has linked periodontitis to an increase in incidence of micro- and macrovascular complications in patients with T2D (predominantly cross-sectional data)^4^ and T1D (one cross-sectional and one case-control study).^25 26^ In contrast, we noted an increased risk only for microvascular complications (retinopathy and albuminuria). Differences may be explained by characteristics of the study populations (eg, ethnicity), but potentially also by differences in access to healthcare.

The strengths of this study reside in the population-wide approach, facilitated by patient registers with high levels of coverage and the solid exposures and endpoints (diagnosis T1D/T2D, diabetes-related complications and mortality) captured by registered healthcare professionals. Also, matching to population registers through the unique national personal identity numbers allowed for adjustment for socioeconomic parameters. Some limitations need to be considered. The categorisation of a periodontitis case was extrapolated from the data available in SKaPa, that is, clinical recordings of periodontal probing depth, rather than a diagnostic code or detailed assessments of attachment levels. The direction of the associations was consistent when using alternative case definitions for periodontitis.

We had no information for the control group on tobacco smoking and body mass index, two well-established risk factors in the current context. However, smoking in T1D (13%) and T2D (14%) was not notably different from 2010 population estimates provided by the Swedish national public health survey (13% of 9933 respondents age 16–84 years were daily smokers).^27^ And finally, while we do have data on ‘first’ recordings of diabetes and periodontitis diagnoses, these may not necessarily correspond to actual onset of disease. Therefore, we avoided making assumptions on directionality and rather focused on comorbidity over the study period.

### Implications for clinicians and policymakers

Our focus on periodontitis as a primary target of our analysis is motivated by its negative impact on quality of life^28^ and the resulting economic burden on society and the individual.^29^ Economic aspects are particularly relevant in light of the already disadvantaged socioeconomic status of the T2D group in our study. Cost may act as a barrier to adequate periodontal care, which may improve glycaemic control.^30^ From a public health point of view, preventive strategies in risk groups should therefore consider including dental care.

## Conclusion

The present data demonstrate a strong association between T2D and periodontitis, exacerbated by poor glycaemic control. For T1D, the association to periodontitis was limited to subgroups with poor glycaemic control. Periodontitis contributed to an increased risk for retinopathy and albuminuria in both T1D and T2D.

## supplementary material

10.1136/bmjopen-2024-087557 online supplemental file 1

10.1136/bmjopen-2024-087557 online supplemental file 2

## Data Availability

Data are available upon reasonable request.
