# Supplementary material for: Periodontitis in patients with diabetes and its association with diabetes-related complications. A register-based cohort study
Source: BMJ Open. 2024 Jul 4;14(7):e087557. doi: 10.1136/bmjopen-2024-087557 (PMC11227830; doi:10.1136/bmjopen-2024-087557)
Supplement: online supplemental file 1 [file bmjopen-14-7-s001.docx]

Periodontitis in patients with diabetes and its association with diabetes-related complications. A register-based cohort study.

Appendix

Table of Contents

[Data retrieval from national registries 5](#_Toc167130826)

[Additional variable description 6](#_Toc167130827)

[Table A1. Included individuals with T1D and matched controls without diabetes. 8](#_Toc167130828)

[Table A2. Subgroups of included individuals with T1D according to glycemic control and their matched controls without diabetes. 9](#_Toc167130829)

[Table A3. Excluded individuals with T1D and matched controls without diabetes, lacking entry in SKaPa (period: 2010-2020). 10](#_Toc167130830)

[Table A4. Included individuals with T2D and matched controls without diabetes. 11](#_Toc167130831)

[Table A5. Subgroups of included individuals with T2D according to glycemic control and their matched controls without diabetes. 12](#_Toc167130832)

[Table A6. Excluded individuals with T2D and matched controls without diabetes, lacking entry in SKaPa (period: 2010-2020) 13](#_Toc167130833)

[Sensitivity analyses: Periodontitis 14](#_Toc167130834)

[Table A7. Summary of sensitivity analyses (outcome: periodontitis) 14](#_Toc167130835)

[Alternative case definition for periodontitis: ≥1 tooth with PPD ≥6 mm 14](#_Toc167130836)

[Logistic regression model T1D versus matched controls without diabetes (outcome: periodontitis) 14](#_Toc167130837)

[Logistic regression model T2D versus matched controls without diabetes (outcome: periodontitis) 14](#_Toc167130838)

[Number of teeth with PPD ≥6 mm 15](#_Toc167130839)

[Regression model T1D versus matched controls without diabetes (outcome: periodontitis extent) 15](#_Toc167130840)

[Regression model T2D versus matched controls without diabetes (outcome: periodontitis extent) 15](#_Toc167130841)

[Sensitivity analyses: Complications 16](#_Toc167130842)

[Table A8. Summary of sensitivity analyses (outcome: diabetes-related complications) 16](#_Toc167130843)

[Alternative case definition for periodontitis: ≥1 tooth with PPD ≥6 mm 16](#_Toc167130844)

[Cox regression model T1D with and without periodontitis (outcome: retinopathy) 16](#_Toc167130845)

[Cox regression model T1D with and without periodontitis (outcome: albuminuria) 16](#_Toc167130846)

[Cox regression model T1D with and without periodontitis (outcome: ischemic heart disease) 17](#_Toc167130847)

[Cox regression model T1D with and without periodontitis (outcome: stroke) 17](#_Toc167130848)

[Cox regression model T1D with and without periodontitis (outcome: death) 18](#_Toc167130849)

[Cox regression model T2D with and without periodontitis (outcome: retinopathy) 18](#_Toc167130850)

[Cox regression model T2D with and without periodontitis (outcome: albuminuria) 18](#_Toc167130851)

[Cox regression model T2D with and without periodontitis (outcome: ischemic heart disease) 19](#_Toc167130852)

[Cox regression model T2D with and without periodontitis (outcome: stroke) 19](#_Toc167130853)

[Cox regression model T2D with and without periodontitis (outcome: death) 19](#_Toc167130854)

[Number of teeth with PPD ≥6 mm 20](#_Toc167130855)

[Cox regression model T1D with and without periodontitis (outcome: retinopathy) 20](#_Toc167130856)

[Cox regression model T1D with and without periodontitis (outcome: albuminuria) 20](#_Toc167130857)

[Cox regression model T1D with and without periodontitis (outcome: ischemic heart disease) 20](#_Toc167130858)

[Cox regression model T1D with and without periodontitis (outcome: stroke) 21](#_Toc167130859)

[Cox regression model T1D with and without periodontitis (outcome: death) 21](#_Toc167130860)

[Cox regression model T2D with and without periodontitis (outcome: retinopathy) 21](#_Toc167130861)

[Cox regression model T2D with and without periodontitis (outcome: albuminuria) 22](#_Toc167130862)

[Cox regression model T2D with and without periodontitis (outcome: ischemic heart disease) 22](#_Toc167130863)

[Cox regression model T2D with and without periodontitis (outcome: stroke) 23](#_Toc167130864)

[Cox regression model T2D with and without periodontitis (outcome: death) 23](#_Toc167130865)

[Number of teeth 23](#_Toc167130866)

[Cox regression model T1D with and without periodontitis (outcome: retinopathy) 23](#_Toc167130867)

[Cox regression model T1D with and without periodontitis (outcome: albuminuria) 24](#_Toc167130868)

[Cox regression model T1D with and without periodontitis (outcome: ischemic heart disease) 24](#_Toc167130869)

[Cox regression model T1D with and without periodontitis (outcome: stroke) 24](#_Toc167130870)

[Cox regression model T1D with and without periodontitis (outcome: death) 25](#_Toc167130871)

[Cox regression model T2D with and without periodontitis (outcome: retinopathy) 25](#_Toc167130872)

[Cox regression model T2D with and without periodontitis (outcome: albuminuria) 25](#_Toc167130873)

[Cox regression model T2D with and without periodontitis (outcome: ischemic heart disease) 26](#_Toc167130874)

[Cox regression model T2D with and without periodontitis (outcome: stroke) 26](#_Toc167130875)

[Cox regression model T2D with and without periodontitis (outcome: death) 26](#_Toc167130876)

[Diabetes and periodontitis 28](#_Toc167130877)

[Table A9. Prevalence of periodontitis by age category and gender (comparing T1D to no diabetes) 28](#_Toc167130878)

[Figure A1. Prevalence of periodontitis (2010-2020) in subjects with T1D and matched controls without diabetes, females and males by age category. 29](#_Toc167130879)

[Logistic regression model T1D versus matched controls without diabetes (outcome: periodontitis) 29](#_Toc167130880)

[Figure A2. Probability estimates for periodontitis and their contrasts (based on logistic regression, stratified by gender), females and males by age category. 30](#_Toc167130881)

[Table A10. Prevalence of periodontitis by age category and gender (comparing T2D to no diabetes) 31](#_Toc167130882)

[Figure A3. Prevalence of periodontitis (2010-2020) in T2D and matched controls without diabetes, females and males by age category. 32](#_Toc167130883)

[Logistic regression model T2D versus matched controls without diabetes (outcome: periodontitis) 32](#_Toc167130884)

[Figure A4. Probability estimates for periodontitis and their contrasts (based on logistic regression, stratified by gender), females and males by age category. 33](#_Toc167130885)

[Figure A5. Prevalence of periodontitis (2010-2020) in subjects with T1D with good/poor glycemic control and matched controls without diabetes, females and males by age category. 34](#_Toc167130886)

[Logistic regression model T1D with good/poor glycemic control versus matched controls without diabetes (outcome: periodontitis) 34](#_Toc167130887)

[Table A11. Risk ratios (T1D with good/poor glycemic control versus matched controls without diabetes) 35](#_Toc167130888)

[Figure A6. Probability estimates for periodontitis (based on logistic regression, stratified by gender), females and males by age category. 35](#_Toc167130889)

[Logistic regression model T1D according to median yearly HbA1c (outcome: periodontitis) 35](#_Toc167130890)

[Figure A7. Prevalence of periodontitis (2010-2020) in T2D with good/poor glycemic control and matched controls without diabetes, females and males by age category. 37](#_Toc167130891)

[Logistic regression model T2D with good/poor glycemic control versus matched controls without diabetes (outcome: periodontitis) 37](#_Toc167130892)

[Table A12. Risk ratios (T2D with good/poor glycemic control versus matched controls without diabetes) 38](#_Toc167130893)

[Figure A8. Probability estimates for periodontitis (based on logistic regression, stratified by gender), females and males by age category. 38](#_Toc167130894)

[Logistic regression model T2D according to median yearly HbA1c (outcome: periodontitis) 38](#_Toc167130895)

[Diabetes and tooth loss 40](#_Toc167130896)

[Figure A9. Prevalence of tooth loss (2010-2020) in T1D and matched controls without diabetes, females and males by age category. 40](#_Toc167130897)

[Poisson regression model T1D versus matched controls without diabetes (outcome: tooth loss) 40](#_Toc167130898)

[Multinomial logistic regression model T1D versus matched controls without diabetes (outcome: tooth loss, categorical - no extraction, 1-4 extractions, ≥5 extractions) 41](#_Toc167130899)

[Figure A10. Estimated annual incidence rate of tooth loss (based on Poisson regression, stratified by gender) and probability estimates for tooth loss (based on multinomial logistic regression, stratified by gender) in T1D and controls without diabetes, females and males by age category. 42](#_Toc167130900)

[Figure A11. Prevalence of tooth loss (2010-2020) in T2D and matched controls without diabetes, females and males by age category. 43](#_Toc167130901)

[Poisson regression model T2D versus matched controls without diabetes (outcome: tooth loss) 43](#_Toc167130902)

[Multinomial logistic regression model T2D versus matched controls without diabetes (outcome: tooth loss, categorical - no extraction, 1-4 extractions, ≥5 extractions) 44](#_Toc167130903)

[Figure A12. Estimated annual incidence rate of tooth loss (based on Poisson regression, stratified by gender) and probability estimates for tooth loss (based on multinomial logistic regression, stratified by gender) in T2D and controls without diabetes, females and males by age category. 45](#_Toc167130904)

[Figure A13. Prevalence of tooth loss (2010-2020) in T1D with good/poor glycemic control and matched controls without diabetes, females and males by age category. 46](#_Toc167130905)

[Poisson regression model T1D with good/poor glycemic control versus matched controls without diabetes (outcome: tooth loss) 46](#_Toc167130906)

[Multinomial logistic regression model T1D with good/poor glycemic control versus matched controls without diabetes (outcome: tooth loss, categorical - no extraction, 1-4 extractions, ≥5 extractions) 47](#_Toc167130907)

[Figure A14. Probability estimates for tooth loss (based on multinomial logistic regression) in T1D with good/poor glycemic control and matched controls without diabetes, by age category. 48](#_Toc167130908)

[Figure A15. Estimated annual incidence rate of tooth loss (based on Poisson regression, stratified by gender) in T1D with good/poor glycemic control and matched controls without diabetes, females and males by age category. 48](#_Toc167130909)

[Figure A16. Prevalence of tooth loss (2010-2020) in T2D with good/poor glycemic control and matched controls without diabetes, females and males by age category. 49](#_Toc167130910)

[Poisson regression model T2D with good/poor glycemic control versus matched controls without diabetes (outcome: tooth loss) 49](#_Toc167130911)

[Multinomial logistic regression model T2D with good/poor glycemic control versus matched controls without diabetes (outcome: tooth loss, categorical - no extraction, 1-4 extractions, ≥5 extractions) 50](#_Toc167130912)

[Figure A17. Probability estimates for tooth loss (based on multinomial logistic regression, stratified by gender) in T2D with good/poor glycemic control and matched controls without diabetes, by age category. 51](#_Toc167130913)

[Figure A18. Estimated annual incidence rate of tooth loss (based on Poisson regression, stratified by gender) in T2D with good/poor glycemic control and matched controls without diabetes, females and males by age category. 51](#_Toc167130914)

[Diabetes-related complications in T1D 52](#_Toc167130915)

[Table A13. Diabetes-related complications in individuals with Type 1 Diabetes (comparing periodontitis to no periodontitis). 52](#_Toc167130916)

[Retinopathy 53](#_Toc167130917)

[Figure A19. Prevalence of retinopathy (2010-2020) in T1D with and without periodontitis, females and males by age category (excluding individuals deceased over the observation period). 53](#_Toc167130918)

[Cox regression model T1D with and without periodontitis (outcome: retinopathy) 53](#_Toc167130919)

[Figure A20. Retinopathy in T1D with and without periodontitis. Cohort-adjusted rates by age and IRR for females and males, based on age-period-cohort models (truncated at 30-60 years and period 2011-2019). 53](#_Toc167130920)

[Albuminuria 54](#_Toc167130921)

[Figure A21. Prevalence of albuminuria (2010-2020) in T1D with and without periodontitis, females and males by age category (excluding individuals deceased over the observation period). 54](#_Toc167130922)

[Cox regression model T1D with and without periodontitis (outcome: albuminuria) 54](#_Toc167130923)

[Figure A22. Albuminuria in T1D with and without periodontitis. Cohort-adjusted rates by age and IRR for females and males, based on age-period-cohort models (truncated at 30-60 years and period 2011-2019). 54](#_Toc167130924)

[Ischemic heart disease 55](#_Toc167130925)

[Figure A23. Prevalence of ischemic heart disease (2010-2020) in T1D with and without periodontitis, females and males by age category (excluding individuals deceased over the observation period). 55](#_Toc167130926)

[Cox regression model T1D with and without periodontitis (outcome: ischemic heart disease) 55](#_Toc167130927)

[Figure A24. Ischemic heart disease in T1D with and without periodontitis. Cohort-adjusted rates by age and IRR for females and males, based on age-period-cohort models (truncated at 30-60 years and period 2011-2019). 55](#_Toc167130928)

[Stroke 56](#_Toc167130929)

[Figure A25. Prevalence of stroke (2010-2020) in T1D with and without periodontitis, females and males by age category (excluding individuals deceased over the observation period). 56](#_Toc167130930)

[Cox regression model T1D with and without periodontitis (outcome: stroke) 56](#_Toc167130931)

[Figure A26. Stroke in T1D with and without periodontitis. Cohort-adjusted rates by age and IRR for females and males, based on age-period-cohort models (truncated at 30-60 years and period 2011-2019). 56](#_Toc167130932)

[Mortality 57](#_Toc167130933)

[Figure A27. Mortality (2010-2020) in T1D with and without periodontitis, females and males by age category. 57](#_Toc167130934)

[Cox regression model T1D with and without periodontitis (outcome: death) 57](#_Toc167130935)

[Figure A28. Mortality in T1D with and without periodontitis. Cohort-adjusted rates by age and IRR for females and males, based on age-period-cohort models (truncated at 50-70 years and period 2011-2019) (left). Main cause of mortality in T1D, females and males (right). 57](#_Toc167130936)

[Diabetes-related complications in T2D 58](#_Toc167130937)

[Table A14. Diabetes-related complications in individuals with Type 2 Diabetes (comparing periodontitis to no periodontitis). 58](#_Toc167130938)

[Retinopathy 59](#_Toc167130939)

[Figure A29. Prevalence of retinopathy (2010-2020) in T2D with and without periodontitis, females and males by age category (excluding individuals deceased over the observation period). 59](#_Toc167130940)

[Cox regression model T2D with and without periodontitis (outcome: retinopathy) 59](#_Toc167130941)

[Figure A30. Retinopathy in T2D with and without periodontitis. Cohort-adjusted rates by age and IRR for females and males, based on age-period-cohort models (truncated at 30-60 years and period 2011-2019). 59](#_Toc167130942)

[Albuminuria 60](#_Toc167130943)

[Figure A31. Prevalence of albuminuria (2010-2020) in T2D with and without periodontitis, females and males by age category (excluding individuals deceased over the observation period). 60](#_Toc167130944)

[Cox regression model T2D with and without periodontitis (outcome: albuminuria) 60](#_Toc167130945)

[Figure A32. Albuminuria in T2D with and without periodontitis. Cohort-adjusted rates by age and IRR for females and males, based on age-period-cohort models (truncated at 30-60 years and period 2011-2019). 60](#_Toc167130946)

[Ischemic heart disease 61](#_Toc167130947)

[Figure A33. Prevalence of ischemic heart disease (2010-2020) in T2D with and without periodontitis, females and males by age category (excluding individuals deceased over the observation period). 61](#_Toc167130948)

[Cox regression model T2D with and without periodontitis (outcome: ischemic heart disease) 61](#_Toc167130949)

[Figure A34. Ischemic heart disease in T2D with and without periodontitis. Cohort-adjusted rates by age and IRR for females and males, based on age-period-cohort models (truncated at 30-60 years and period 2011-2019). 61](#_Toc167130950)

[Stroke 62](#_Toc167130951)

[Figure A35. Prevalence of stroke (2010-2020) in T2D with and without periodontitis, females and males by age category (excluding individuals deceased over the observation period). 62](#_Toc167130952)

[Cox regression model T2D with and without periodontitis (outcome: stroke) 62](#_Toc167130953)

[Figure A36. Stroke in T2D with and without periodontitis. Cohort-adjusted rates by age and IRR for females and males, based on age-period-cohort models (truncated at 30-60 years and period 2011-2019). 62](#_Toc167130954)

[Mortality 63](#_Toc167130955)

[Figure A37. Mortality (2010-2020) in T2D with and without periodontitis, females and males by age category. 63](#_Toc167130956)

[Cox regression model T2D with and without periodontitis (outcome: death) 63](#_Toc167130957)

[Figure A38. Mortality in T2D with and without periodontitis. Cohort-adjusted rates by age and IRR for females and males, based on age-period-cohort models (truncated at 50-70 years and period 2011-2019) (left). Main cause of mortality in T2D, females and males (right). 63](#_Toc167130958)

# Data retrieval from national registries

^1^Statistics Sweden; ^2^Swedish National Board of Health and Welfare (authority responsible for matching the registries through the national personal identity number); ^3^Region Västra Götaland; ^4^Region Värmland

# Additional variable description

In the 2017 World Workshop on the Classification of Periodontal and Peri-Implant Diseases and Conditions, a **periodontitis** **case** was defined by interdental clinical attachment loss at ≥2 non-adjacent teeth. The case definition for periodontitis in this study was based on periodontal probing depth (PPD) only, due to the low degree of completeness for data on clinical attachment levels in SKaPa. We chose a threshold of ≥3 teeth with PPD ≥6 mm in an attempt to approximate the definition from the World Workshop.

*Papapanou PN, Sanz M, et al. Periodontitis: Consensus report of Workgroup 2 of the 2017 World Workshop on the Classification of Periodontal and Peri-Implant Diseases and Conditions. J Periodontol. 2018; 89(Suppl 1): S173–S182.*

Many epidemiological studies have utilized case definitions based on “deep” PPD (≥6 mm). For instance, 50 of 72 studies included in the widely cited systematic review by Kassebaum et al. (2014) on the global burden of severe periodontitis based their case definitions exclusively on PPD (CPI 4 or PPD ≥6 mm).

*Kassebaum NJ, Bernabé E, Dahiya M, Bhandari B, Murray CJL, Marcenes W. Global Burden of Severe Periodontitis in 1990-2010: A Systematic Review and Meta-regression. Journal of Dental Research. 2014;93(11):1045-1053.*

In this Appendix we provide sensitivity analysis based on an alternative case definition for periodontitis (≥1 tooth with PPD ≥6 mm), as well as a continuous measure of the extent of periodontitis (number of teeth with PPD ≥6 mm).

**Age** (in 2010) was categorized as follows: 18-29 years (born 1981-1992), 30-39 years (born 1971-1980), 40-49 years (born 1961-1970), 50-59 years (born 1951-1960), 60-69 years (born 1941-1950), and ≥70 years (born before 1940).

Information on the **level of education** was obtained from the Longitudinal Integrated Database for Health Insurance and Labour Market Studies (LISA). Individuals were then classified according to the maximum level of education obtained by year 2019:

1. Up to Lower secondary education.
2. Upper secondary to Post-secondary education <2 years.
3. Post-secondary ≥2 years to Tertiary education.

Annual income included salary, pension and capital gain (obtained from LISA). For categorization purposes, we used a ratio calculated relative to a yearly national reference amount*. Ranking was then performed by age for each year (2005-2019). The lowest income subgroup consisted of individuals who ranked within the lowest 5 percentile. Number of years within this lowest income subgroup was used as a measure of deprivation ("**income**”).

* Information obtained from: Statistics Sweden (SCB) 2023, Prisbasbelopp, last accessed 17^th^ august 2023 (<https://www.scb.se/hitta-statistik/statistik-efter-amne/priser-och-konsumtion/konsumentprisindex/konsumentprisindex-kpi/pong/tabell-och-diagram/prisbasbelopp/prisbasbelopp/>)

Data on **systemic conditions** originate from the National Patient Register, including in-patient care and specialist care in Sweden. The register does not cover primary care.

Certain infectious and parasitic diseases (A00-B99)

Neoplasms (C00-D48)

Cancer (C00-C97)

Diseases of the blood and blood-forming organs and certain disorders involving the immune mechanism (D50-D89)

Endocrine, nutritional and metabolic diseases (E00-E90)

Obesity (E66)

Mental and behavioral disorders (F00-F99)

Diseases of the nervous system (G00-G99)

Diseases of the eye and adnexa (H00-H59)

Diseases of the ear and mastoid process (H60-H95)

Diseases of the circulatory system (I00-I99)

**Ischemic heart diseases** (I20-I25)

**Stroke** (I60, I61, I63, I64, G45)

Diseases of the respiratory system (J00-J99)

Diseases of the digestive system (K00-K93)

Diseases of the skin and subcutaneous tissue (L00-L99)

Diseases of the musculoskeletal system and connective tissue (M00-M99)

Diseases of the genitourinary system (N00-N99)

Nephritis, nephrotic syndrome and nephrosis (N00-N07, N17-N19, N25-N27)

For individuals with **diabetes**, year of **onset** was retrieved from NDR. When missing, the first registration in NDR was used instead.

For both T1D and T2D, the yearly maximum **HbA1c** score was noted for the time period 2010-2020 (obtained from NDR). The median yearly HbA1c over the observation period was chosen to represent the individual. Within those individuals with HbA1c data for ≥5 years, subgroups by glycemic control were defined as follows:

- **Good glycemic control**: maximum yearly HbA1c <52 mmol/mol for ≥75% of their observation period.
- **Poor glycemic control**: maximum yearly HbA1c >62 mmol/mol for ≥75% of their observation period.

For these subgroups, information on **smoking** habits, **BMI** and **physical exercise** were obtained from NDR. An individual was classified as smoker if they had reported smoking daily at least once between 2005 and 2020. Non-smoking, non-daily smoking and previous smoking were classified as non-smoking. Maximum BMI and median physical exercise (dichotomized to < or ≥3 times/week; each occasion being the equivalent to a 30-minute walk) between 2005 and 2020 was chosen to represent the individual.

**Tooth loss** (extractions) was considered as both a continuous variable and categorized into 0, 1-4 or ≥5 extractions over the observation period (data obtained from SKaPa).

**Follow-up** represents number of years from the first to the last registration in SKaPa between 2010 and 2020. For the purpose of the Poisson regression analyses for tooth loss, individuals with data from one year only were given a follow-up period value of 0.1 years.

# Table A1. Included individuals with T1D and matched controls without diabetes.

|  | Group | | | |
| --- | --- | --- | --- | --- |
|  | No Diabetes | | Type 1 Diabetes | |
| **Gender** |  |  |  |  |
| Female | 26 271 | 45.4% | 13 022 | 45.2% |
| Male | 31 568 | 54.6% | 15 779 | 54.8% |
| **Age in 2010** | 42.9 | (16.9) | 42.4 | (16.5) |
| **Year of birth** |  |  |  |  |
| 1981-1992 | 15 984 | 27.6% | 8 071 | 28.0% |
| 1971-1980 | 10 066 | 17.4% | 5 134 | 17.8% |
| 1961-1970 | 11 227 | 19.4% | 5 757 | 20.0% |
| 1951-1960 | 9 253 | 16.0% | 4 635 | 16.1% |
| 1941-1950 | 7 545 | 13.0% | 3 571 | 12.4% |
| ≤1940 | 3 764 | 6.5% | 1 633 | 5.7% |
| **Birthplace** |  |  |  |  |
| Sweden | 51 397 | 88.9% | 26 689 | 92.7% |
| Scandinavia (excl. Sweden) | 1 405 | 2.4% | 597 | 2.1% |
| Europe (excl. Scandinavia) | 2 100 | 3.6% | 589 | 2.0% |
| Other | 2 937 | 5.1% | 922 | 3.2% |
| Unknown | 0 | 0.0% | 1 | 0.0% |
| **National area according to NUTS 2** |  |  |  |  |
| SE11 Stockholm | 8 774 | 15.2% | 4 495 | 15.6% |
| SE12 East Middle Sweden | 10 718 | 18.5% | 5 318 | 18.5% |
| SE21 Småland and the islands | 5 989 | 10.4% | 2 992 | 10.4% |
| SE22 South Sweden | 7 832 | 13.5% | 3 989 | 13.9% |
| SE23 West Sweden | 11 901 | 20.6% | 5 804 | 20.2% |
| SE31 North Middle Sweden | 6 794 | 11.7% | 3 254 | 11.3% |
| SE32 Middle Norrland | 2 407 | 4.2% | 1 242 | 4.3% |
| SE33 Upper Norrland | 3 424 | 5.9% | 1 707 | 5.9% |
| **Education** (latest available) |  |  |  |  |
| Up to Lower secondary education | 7 818 | 13.6% | 4 083 | 14.2% |
| Upper secondary to Post-secondary education <2 years | 29 831 | 51.8% | 15 214 | 53.1% |
| Post-secondary ≥2 years to Tertiary education | 19 965 | 34.7% | 9 362 | 32.7% |
| **Annual Income** (SEK; latest available) | 300 900 | [275 600] | 266 700 | [271 600] |
| **Years in lowest 5^th^ percentile of income** (2005-2019) | 0.7 | (1.9) | 0.8 | (2.0) |
| 0 | 45 123 | 78.0% | 22 128 | 76.8% |
| 1-4 years | 9 923 | 17.2% | 5 081 | 17.6% |
| ≥5 years | 2 793 | 4.8% | 1 589 | 5.5% |
| **Systemic conditions** (2005-2020)* |  |  |  |  |
| Certain infectious and parasitic diseases (A00-B99) | 1 702 | 2.9% | 10 768 | 37.4% |
| Neoplasms (C00-D48) | 15 418 | 26.7% | 10 423 | 36.2% |
| Cancer (C00-C97) | 6 079 | 10.5% | 2 684 | 9.3% |
| Diseases of the blood and blood-forming organs [...] (D50-D89) | 0 | 0.0% | 1 | 0.0% |
| Endocrine, nutritional and metabolic diseases (E00-E90) | 7 910 | 13.7% | 28 664 | 99.5% |
| Obesity (E66) | 1 761 | 3.0% | 2 205 | 7.7% |
| Mental and behavioral disorders (F00-F99) | 25 | 0.0% | 300 | 1.0% |
| Diseases of the nervous system (G00-G99) | 0 | 0.0% | 3 | 0.0% |
| Diseases of the eye and adnexa (H00-H59) | 0 | 0.0% | 6 | 0.0% |
| Diseases of the ear and mastoid process (H60-H95) | 0 | 0.0% | 0 | 0.0% |
| Diseases of the circulatory system (I00-I99) | 14 314 | 24.7% | 14 465 | 50.2% |
| Ischemic heart diseases (I20-I25) | 2 648 | 4.6% | 3 000 | 10.4% |
| Stroke (I60, I61, I63, I64, G45) | 1 298 | 2.2% | 1 186 | 4.1% |
| Diseases of the respiratory system (J00-J99) | 8 | 0.0% | 11 | 0.0% |
| Diseases of the digestive system (K00-K93) | 1 | 0.0% | 10 | 0.0% |
| Diseases of the skin and subcutaneous tissue (L00-L99) | 1 | 0.0% | 5 | 0.0% |
| Diseases of the musculoskeletal system and connective tissue (M00-M99) | 5 | 0.0% | 5 | 0.0% |
| Diseases of the genitourinary system (N00-N99) | 1 | 0.0% | 9 | 0.0% |
| Nephritis, nephrotic syndrome and nephrosis (N00-N07, N17-N19, N25-N27) | 1 | 0.0% | 8 | 0.0% |
| **Glycemic control**** |  |  |  |  |
| Good glycemic control | - | - | 1 761 | - |
| Poor glycemic control | - | - | 6 986 | - |
| **Smoking***** | - | - | 3 703 | 13.0% |
| * Systemic conditions exclude diagnoses only registered in primary care. ICD10 codes provided in parentheses. Categorical data is presented as frequencies and percentages. The continuous variables age and number of years in the lowest 5^th^ percentile of income are presented as mean (standard deviation); income is presented as median [interquartile range].  **T1D with ≥5 years HbA1c data, only  *** Smoking data extracted from NDR; information missing for 293 individuals in T1D | | | | |

# Table A2. Subgroups of included individuals with T1D according to glycemic control and their matched controls without diabetes.

|  | No Diabetes | | Type 1 Diabetes | | | |
| --- | --- | --- | --- | --- | --- | --- |
|  |  |  | Good glycemic control | | Poor glycemic control | |
| **Gender** |  |  |  |  |  |  |
| Female | 7 851 | 46.1% | 684 | 38.8% | 3 345 | 47.9% |
| Male | 9 195 | 53.9% | 1 077 | 61.2% | 3 641 | 52.1% |
| **Age in 2010** | 41.9 | (15.8) | 40.6 | (15.5) | 42.6 | (16.1) |
| **Year of birth** |  |  |  |  |  |  |
| 1981-1992 | 4 680 | 27.5% | 545 | 30.9% | 1 816 | 26.0% |
| 1971-1980 | 3 145 | 18.5% | 372 | 21.1% | 1 211 | 17.3% |
| 1961-1970 | 3 730 | 21.9% | 305 | 17.3% | 1 569 | 22.5% |
| 1951-1960 | 2 748 | 16.1% | 264 | 15.0% | 1 210 | 17.3% |
| 1941-1950 | 1 992 | 11.7% | 228 | 12.9% | 809 | 11.6% |
| ≤1940 | 751 | 4.4% | 47 | 2.7% | 371 | 5.3% |
| **Birthplace** |  |  |  |  |  |  |
| Sweden | 15 179 | 89.0% | 1 628 | 92.4% | 6 427 | 92.0% |
| Scandinavia (excl. Sweden) | 386 | 2.3% | 39 | 2.2% | 154 | 2.2% |
| Europe (excl. Scandinavia) | 625 | 3.7% | 41 | 2.3% | 146 | 2.1% |
| Other | 856 | 5.0% | 53 | 3.0% | 259 | 3.7% |
| Unknown | 0 | 0.0% | 0 | 0.0% | 0 | 0.0% |
| **National area according to NUTS 2** |  |  |  |  |  |  |
| SE11 Stockholm | 2 266 | 13.3% | 302 | 17.1% | 953 | 13.6% |
| SE12 East Middle Sweden | 3 275 | 19.2% | 246 | 14.0% | 1 388 | 19.9% |
| SE21 Småland and the islands | 1 889 | 11.1% | 154 | 8.7% | 824 | 11.8% |
| SE22 South Sweden | 2 464 | 14.5% | 277 | 15.7% | 998 | 14.3% |
| SE23 West Sweden | 3 394 | 19.9% | 427 | 24.2% | 1 247 | 17.8% |
| SE31 North Middle Sweden | 1 997 | 11.7% | 149 | 8.5% | 856 | 12.3% |
| SE32 Middle Norrland | 737 | 4.3% | 97 | 5.5% | 273 | 3.9% |
| SE33 Upper Norrland | 1 024 | 6.0% | 109 | 6.2% | 447 | 6.4% |
| **Education (latest available)** |  |  |  |  |  |  |
| Up to Lower secondary education | 2 159 | 12.7% | 147 | 8.4% | 1 256 | 18.1% |
| Upper secondary to Post-secondary education <2 years | 8 986 | 52.9% | 769 | 43.8% | 4 130 | 59.5% |
| Post-secondary ≥2 years to Tertiary education | 5 835 | 34.4% | 840 | 47.8% | 1 561 | 22.5% |
| **Annual Income** (SEK; latest available) | 311 900 | [275 300] | 342 200 | [304 100] | 219 550 | [257 800] |
| **Years in lowest 5th percentile of income** (2005-2019) | 0.7 | (1.9) | 0.5 | (1.4) | 1.0 | (2.4) |
| 0 | 13 217 | 77.5% | 1 453 | 82.5% | 5 009 | 71.7% |
| 1-4 years | 2 978 | 17.5% | 260 | 14.8% | 1 396 | 20.0% |
| ≥5 years | 851 | 5.0% | 48 | 2.7% | 581 | 8.3% |
| **Systemic conditions** (2005-2020) |  |  |  |  |  |  |
| Certain infectious and parasitic diseases (A00-B99) | 442 | 2.6% | 416 | 23.6% | 3 308 | 47.4% |
| Neoplasms (C00-D48) | 4 388 | 25.7% | 569 | 32.3% | 2 848 | 40.8% |
| Cancer (C00-C97) | 1 586 | 9.3% | 139 | 7.9% | 614 | 8.8% |
| Diseases of the blood and blood-forming organs [...] (D50-D89) | 0 | 0.0% | 0 | 0.0% | 0 | 0.0% |
| Endocrine, nutritional and metabolic diseases (E00-E90) | 2 196 | 12.9% | 1 750 | 99.4% | 6 981 | 99.9% |
| Obesity (E66) | 539 | 3.2% | 44 | 2.5% | 810 | 11.6% |
| Mental and behavioral disorders (F00-F99) | 6 | 0.0% | 12 | 0.7% | 108 | 1.5% |
| Diseases of the nervous system (G00-G99) | 0 | 0.0% | 0 | 0.0% | 2 | 0.0% |
| Diseases of the eye and adnexa (H00-H59) | 0 | 0.0% | 0 | 0.0% | 4 | 0.1% |
| Diseases of the ear and mastoid process (H60-H95) | 0 | 0.0% | 0 | 0.0% | 0 | 0.0% |
| Diseases of the circulatory system (I00-I99) | 3 828 | 22.5% | 686 | 39.0% | 4 042 | 57.9% |
| Ischemic heart diseases (I20-I25) | 647 | 3.8% | 78 | 4.4% | 961 | 13.8% |
| Stroke (I60, I61, I63, I64, G45) | 331 | 1.9% | 27 | 1.5% | 380 | 5.4% |
| Diseases of the respiratory system (J00-J99) | 1 | 0.0% | 1 | 0.1% | 5 | 0.1% |
| Diseases of the digestive system (K00-K93) | 0 | 0.0% | 0 | 0.0% | 2 | 0.0% |
| Diseases of the skin and subcutaneous tissue (L00-L99) | 0 | 0.0% | 0 | 0.0% | 0 | 0.0% |
| Diseases of the musculoskeletal system and connective tissue (M00-M99) | 0 | 0.0% | 0 | 0.0% | 0 | 0.0% |
| Diseases of the genitourinary system (N00-N99) | 0 | 0.0% | 1 | 0.1% | 3 | 0.0% |
| Nephritis, nephrotic syndrome and nephrosis (N00-N07, N17-N19, N25-N27) | 0 | 0.0% | 1 | 0.1% | 2 | 0.0% |
| **Behavior** |  |  |  |  |  |  |
| ***Smoking*** | - | - | 103 | 5.9% | 1 472 | 21.1% |
| ***Physical exercise*** |  |  |  |  |  |  |
| ≤2 times/week | - | - | 538 | 30.6% | 3 702 | 53.2% |
| ≥3 times/week | - | - | 1 218 | 69.4% | 3 259 | 46.8% |
| ***Overweight/obesity*** |  |  |  |  |  |  |
| BMI <25 | - | - | 781 | 44.3% | 1 492 | 21.4% |
| BMI 25-29.9 (Overweight) | - | - | 712 | 40.4% | 2 719 | 38.9% |
| BMI ≥30 (Obesity) | - | - | 268 | 15.2% | 2 775 | 39.7% |

# Table A3. Excluded individuals with T1D and matched controls without diabetes, lacking entry in SKaPa (period: 2010-2020).

|  | Group | | | |
| --- | --- | --- | --- | --- |
|  | No Diabetes | | Type 1 Diabetes | |
| **Gender** |  |  |  |  |
| Female | 25 477 | 42.1% | 13 088 | 42.2% |
| Male | 35 068 | 57.9% | 17 921 | 57.8% |
| **Age in 2010** (mean (SD)) | 51.3 | (18.0) | 52.0 | (18.1) |
| **Year of birth** |  |  |  |  |
| 1981-1992 | 24 447 | 20.7% | 12 237 | 20.5% |
| 1971-1980 | 19 341 | 16.3% | 9 684 | 16.2% |
| 1961-1970 | 22 070 | 18.6% | 11 080 | 18.5% |
| 1951-1960 | 19 753 | 16.7% | 9 976 | 16.7% |
| 1941-1950 | 18 851 | 15.9% | 9 582 | 16.0% |
| ≤1940 | 13 922 | 11.8% | 7 251 | 12.1% |
| **Birthplace** |  |  |  |  |
| Sweden | 49 309 | 81.5% | 27 012 | 87.4% |
| Scandinavia (excl. Sweden) | 2 398 | 4.0% | 1 124 | 3.6% |
| Europe (excl. Scandinavia) | 4 297 | 7.1% | 1 297 | 4.2% |
| Other | 4 509 | 7.5% | 1 479 | 4.8% |
| Unknown | 5 | 0.0% | 3 | 0.0% |
| **National area according to NUTS 2** |  |  |  |  |
| SE11 Stockholm | 23 277 | 19.7% | 11 763 | 19.7% |
| SE12 East Middle Sweden | 20 622 | 17.4% | 10 408 | 17.4% |
| SE21 Småland and the islands | 11 107 | 9.4% | 5 614 | 9.4% |
| SE22 South Sweden | 17 285 | 14.6% | 8 763 | 14.7% |
| SE23 West Sweden | 24 206 | 20.4% | 12 204 | 20.4% |
| SE31 North Middle Sweden | 11 756 | 9.9% | 5 935 | 9.9% |
| SE32 Middle Norrland | 4 524 | 3.8% | 2 285 | 3.8% |
| SE33 Upper Norrland | 5 607 | 4.7% | 2 838 | 4.7% |
| **Education** |  |  |  |  |
| Up to Lower secondary education | 13 042 | 22.4% | 7 029 | 23.6% |
| Upper secondary to Post-secondary education <2 years | 28 477 | 48.9% | 15 061 | 50.5% |
| Post-secondary ≥2 years to Tertiary education | 16 736 | 28.7% | 7 717 | 25.9% |
| **Annual Income** (SEK; median [IQR]) | 212 400 | [267 400] | 180 700 | [243 900] |
| **Years in lowest 5th percentile of income** (2005-2019) | 0.8 | (2.1) | 0.8 | (2.0) |
| 0 | 47 100 | 78.1% | 24 131 | 78.1% |
| 1-4 years | 9 762 | 16.2% | 4 997 | 16.2% |
| ≥5 years | 3 465 | 5.7% | 1 782 | 5.8% |
| **Systemic conditions** (2005-2020) |  |  |  |  |
| Certain infectious and parasitic diseases (A00-B99) | 3 260 | 5.4% | 11 841 | 38.2% |
| Neoplasms (C00-D48) | 18 192 | 30.0% | 12 477 | 40.2% |
| Cancer (C00-C97) | 9 873 | 16.3% | 4 772 | 15.4% |
| Diseases of the blood and blood-forming organs [...] (D50-D89) | 0 | 0.0% | 0 | 0.0% |
| Endocrine, nutritional and metabolic diseases (E00-E90) | 9 858 | 16.3% | 30 653 | 98.9% |
| Obesity (E66) | 1 577 | 2.6% | 1 991 | 6.4% |
| Mental and behavioral disorders (F00-F99) | 61 | 0.1% | 328 | 1.1% |
| Diseases of the nervous system (G00-G99) | 1 | 0.0% | 5 | 0.0% |
| Diseases of the eye and adnexa (H00-H59) | 0 | 0.0% | 2 | 0.0% |
| Diseases of the ear and mastoid process (H60-H95) | 0 | 0.0% | 0 | 0.0% |
| Diseases of the circulatory system (I00-I99) | 20 609 | 34.0% | 19 771 | 63.8% |
| Ischemic heart diseases (I20-I25) | 4 694 | 7.8% | 6 073 | 19.6% |
| Stroke (I60, I61, I63, I64, G45) | 2 798 | 4.6% | 2 684 | 8.7% |
| Diseases of the respiratory system (J00-J99) | 24 | 0.0% | 13 | 0.0% |
| Diseases of the digestive system (K00-K93) | 0 | 0.0% | 9 | 0.0% |
| Diseases of the skin and subcutaneous tissue (L00-L99) | 15 | 0.0% | 15 | 0.0% |
| Diseases of the musculoskeletal system and connective tissue (M00-M99) | 9 | 0.0% | 11 | 0.0% |
| Diseases of the genitourinary system (N00-N99) | 1 | 0.0% | 3 | 0.0% |
| Nephritis, nephrotic syndrome and nephrosis (N00-N07, N17-N19, N25-N27) | 1 | 0.0% | 3 | 0.0% |
| **Glycemic control** (T1D with ≥5 years HbA1c data, only) |  |  |  |  |
| Good glycemic control | - | - | 1 324 | - |
| Poor glycemic control | - | - | 6 849 | - |

# Table A4. Included individuals with T2D and matched controls without diabetes.

|  | Group | | | |
| --- | --- | --- | --- | --- |
|  | No Diabetes | | Type 2 Diabetes | |
| **Gender** |  |  |  |  |
| Female | 235 533 | 43.6% | 110 627 | 44.0% |
| Male | 304 272 | 56.4% | 141 018 | 56.0% |
| **Age in 2010** | 60.1 | (13.3) | 60.7 | (13.1) |
| **Year of birth** |  |  |  |  |
| 1981-1992 | 10 348 | 1.9% | 4 217 | 1.7% |
| 1971-1980 | 28 120 | 5.2% | 11 576 | 4.6% |
| 1961-1970 | 75 431 | 14.0% | 33 536 | 13.3% |
| 1951-1960 | 124 714 | 23.1% | 58 104 | 23.1% |
| 1941-1950 | 168 295 | 31.2% | 80 540 | 32.0% |
| ≤1940 | 132 897 | 24.6% | 63 672 | 25.3% |
| **Birthplace** |  |  |  |  |
| Sweden | 478 632 | 88.7% | 203 514 | 80.9% |
| Scandinavia (excl. Sweden) | 21 928 | 4.1% | 11 253 | 4.5% |
| Europe (excl. Scandinavia) | 19 890 | 3.7% | 13 279 | 5.3% |
| Other | 19 334 | 3.6% | 23 527 | 9.4% |
| Unknown | 6 | 0.0% | 12 | 0.0% |
| **National area according to NUTS 2** |  |  |  |  |
| SE11 Stockholm | 76 258 | 14.1% | 35 020 | 13.9% |
| SE12 East Middle Sweden | 100 629 | 18.6% | 47 765 | 19.0% |
| SE21 Småland and the islands | 55 450 | 10.3% | 25 763 | 10.2% |
| SE22 South Sweden | 66 992 | 12.4% | 31 651 | 12.6% |
| SE23 West Sweden | 104 103 | 19.3% | 47 772 | 19.0% |
| SE31 North Middle Sweden | 74 247 | 13.8% | 34 396 | 13.7% |
| SE32 Middle Norrland | 27 280 | 5.1% | 12 669 | 5.0% |
| SE33 Upper Norrland | 34 846 | 6.5% | 16 609 | 6.6% |
| **Education** (latest available) |  |  |  |  |
| Up to Lower secondary education | 134 919 | 25.1% | 80 780 | 32.4% |
| Upper secondary to Post-secondary education <2 years | 262 874 | 48.9% | 124 894 | 50.2% |
| Post-secondary ≥2 years to Tertiary education | 139 526 | 26.0% | 43 312 | 17.4% |
| **Annual Income** (SEK; latest available) | 197 400 | [202 400] | 168 500 | [133 200] |
| **Years in lowest 5^th^ percentile of income** (2005-2019) | 0.5 | (1.9) | 0.8 | (2.3) |
| 0 | 464 008 | 86.0% | 203 105 | 80.7% |
| 1-4 years | 53 916 | 10.0% | 32 265 | 12.8% |
| ≥5 years | 21 880 | 4.1% | 16 267 | 6.5% |
| **Systemic conditions** (2005-2020)* |  |  |  |  |
| Certain infectious and parasitic diseases (A00-B99) | 37 332 | 6.9% | 35 971 | 14.3% |
| Neoplasms (C00-D48) | 208 763 | 38.7% | 104 478 | 41.5% |
| Cancer (C00-C97) | 121 118 | 22.4% | 57 907 | 23.0% |
| Diseases of the blood and blood-forming organs [...] (D50-D89) | 5 | 0.0% | 5 | 0.0% |
| Endocrine, nutritional and metabolic diseases (E00-E90) | 110 280 | 20.4% | 186 300 | 74.0% |
| Obesity (E66) | 11 641 | 2.2% | 30 423 | 12.1% |
| Mental and behavioral disorders (F00-F99) | 722 | 0.1% | 853 | 0.3% |
| Diseases of the nervous system (G00-G99) | 6 | 0.0% | 14 | 0.0% |
| Diseases of the eye and adnexa (H00-H59) | 11 | 0.0% | 19 | 0.0% |
| Diseases of the ear and mastoid process (H60-H95) | 0 | 0.0% | 0 | 0.0% |
| Diseases of the circulatory system (I00-I99) | 253 533 | 47.0% | 170 748 | 67.9% |
| Ischemic heart diseases (I20-I25) | 64 083 | 11.9% | 56 417 | 22.4% |
| Stroke (I60, I61, I63, I64, G45) | 31 514 | 5.8% | 23 098 | 9.2% |
| Diseases of the respiratory system (J00-J99) | 158 | 0.0% | 108 | 0.0% |
| Diseases of the digestive system (K00-K93) | 18 | 0.0% | 11 | 0.0% |
| Diseases of the skin and subcutaneous tissue (L00-L99) | 50 | 0.0% | 34 | 0.0% |
| Diseases of the musculoskeletal system and connective tissue (M00-M99) | 90 | 0.0% | 48 | 0.0% |
| Diseases of the genitourinary system (N00-N99) | 18 | 0.0% | 34 | 0.0% |
| Nephritis, nephrotic syndrome and nephrosis (N00-N07, N17-N19, N25-N27) | 12 | 0.0% | 24 | 0.0% |
| **Glycemic control**** |  |  |  |  |
| Good glycemic control | - | - | 43 863 | - |
| Poor glycemic control | - | - | 13 559 | - |
| **Smoking***** | - | - | 34 708 | 14.3% |
| * Systemic conditions exclude diagnoses only registered in primary care. ICD10 codes provided in parentheses. Categorical data is presented as frequencies and percentages. The continuous variables age and number of years in the lowest 5^th^ percentile of income are presented as mean (standard deviation); income is presented as median [interquartile range].  **T2D with ≥5 years HbA1c data, only  *** Smoking data extracted from NDR; information missing for 9 568 individuals in T2D | | | | |

# Table A5. Subgroups of included individuals with T2D according to glycemic control and their matched controls without diabetes.

|  | No Diabetes | | Type 2 Diabetes | | | |
| --- | --- | --- | --- | --- | --- | --- |
|  |  |  | Good glycemic control | | Poor glycemic control | |
| **Gender** |  |  |  |  |  |  |
| Female | 51 018 | 46.0% | 20 906 | 47.7% | 5 640 | 41.6% |
| Male | 59 979 | 54.0% | 22 957 | 52.3% | 7 919 | 58.4% |
| **Age in 2010** | 60.8 | (11.7) | 62.5 | (11.2) | 59.4 | (13.0) |
| **Year of birth** |  |  |  |  |  |  |
| 1981-1992 | 1 114 | 1.0% | 304 | 0.7% | 217 | 1.6% |
| 1971-1980 | 4 161 | 3.7% | 1 191 | 2.7% | 628 | 4.6% |
| 1961-1970 | 13 590 | 12.2% | 4 147 | 9.5% | 2 264 | 16.7% |
| 1951-1960 | 26 173 | 23.6% | 9 254 | 21.1% | 3 526 | 26.0% |
| 1941-1950 | 40 511 | 36.5% | 17 324 | 39.5% | 3 833 | 28.3% |
| ≤1940 | 25 448 | 22.9% | 11 643 | 26.5% | 3 091 | 22.8% |
| **Birthplace** |  |  |  |  |  |  |
| Sweden | 98 283 | 88.5% | 36 478 | 83.2% | 10 013 | 73.8% |
| Scandinavia (excl. Sweden) | 4 809 | 4.3% | 2 119 | 4.8% | 654 | 4.8% |
| Europe (excl. Scandinavia) | 4 164 | 3.8% | 2 034 | 4.6% | 964 | 7.1% |
| Other | 3 740 | 3.4% | 3 232 | 7.4% | 1 928 | 14.2% |
| Unknown | 1 | 0.0% | 0 | 0.0% | 0 | 0.0% |
| **National area according to NUTS 2** |  |  |  |  |  |  |
| SE11 Stockholm | 16 267 | 14.7% | 6 317 | 14.4% | 2 030 | 15.0% |
| SE12 East Middle Sweden | 19 221 | 17.3% | 7 946 | 18.1% | 2 290 | 16.9% |
| SE21 Småland and the islands | 11 140 | 10.0% | 4 250 | 9.7% | 1 379 | 10.2% |
| SE22 South Sweden | 14 801 | 13.3% | 5 911 | 13.5% | 1 750 | 12.9% |
| SE23 West Sweden | 23 199 | 20.9% | 9 105 | 20.8% | 2 632 | 19.4% |
| SE31 North Middle Sweden | 15 800 | 14.2% | 6 037 | 13.8% | 2 130 | 15.7% |
| SE32 Middle Norrland | 5 175 | 4.7% | 2 117 | 4.8% | 613 | 4.5% |
| SE33 Upper Norrland | 5 394 | 4.9% | 2 180 | 5.0% | 735 | 5.4% |
| **Education (latest available)** |  |  |  |  |  |  |
| Up to Lower secondary education | 27 587 | 25.0% | 13 889 | 31.9% | 4 804 | 36.1% |
| Upper secondary to Post-secondary education <2 years | 53 962 | 48.8% | 21 450 | 49.3% | 6 768 | 50.8% |
| Post-secondary ≥2 years to Tertiary education | 28 948 | 26.2% | 8 205 | 18.8% | 1 749 | 13.1% |
| **Annual Income** (SEK; latest available) | 193 000 | [182 500] | 170 600 | [109 300] | 149 800 | [129 800] |
| **Years in lowest 5th percentile of income** (2005-2019) | 0.5 | (1.9) | 0.7 | (2.2) | 1.1 | (2.6) |
| 0 | 95 344 | 85.9% | 36 327 | 82.8% | 10 180 | 75.1% |
| 1-4 years | 11 139 | 10.0% | 5 022 | 11.4% | 2 151 | 15.9% |
| ≥5 years | 4 514 | 4.1% | 2 514 | 5.7% | 1 228 | 9.1% |
| **Systemic conditions** (2005-2020) |  |  |  |  |  |  |
| Certain infectious and parasitic diseases (A00-B99) | 7 744 | 7.0% | 4 908 | 11.2% | 3 743 | 27.6% |
| Neoplasms (C00-D48) | 44 217 | 39.8% | 19 090 | 43.5% | 5 671 | 41.8% |
| Cancer (C00-C97) | 25 907 | 23.3% | 10 667 | 24.3% | 2 782 | 20.5% |
| Diseases of the blood and blood-forming organs [...] (D50-D89) | 0 | 0.0% | 0 | 0.0% | 1 | 0.0% |
| Endocrine, nutritional and metabolic diseases (E00-E90) | 23 224 | 20.9% | 32 232 | 73.5% | 12 486 | 92.1% |
| Obesity (E66) | 2 467 | 2.2% | 4 306 | 9.8% | 2 872 | 21.2% |
| Mental and behavioral disorders (F00-F99) | 153 | 0.1% | 112 | 0.3% | 119 | 0.9% |
| Diseases of the nervous system (G00-G99) | 153 | 0.1% | 112 | 0.3% | 119 | 0.9% |
| Diseases of the eye and adnexa (H00-H59) | 2 | 0.0% | 2 | 0.0% | 4 | 0.0% |
| Diseases of the ear and mastoid process (H60-H95) | 0 | 0.0% | 0 | 0.0% | 0 | 0.0% |
| Diseases of the circulatory system (I00-I99) | 53 013 | 47.8% | 30 112 | 68.7% | 10 347 | 76.3% |
| Ischemic heart diseases (I20-I25) | 12 887 | 11.6% | 8 857 | 20.2% | 4 182 | 30.8% |
| Stroke (I60, I61, I63, I64, G45) | 6 373 | 5.7% | 3 565 | 8.1% | 1 670 | 12.3% |
| Diseases of the respiratory system (J00-J99) | 24 | 0.0% | 24 | 0.1% | 7 | 0.1% |
| Diseases of the digestive system (K00-K93) | 3 | 0.0% | 4 | 0.0% | 0 | 0.0% |
| Diseases of the skin and subcutaneous tissue (L00-L99) | 10 | 0.0% | 3 | 0.0% | 7 | 0.1% |
| Diseases of the musculoskeletal system and connective tissue (M00-M99) | 16 | 0.0% | 7 | 0.0% | 2 | 0.0% |
| Diseases of the genitourinary system (N00-N99) | 5 | 0.0% | 2 | 0.0% | 6 | 0.0% |
| Nephritis, nephrotic syndrome and nephrosis (N00-N07, N17-N19, N25-N27) | 4 | 0.0% | 2 | 0.0% | 6 | 0.0% |
| **Behavior** |  |  |  |  |  |  |
| ***Smoking*** | - | - | 6 336 | 14.5% | 2 542 | 18.8% |
| ***Physical exercise*** |  |  |  |  |  |  |
| ≤2 times/week | - | - | 16 977 | 39.1% | 8 364 | 62.4% |
| ≥3 times/week | - | - | 26 396 | 60.9% | 5 050 | 37.6% |
| ***Overweight/obesity*** |  |  |  |  |  |  |
| BMI <25 | - | - | 3 898 | 8.9% | 547 | 4.0% |
| BMI 25-29.9 (Overweight) | - | - | 15 924 | 36.3% | 3 289 | 24.3% |
| BMI ≥30 (Obesity) | - | - | 24 041 | 54.8% | 9 723 | 71.7% |

# Table A6. Excluded individuals with T2D and matched controls without diabetes, lacking entry in SKaPa (period: 2010-2020)

|  | Group | | | |
| --- | --- | --- | --- | --- |
|  | No Diabetes | | Type 2 Diabetes | |
| **Gender** |  |  |  |  |
| Female | 356 182 | 42.6% | 190 662 | 42.4% |
| Male | 479 575 | 57.4% | 259 105 | 57.6% |
| **Age in 2010** (mean (SD)) | 65.7 | (14.0) | 65.4 | (14.2) |
| **Year of birth** |  |  |  |  |
| 1981-1992 | 18 047 | 1.3% | 9 034 | 1.3% |
| 1971-1980 | 55 295 | 4.0% | 27 691 | 3.9% |
| 1961-1970 | 151 557 | 11.0% | 76 064 | 10.8% |
| 1951-1960 | 269 090 | 19.6% | 135 621 | 19.3% |
| 1941-1950 | 409 963 | 29.8% | 208 220 | 29.7% |
| ≤1940 | 471 610 | 34.3% | 244 782 | 34.9% |
| **Birthplace** |  |  |  |  |
| Sweden | 702 041 | 84.0% | 345 345 | 76.9% |
| Scandinavia (excl. Sweden) | 43 694 | 5.2% | 24 436 | 5.4% |
| Europe (excl. Scandinavia) | 51 977 | 6.2% | 36 055 | 8.0% |
| Other | 37 585 | 4.5% | 42 976 | 9.6% |
| Unknown | 82 | 0.0% | 39 | 0.0% |
| **National area according to NUTS 2** |  |  |  |  |
| SE11 Stockholm | 258 179 | 18.8% | 131 385 | 18.7% |
| SE12 East Middle Sweden | 242 751 | 17.6% | 123 691 | 17.6% |
| SE21 Småland and the islands | 127 638 | 9.3% | 65 289 | 9.3% |
| SE22 South Sweden | 193 129 | 14.0% | 99 007 | 14.1% |
| SE23 West Sweden | 267 339 | 19.4% | 135 620 | 19.3% |
| SE31 North Middle Sweden | 151 878 | 11.0% | 77 300 | 11.0% |
| SE32 Middle Norrland | 61 215 | 4.5% | 31 308 | 4.5% |
| SE33 Upper Norrland | 73 433 | 5.3% | 37 812 | 5.4% |
| **Education** |  |  |  |  |
| Up to Lower secondary education | 271 317 | 33.8% | 176 121 | 41.0% |
| Upper secondary to Post-secondary education <2 years | 351 243 | 43.7% | 188 488 | 43.9% |
| Post-secondary ≥2 years to Tertiary education | 180 578 | 22.5% | 64 994 | 15.1% |
| **Annual Income** (SEK; median [IQR]) | 169 900 | [132 600] | 149 000 | [113 200] |
| **Years in lowest 5th percentile of income** (2005-2019) | 0.7 | (2.1) | 0.9 | (2.4) |
| 0 | 700 259 | 84.1% | 358 206 | 79.9% |
| 1-4 years | 88 342 | 10.6% | 57 384 | 12.8% |
| ≥5 years | 44 418 | 5.3% | 32 938 | 7.3% |
| **Glycemic control** (T2D with ≥5 years HbA1c data) |  |  |  |  |
| Good glycemic control | - | - | 59 701 | - |
| Poor glycemic control | - | - | 23 648 | - |
| **Systemic conditions** (2005-2020) |  |  |  |  |
| Certain infectious and parasitic diseases (A00-B99) | 80 605 | 9.6% | 83 346 | 18.5% |
| Neoplasms (C00-D48) | 333 452 | 39.9% | 189 060 | 42.0% |
| Cancer (C00-C97) | 220 372 | 26.4% | 117 584 | 26.1% |
| Diseases of the blood and blood-forming organs [...] (D50-D89) | 21 | 0.0% | 26 | 0.0% |
| Endocrine, nutritional and metabolic diseases (E00-E90) | 191 508 | 22.9% | 352 924 | 78.5% |
| Obesity (E66) | 15 883 | 1.9% | 46 292 | 10.3% |
| Mental and behavioral disorders (F00-F99) | 1 437 | 0.2% | 1 721 | 0.4% |
| Diseases of the nervous system (G00-G99) | 14 | 0.0% | 35 | 0.0% |
| Diseases of the eye and adnexa (H00-H59) | 25 | 0.0% | 27 | 0.0% |
| Diseases of the ear and mastoid process (H60-H95) | 0 | 0.0% | 0 | 0.0% |
| Diseases of the circulatory system (I00-I99) | 454 661 | 54.4% | 331 195 | 73.6% |
| Ischemic heart diseases (I20-I25) | 127 680 | 15.3% | 122 541 | 27.2% |
| Stroke (I60, I61, I63, I64, G45) | 73 365 | 8.8% | 56 533 | 12.6% |
| Diseases of the respiratory system (J00-J99) | 327 | 0.0% | 195 | 0.0% |
| Diseases of the digestive system (K00-K93) | 35 | 0.0% | 17 | 0.0% |
| Diseases of the skin and subcutaneous tissue (L00-L99) | 237 | 0.0% | 145 | 0.0% |
| Diseases of the musculoskeletal system and connective tissue (M00-M99) | 151 | 0.0% | 106 | 0.0% |
| Diseases of the genitourinary system (N00-N99) | 39 | 0.0% | 67 | 0.0% |
| Nephritis, nephrotic syndrome and nephrosis (N00-N07, N17-N19, N25-N27) | 31 | 0.0% | 54 | 0.0% |

# Sensitivity analyses: Periodontitis

## Table A7. Summary of sensitivity analyses (outcome: periodontitis)

|  |  | **T1D versus non-T1D** | **T2D versus non-T2D** |
| --- | --- | --- | --- |
| **Prevalent periodontitis**  (risk ratio (95% CI)) | ≥3 teeth with PPD ≥6 mm | 1.13 (1.09, 1.18) | 1.26 (1.24, 1.27) |
|  | ≥1 tooth with PPD ≥6 mm | 1.07 (1.05, 1.09) | 1.12 (1.11, 1.12) |
| **Maximum extent of periodontitis**  (mean difference (95% CI)) | Number of teeth with PPD ≥6 mm | 0.12 (0.08, 0.15) | 0.36 (0.35, 0.38) |

## Alternative case definition for periodontitis: ≥1 tooth with PPD ≥6 mm

### Logistic regression model T1D versus matched controls without diabetes (outcome: periodontitis)

Logistic regression Number of obs = 85,042

LR chi2(15) = 4896.42

Prob > chi2 = 0.0000

Log likelihood = -48328.034 Pseudo R2 = 0.0482

-----------------------------------------------------------------------------------------------------------------------

everParod1 | Odds ratio Std. err. z P>|z| [95% conf. interval]

------------------------------------------------------+----------------------------------------------------------------

cohort |

18-29 | 1 (base)

30-39 | 1.499471 .0492636 12.33 0.000 1.405959 1.599202

40-49 | 1.976131 .0608892 22.11 0.000 1.860322 2.099149

50-59 | 3.016299 .0942308 35.34 0.000 2.83715 3.206759

60-69 | 3.902143 .1275685 41.65 0.000 3.659955 4.160357

≥70 | 3.587603 .1476423 31.04 0.000 3.309592 3.888967

|

Group |

No Diabetes | 1 (base)

Type 1 Diabetes | 1.13237 .0416591 3.38 0.001 1.053593 1.217036

|

cohort#Group |

30-39#Type 1 Diabetes | 1.043754 .0572982 0.78 0.435 .9372825 1.162321

40-49#Type 1 Diabetes | 1.038522 .0534301 0.73 0.463 .9389073 1.148704

50-59#Type 1 Diabetes | .9697944 .050968 -0.58 0.559 .8748716 1.075016

60-69#Type 1 Diabetes | .9352488 .0518582 -1.21 0.227 .8389368 1.042618

≥70#Type 1 Diabetes | .7379291 .0530133 -4.23 0.000 .6410084 .8495043

|

Kon |

Male | 1 (base)

Female | .7544315 .0120626 -17.62 0.000 .7311557 .7784482

|

Education |

Up to Lower secondary education | 1.170412 .027066 6.80 0.000 1.118548 1.224681

Upper secondary to Post-secondary education <2 years | 1 (base)

Post-secondary ≥2 years to Tertiary education | .8215446 .0148662 -10.86 0.000 .792918 .8512047

|

No_5p_rank | 1.006189 .0042374 1.47 0.143 .9979181 1.014529

_cons | .2256258 .0055071 -61.00 0.000 .2150862 .2366819

-----------------------------------------------------------------------------------------------------------------------

Note: _cons estimates baseline odds.

### Logistic regression model T2D versus matched controls without diabetes (outcome: periodontitis)

Logistic regression Number of obs = 770,672

LR chi2(15) = 17420.11

Prob > chi2 = 0.0000

Log likelihood = -507022.14 Pseudo R2 = 0.0169

-----------------------------------------------------------------------------------------------------------------------

everParod1 | Odds ratio Std. err. z P>|z| [95% conf. interval]

------------------------------------------------------+----------------------------------------------------------------

cohort |

18-29 | 1 (base)

30-39 | 1.445942 .0430251 12.39 0.000 1.364026 1.532777

40-49 | 1.827252 .0500713 22.00 0.000 1.731703 1.928073

50-59 | 2.665167 .0715235 36.53 0.000 2.528607 2.809103

60-69 | 3.457682 .0921707 46.54 0.000 3.281669 3.643136

≥70 | 2.941754 .0790108 40.17 0.000 2.790901 3.10076

|

Group |

No Diabetes | 1 (base)

Type 2 Diabetes | 1.351781 .0621216 6.56 0.000 1.235348 1.479189

|

cohort#Group |

30-39#Type 2 Diabetes | 1.129387 .0589159 2.33 0.020 1.019621 1.25097

40-49#Type 2 Diabetes | 1.137365 .0546202 2.68 0.007 1.035195 1.249619

50-59#Type 2 Diabetes | .9899537 .0466182 -0.21 0.830 .9026735 1.085673

60-69#Type 2 Diabetes | .8420575 .0393789 -3.68 0.000 .7683079 .9228864

≥70#Type 2 Diabetes | .7349303 .0345607 -6.55 0.000 .6702205 .8058878

|

Kon |

Male | 1 (base)

Female | .7826459 .0037675 -50.91 0.000 .7752963 .7900651

|

Education |

Up to Lower secondary education | 1.046709 .0060137 7.95 0.000 1.034989 1.058563

Upper secondary to Post-secondary education <2 years | 1 (base)

Post-secondary ≥2 years to Tertiary education | .8490589 .0051623 -26.91 0.000 .839001 .8592373

|

No_5p_rank | 1.002782 .0012313 2.26 0.024 1.000372 1.005199

_cons | .2538528 .0066969 -51.97 0.000 .2410607 .2673238

-----------------------------------------------------------------------------------------------------------------------

Note: _cons estimates baseline odds.

## Number of teeth with PPD ≥6 mm

### Regression model T1D versus matched controls without diabetes (outcome: periodontitis extent)

Source | SS df MS Number of obs = 85,042

-------------+---------------------------------- F(15, 85026) = 232.30

Model | 19049.8111 15 1269.98741 Prob > F = 0.0000

Residual | 464843.119 85,026 5.46707029 R-squared = 0.0394

-------------+---------------------------------- Adj R-squared = 0.0392

Total | 483892.93 85,041 5.69011336 Root MSE = 2.3382

-----------------------------------------------------------------------------------------------------------------------

perio_numberTeeth | Coefficient Std. err. t P>|t| [95% conf. interval]

------------------------------------------------------+----------------------------------------------------------------

cohort |

18-29 | 0 (base)

30-39 | .2931772 .0300766 9.75 0.000 .2342273 .3521271

40-49 | .5419772 .0292682 18.52 0.000 .4846118 .5993426

50-59 | .9655693 .0311702 30.98 0.000 .904476 1.026663

60-69 | 1.042657 .0335753 31.05 0.000 .97685 1.108465

≥70 | .732808 .0440258 16.64 0.000 .6465179 .8190981

|

Group |

No Diabetes | 0 (base)

Type 1 Diabetes | .1154013 .0322195 3.58 0.000 .0522514 .1785512

|

cohort#Group |

30-39#Type 1 Diabetes | .0304001 .0517101 0.59 0.557 -.0709512 .1317515

40-49#Type 1 Diabetes | .086738 .0500423 1.73 0.083 -.0113446 .1848206

50-59#Type 1 Diabetes | -.0128664 .0533327 -0.24 0.809 -.117398 .0916652

60-69#Type 1 Diabetes | -.0508365 .0578026 -0.88 0.379 -.1641291 .0624562

≥70#Type 1 Diabetes | -.226206 .0771316 -2.93 0.003 -.3773834 -.0750286

|

Kon |

Male | 0 (base)

Female | -.2111529 .0162748 -12.97 0.000 -.2430515 -.1792544

|

Education |

Up to Lower secondary education | .2981926 .0253997 11.74 0.000 .2484094 .3479757

Upper secondary to Post-secondary education <2 years | 0 (base)

Post-secondary ≥2 years to Tertiary education | -.2339976 .0180107 -12.99 0.000 -.2692984 -.1986969

|

No_5p_rank | .0184246 .0043752 4.21 0.000 .0098492 .027

_cons | .5320711 .0220034 24.18 0.000 .4889447 .5751975

-----------------------------------------------------------------------------------------------------------------------

### Regression model T2D versus matched controls without diabetes (outcome: periodontitis extent)

Source | SS df MS Number of obs = 770,672

-------------+---------------------------------- F(15, 770656) = 1004.30

Model | 114074.761 15 7604.98406 Prob > F = 0.0000

Residual | 5835739.3 770,656 7.57243089 R-squared = 0.0192

-------------+---------------------------------- Adj R-squared = 0.0192

Total | 5949814.06 770,671 7.72030355 Root MSE = 2.7518

-----------------------------------------------------------------------------------------------------------------------

perio_numberTeeth | Coefficient Std. err. t P>|t| [95% conf. interval]

------------------------------------------------------+----------------------------------------------------------------

cohort |

18-29 | 0 (base)

30-39 | .2615618 .0319508 8.19 0.000 .1989393 .3241844

40-49 | .4963294 .0291544 17.02 0.000 .4391878 .553471

50-59 | .8244596 .0284651 28.96 0.000 .7686689 .8802504

60-69 | .9596533 .0282087 34.02 0.000 .9043653 1.014941

≥70 | .6401186 .0285268 22.44 0.000 .5842069 .6960302

|

Group |

No Diabetes | 0 (base)

Type 2 Diabetes | .3255561 .0515371 6.32 0.000 .2245452 .4265671

|

cohort#Group |

30-39#Type 2 Diabetes | .3827225 .0600723 6.37 0.000 .2649827 .5004623

40-49#Type 2 Diabetes | .3785032 .0546583 6.92 0.000 .2713748 .4856316

50-59#Type 2 Diabetes | .210149 .0533788 3.94 0.000 .1055282 .3147698

60-69#Type 2 Diabetes | -.0557004 .0528814 -1.05 0.292 -.1593462 .0479454

≥70#Type 2 Diabetes | -.2575913 .0532505 -4.84 0.000 -.3619604 -.1532221

|

Kon |

Male | 0 (base)

Female | -.3675553 .0063701 -57.70 0.000 -.3800404 -.3550701

|

Education |

Up to Lower secondary education | .1187198 .0077342 15.35 0.000 .1035612 .1338785

Upper secondary to Post-secondary education <2 years | 0 (base)

Post-secondary ≥2 years to Tertiary education | -.2338523 .0079466 -29.43 0.000 -.2494274 -.2182772

|

No_5p_rank | .015198 .0016358 9.29 0.000 .0119919 .0184041

_cons | .7215133 .0277463 26.00 0.000 .6671315 .7758951

-----------------------------------------------------------------------------------------------------------------------

# Sensitivity analyses: Complications

## Table A8. Summary of sensitivity analyses (outcome: diabetes-related complications)

|  |  | **Case definition for periodontitis** | | **Number of teeth with PPD ≥6 mm** (continuous) | **Number of teeth at start of observation period** (continuous) |
| --- | --- | --- | --- | --- | --- |
|  |  | **≥3 teeth with PPD ≥6 mm** (categorical, as presented in main text) | **≥1 tooth with PPD ≥6 mm** (categorical) |
| **Complications in T1D**  Periodontitis versus no periodontitis;  hazard ratio (95% CI) | |  |  |  |  |
|  | **Retinopathy** | 1.08 (1.02, 1.14) | 1.08 (1.04, 1.13) | 1.01 (1.01, 1.02) | 1.00 (1.00, 1.00) |
|  | **Albuminuria** | 1.14 (1.06, 1.23) | 1.10 (1.04, 1.17) | 1.02 (1.01, 1.03) | 0.99 (0.98, 0.99) |
|  | **Ischemic heart disease** | 0.96 (0.86, 1.08) | 0.95 (0.87, 1.04) | 0.99 (0.98, 1.01) | 0.98 (0.97, 0.99) |
|  | **Stroke** | 1.05 (0.89, 1.25) | 1.04 (0.91, 1.19) | 1.02 (0.99, 1.04) | 0.98 (0.97, 0.99) |
|  | **Mortality** | 0.91 (0.81, 1.02) | 0.77 (0.70, 0.85) | 0.98 (0.97, 1.00) | 0.97 (0.96, 0.97) |
| **Complications in T2D**  Periodontitis versus no periodontitis;  hazard ratio (95% CI) | |  |  |  |  |
|  | **Retinopathy** | 1.08 (1.06, 1.10) | 1.08 (1.06, 1.09) | 1.01 (1.01, 1.01) | 1.00 (1.00, 1.00) |
|  | **Albuminuria** | 1.09 (1.07, 1.11) | 1.06 (1.05, 1.08) | 1.01 (1.01, 1.02) | 0.99 (0.99, 0.99) |
|  | **Ischemic heart disease** | 0.96 (0.94, 0.99) | 0.93 (0.91, 0.95) | 1.00 (0.99, 1.00) | 0.98 (0.98, 0.98) |
|  | **Stroke** | 0.99 (0.95, 1.03) | 0.95 (0.91, 0.98) | 1.00 (1.00, 1.01) | 0.98 (0.98, 0.99) |
|  | **Mortality** | 0.81 (0.79, 0.83) | 0.77 (0.75, 0.79) | 0.97 (0.97, 0.97) | 0.97 (0.97, 0.97) |

## Alternative case definition for periodontitis: ≥1 tooth with PPD ≥6 mm

### Cox regression model T1D with and without periodontitis (outcome: retinopathy)

Cox regression with Breslow method for ties

No. of subjects = 17,788 Number of obs = 17,788

No. of failures = 12,239

Time at risk = 88,930

LR chi2(10) = 97.35

Log likelihood = -113558.59 Prob > chi2 = 0.0000

----------------------------------------------------------------------------------------------------------------

_t | Haz. ratio Std. err. z P>|z| [95% conf. interval]

-----------------------------------------------+----------------------------------------------------------------

1.everParod1 | 1.08084 .0222172 3.78 0.000 1.038161 1.125274

|

cohort |

30-39 | 1.117403 .0295336 4.20 0.000 1.060992 1.176813

40-49 | 1.133445 .0298741 4.75 0.000 1.07638 1.193536

50-59 | 1.139527 .0335238 4.44 0.000 1.07568 1.207164

60-69 | 1.1706 .0388417 4.75 0.000 1.096895 1.249259

≥70 | 1.188631 .0564402 3.64 0.000 1.083002 1.304563

|

Kon |

Female | .9912268 .0182015 -0.48 0.631 .9561868 1.027551

|

Education |

Up to Lower secondary education | .9815376 .0286141 -0.64 0.523 .9270271 1.039253

Post-secondary ≥2 years to Tertiary education | .9404062 .0189643 -3.05 0.002 .9039617 .9783199

|

No_5p_rank | 1.012804 .0047089 2.74 0.006 1.003617 1.022076

----------------------------------------------------------------------------------------------------------------

### Cox regression model T1D with and without periodontitis (outcome: albuminuria)

Cox regression with Breslow method for ties

No. of subjects = 24,817 Number of obs = 24,817

No. of failures = 5,083

Time at risk = 204,357

LR chi2(10) = 1170.28

Log likelihood = -50054.026 Prob > chi2 = 0.0000

----------------------------------------------------------------------------------------------------------------

_t | Haz. ratio Std. err. z P>|z| [95% conf. interval]

-----------------------------------------------+----------------------------------------------------------------

1.everParod1 | 1.099848 .0331982 3.15 0.002 1.036668 1.166878

|

cohort |

30-39 | 1.200659 .0597628 3.67 0.000 1.089058 1.323696

40-49 | 1.528273 .0697781 9.29 0.000 1.397451 1.671341

50-59 | 1.908756 .0887975 13.90 0.000 1.742415 2.090977

60-69 | 2.595247 .1232806 20.08 0.000 2.364528 2.848478

≥70 | 3.62339 .2081824 22.41 0.000 3.237496 4.055282

|

Kon |

Female | .9513001 .0271543 -1.75 0.080 .8995401 1.006038

|

Education |

Up to Lower secondary education | 1.195327 .0459783 4.64 0.000 1.108524 1.288926

Post-secondary ≥2 years to Tertiary education | .7496298 .0257259 -8.40 0.000 .7008663 .8017861

|

No_5p_rank | 1.045097 .0065456 7.04 0.000 1.032346 1.058005

----------------------------------------------------------------------------------------------------------------

### Cox regression model T1D with and without periodontitis (outcome: ischemic heart disease)

Cox regression with Breslow method for ties

No. of subjects = 27,290 Number of obs = 27,290

No. of failures = 1,982

Time at risk = 243,289

LR chi2(10) = 2573.82

Log likelihood = -18755.837 Prob > chi2 = 0.0000

----------------------------------------------------------------------------------------------------------------

_t | Haz. ratio Std. err. z P>|z| [95% conf. interval]

-----------------------------------------------+----------------------------------------------------------------

1.everParod1 | .9508762 .0442041 -1.08 0.279 .8680676 1.041584

|

cohort |

30-39 | 5.800826 1.393203 7.32 0.000 3.62288 9.288075

40-49 | 20.84 4.591744 13.78 0.000 13.53162 32.09561

50-59 | 46.31931 10.08966 17.61 0.000 30.2236 70.98685

60-69 | 69.34966 15.11117 19.45 0.000 45.24484 106.2967

≥70 | 114.8771 25.37307 21.48 0.000 74.51219 177.1086

|

Kon |

Female | .8264167 .0380096 -4.15 0.000 .7551784 .9043751

|

Education |

Up to Lower secondary education | 1.18656 .0654212 3.10 0.002 1.065022 1.321968

Post-secondary ≥2 years to Tertiary education | .7920238 .0468965 -3.94 0.000 .7052413 .8894852

|

No_5p_rank | 1.02836 .0109159 2.63 0.008 1.007187 1.049979

----------------------------------------------------------------------------------------------------------------

### Cox regression model T1D with and without periodontitis (outcome: stroke)

Cox regression with Breslow method for ties

No. of subjects = 27,938 Number of obs = 27,938

No. of failures = 873

Time at risk = 253,965

LR chi2(10) = 968.80

Log likelihood = -8365.8013 Prob > chi2 = 0.0000

----------------------------------------------------------------------------------------------------------------

_t | Haz. ratio Std. err. z P>|z| [95% conf. interval]

-----------------------------------------------+----------------------------------------------------------------

1.everParod1 | 1.039454 .0726304 0.55 0.580 .9064182 1.192015

|

cohort |

30-39 | 4.369814 1.110932 5.80 0.000 2.654991 7.192216

40-49 | 9.547933 2.226244 9.68 0.000 6.045594 15.07925

50-59 | 15.50635 3.57449 11.89 0.000 9.86944 24.36277

60-69 | 24.93709 5.724279 14.01 0.000 15.90207 39.10552

≥70 | 57.74206 13.39809 17.48 0.000 36.6425 90.99121

|

Kon |

Female | .7736698 .0540637 -3.67 0.000 .6746431 .887232

|

Education |

Up to Lower secondary education | 1.070732 .0894788 0.82 0.413 .9089665 1.261287

Post-secondary ≥2 years to Tertiary education | .7191097 .0654345 -3.62 0.000 .6016461 .8595065

|

No_5p_rank | 1.060272 .0144739 4.29 0.000 1.032279 1.089023

----------------------------------------------------------------------------------------------------------------

### Cox regression model T1D with and without periodontitis (outcome: death)

Cox regression with Breslow method for ties

No. of subjects = 28,041 Number of obs = 28,041

No. of failures = 1,942

Time at risk = 257,631

LR chi2(10) = 3478.28

Log likelihood = -17952.2 Prob > chi2 = 0.0000

----------------------------------------------------------------------------------------------------------------

_t | Haz. ratio Std. err. z P>|z| [95% conf. interval]

-----------------------------------------------+----------------------------------------------------------------

1.everParod1 | .7744165 .0372397 -5.32 0.000 .704762 .8509552

|

cohort |

30-39 | 1.624549 .281958 2.80 0.005 1.156106 2.282801

40-49 | 3.653772 .5284121 8.96 0.000 2.751946 4.851132

50-59 | 7.916757 1.082656 15.13 0.000 6.055378 10.35031

60-69 | 18.63869 2.466815 22.10 0.000 14.38003 24.15857

≥70 | 59.69864 7.872017 31.01 0.000 46.10232 77.30473

|

Kon |

Female | .7937009 .0370559 -4.95 0.000 .7242965 .8697558

|

Education |

Up to Lower secondary education | 1.289715 .0670992 4.89 0.000 1.164686 1.428166

Post-secondary ≥2 years to Tertiary education | .5791211 .0396644 -7.98 0.000 .5063723 .6623214

|

No_5p_rank | 1.023645 .0105113 2.28 0.023 1.003249 1.044455

----------------------------------------------------------------------------------------------------------------

### Cox regression model T2D with and without periodontitis (outcome: retinopathy)

Cox regression with Breslow method for ties

No. of subjects = 187,388 Number of obs = 187,388

No. of failures = 63,032

Time at risk = 1,100,711

LR chi2(10) = 454.63

Log likelihood = -739350.52 Prob > chi2 = 0.0000

----------------------------------------------------------------------------------------------------------------

_t | Haz. ratio Std. err. z P>|z| [95% conf. interval]

-----------------------------------------------+----------------------------------------------------------------

1.everParod1 | 1.077622 .0086716 9.29 0.000 1.060759 1.094752

|

cohort |

30-39 | 1.118431 .0505392 2.48 0.013 1.023636 1.222005

40-49 | 1.119879 .0466907 2.72 0.007 1.032006 1.215234

50-59 | 1.102734 .0451976 2.39 0.017 1.017613 1.194975

60-69 | 1.130943 .0460797 3.02 0.003 1.044141 1.224962

≥70 | 1.123552 .0460985 2.84 0.005 1.036738 1.217636

|

Kon |

Female | .881117 .0071929 -15.50 0.000 .8671313 .8953283

|

Education |

Up to Lower secondary education | 1.071312 .0097544 7.57 0.000 1.052363 1.090602

Post-secondary ≥2 years to Tertiary education | .987202 .0111481 -1.14 0.254 .9655921 1.009296

|

No_5p_rank | 1.005197 .0017968 2.90 0.004 1.001681 1.008725

----------------------------------------------------------------------------------------------------------------

### Cox regression model T2D with and without periodontitis (outcome: albuminuria)

Cox regression with Breslow method for ties

No. of subjects = 190,677 Number of obs = 190,677

No. of failures = 62,289

Time at risk = 1,133,619

LR chi2(10) = 2866.23

Log likelihood = -730149.66 Prob > chi2 = 0.0000

----------------------------------------------------------------------------------------------------------------

_t | Haz. ratio Std. err. z P>|z| [95% conf. interval]

-----------------------------------------------+----------------------------------------------------------------

1.everParod1 | 1.063475 .008608 7.60 0.000 1.046737 1.080481

|

cohort |

30-39 | .9473513 .0447314 -1.15 0.252 .8636138 1.039208

40-49 | .956334 .0411102 -1.04 0.299 .8790606 1.0404

50-59 | 1.015592 .0427257 0.37 0.713 .9352101 1.102882

60-69 | 1.176229 .0490838 3.89 0.000 1.083855 1.276475

≥70 | 1.502256 .062882 9.72 0.000 1.383929 1.630699

|

Kon |

Female | .7728591 .0063967 -31.13 0.000 .7604229 .7854986

|

Education |

Up to Lower secondary education | 1.05802 .0095958 6.22 0.000 1.039379 1.076995

Post-secondary ≥2 years to Tertiary education | .9247508 .0107353 -6.74 0.000 .9039475 .9460327

|

No_5p_rank | 1.012124 .0017725 6.88 0.000 1.008655 1.015604

----------------------------------------------------------------------------------------------------------------

### Cox regression model T2D with and without periodontitis (outcome: ischemic heart disease)

Cox regression with Breslow method for ties

No. of subjects = 214,426 Number of obs = 214,426

No. of failures = 29,867

Time at risk = 1,430,544

LR chi2(10) = 11346.77

Log likelihood = -351074.26 Prob > chi2 = 0.0000

----------------------------------------------------------------------------------------------------------------

_t | Haz. ratio Std. err. z P>|z| [95% conf. interval]

-----------------------------------------------+----------------------------------------------------------------

1.everParod1 | .933064 .0109432 -5.91 0.000 .9118604 .9547607

|

cohort |

30-39 | 2.819661 .6654262 4.39 0.000 1.775487 4.47792

40-49 | 6.579414 1.482194 8.36 0.000 4.23088 10.2316

50-59 | 12.21048 2.736781 11.16 0.000 7.869551 18.94593

60-69 | 19.12648 4.282106 13.18 0.000 12.33289 29.66232

≥70 | 34.99851 7.834611 15.88 0.000 22.56854 54.27449

|

Kon |

Female | .5652654 .0069854 -46.16 0.000 .5517388 .5791236

|

Education |

Up to Lower secondary education | 1.110296 .0141886 8.19 0.000 1.082832 1.138456

Post-secondary ≥2 years to Tertiary education | .896951 .0158898 -6.14 0.000 .8663421 .9286413

|

No_5p_rank | 1.023575 .0025446 9.37 0.000 1.0186 1.028575

----------------------------------------------------------------------------------------------------------------

### Cox regression model T2D with and without periodontitis (outcome: stroke)

Cox regression with Breslow method for ties

No. of subjects = 228,866 Number of obs = 228,866

No. of failures = 13,561

Time at risk = 1,597,660

LR chi2(10) = 5853.82

Log likelihood = -159635.7 Prob > chi2 = 0.0000

----------------------------------------------------------------------------------------------------------------

_t | Haz. ratio Std. err. z P>|z| [95% conf. interval]

-----------------------------------------------+----------------------------------------------------------------

1.everParod1 | .9466764 .0165093 -3.14 0.002 .9148654 .9795935

|

cohort |

30-39 | 1.614247 .488706 1.58 0.114 .8918109 2.921914

40-49 | 3.41469 .9597446 4.37 0.000 1.968383 5.923698

50-59 | 5.524441 1.53915 6.13 0.000 3.199907 9.537605

60-69 | 10.33701 2.872412 8.41 0.000 5.996065 17.82066

≥70 | 22.64222 6.288785 11.23 0.000 13.13715 39.02445

|

Kon |

Female | .7823883 .0138862 -13.83 0.000 .7556397 .8100837

|

Education |

Up to Lower secondary education | 1.069446 .0202128 3.55 0.000 1.030555 1.109806

Post-secondary ≥2 years to Tertiary education | .9180313 .0242636 -3.24 0.001 .8716863 .9668404

|

No_5p_rank | 1.014328 .0037698 3.83 0.000 1.006966 1.021743

----------------------------------------------------------------------------------------------------------------

### Cox regression model T2D with and without periodontitis (outcome: death)

Cox regression with Breslow method for ties

No. of subjects = 233,478 Number of obs = 233,478

No. of failures = 34,743

Time at risk = 1,661,307

LR chi2(10) = 29255.86

Log likelihood = -400399.56 Prob > chi2 = 0.0000

----------------------------------------------------------------------------------------------------------------

_t | Haz. ratio Std. err. z P>|z| [95% conf. interval]

-----------------------------------------------+----------------------------------------------------------------

1.everParod1 | .7711431 .0085884 -23.33 0.000 .7544926 .7881611

|

cohort |

30-39 | 1.251517 .2540876 1.11 0.269 .8406647 1.863163

40-49 | 1.733877 .3242397 2.94 0.003 1.201825 2.50147

50-59 | 3.529571 .6484719 6.86 0.000 2.462264 5.059519

60-69 | 7.158449 1.309903 10.76 0.000 5.001037 10.24655

≥70 | 26.50508 4.844722 17.93 0.000 18.52433 37.92414

|

Kon |

Female | .8044119 .0088723 -19.73 0.000 .7872091 .8219907

|

Education |

Up to Lower secondary education | 1.216247 .0141676 16.81 0.000 1.188793 1.244334

Post-secondary ≥2 years to Tertiary education | .8250379 .0148608 -10.68 0.000 .7964195 .8546846

|

No_5p_rank | .9938541 .0024718 -2.48 0.013 .9890212 .9987106

**----------------------------------------------------------------------------------------------------------------**

## Number of teeth with PPD ≥6 mm

### Cox regression model T1D with and without periodontitis (outcome: retinopathy)

Cox regression with Breslow method for ties

No. of subjects = 17,788 Number of obs = 17,788

No. of failures = 12,239

Time at risk = 88,930

LR chi2(10) = 96.23

Log likelihood = -113559.15 Prob > chi2 = 0.0000

----------------------------------------------------------------------------------------------------------------

_t | Haz. ratio Std. err. z P>|z| [95% conf. interval]

-----------------------------------------------+----------------------------------------------------------------

perio_numberTeeth | 1.013054 .0035551 3.70 0.000 1.00611 1.020046

|

cohort |

30-39 | 1.117955 .0295451 4.22 0.000 1.061522 1.177388

40-49 | 1.134646 .0298877 4.80 0.000 1.077553 1.194763

50-59 | 1.14478 .0335197 4.62 0.000 1.080932 1.212399

60-69 | 1.179904 .0387936 5.03 0.000 1.106268 1.258441

≥70 | 1.198902 .0567402 3.83 0.000 1.092695 1.315432

|

Kon |

Female | .9903381 .0181772 -0.53 0.597 .9553446 1.026613

|

Education |

Up to Lower secondary education | .9802985 .0285926 -0.68 0.495 .9258298 1.037972

Post-secondary ≥2 years to Tertiary education | .9411897 .0189899 -3.00 0.003 .9046965 .9791549

|

No_5p_rank | 1.012635 .0047094 2.70 0.007 1.003446 1.021907

----------------------------------------------------------------------------------------------------------------

### Cox regression model T1D with and without periodontitis (outcome: albuminuria)

Cox regression with Breslow method for ties

No. of subjects = 24,817 Number of obs = 24,817

No. of failures = 5,083

Time at risk = 204,357

LR chi2(10) = 1179.02

Log likelihood = -50049.654 Prob > chi2 = 0.0000

----------------------------------------------------------------------------------------------------------------

_t | Haz. ratio Std. err. z P>|z| [95% conf. interval]

-----------------------------------------------+----------------------------------------------------------------

perio_numberTeeth | 1.022029 .0049772 4.47 0.000 1.012321 1.031831

|

cohort |

30-39 | 1.200549 .0597363 3.67 0.000 1.088996 1.323529

40-49 | 1.52581 .0695885 9.26 0.000 1.395337 1.668482

50-59 | 1.905609 .0883109 13.91 0.000 1.74015 2.086799

60-69 | 2.606415 .1224479 20.39 0.000 2.377139 2.857805

≥70 | 3.650006 .2086192 22.65 0.000 3.26319 4.082674

|

Kon |

Female | .9523241 .0271806 -1.71 0.087 .9005138 1.007115

|

Education |

Up to Lower secondary education | 1.191329 .0458366 4.55 0.000 1.104794 1.284641

Post-secondary ≥2 years to Tertiary education | .7519518 .0258213 -8.30 0.000 .7030085 .8043025

|

No_5p_rank | 1.044719 .0065488 6.98 0.000 1.031962 1.057634

----------------------------------------------------------------------------------------------------------------

### Cox regression model T1D with and without periodontitis (outcome: ischemic heart disease)

Cox regression with Breslow method for ties

No. of subjects = 27,290 Number of obs = 27,290

No. of failures = 1,982

Time at risk = 243,289

LR chi2(10) = 2573.28

Log likelihood = -18756.105 Prob > chi2 = 0.0000

----------------------------------------------------------------------------------------------------------------

_t | Haz. ratio Std. err. z P>|z| [95% conf. interval]

-----------------------------------------------+----------------------------------------------------------------

perio_numberTeeth | .9935326 .0081208 -0.79 0.427 .9777429 1.009577

|

cohort |

30-39 | 5.790759 1.390718 7.31 0.000 3.616675 9.271745

40-49 | 20.78236 4.578399 13.77 0.000 13.49501 32.0049

50-59 | 46.10706 10.03943 17.59 0.000 30.09023 70.64957

60-69 | 68.86806 14.99245 19.44 0.000 44.94825 105.5171

≥70 | 114.1397 25.1928 21.46 0.000 74.05598 175.9191

|

Kon |

Female | .8262206 .0380355 -4.15 0.000 .7549367 .9042355

|

Education |

Up to Lower secondary education | 1.18659 .0654257 3.10 0.002 1.065044 1.322007

Post-secondary ≥2 years to Tertiary education | .7921507 .046918 -3.93 0.000 .7053299 .8896585

|

No_5p_rank | 1.028457 .0109153 2.64 0.008 1.007284 1.050075

----------------------------------------------------------------------------------------------------------------

### Cox regression model T1D with and without periodontitis (outcome: stroke)

Cox regression with Breslow method for ties

No. of subjects = 27,938 Number of obs = 27,938

No. of failures = 873

Time at risk = 253,965

LR chi2(10) = 970.36

Log likelihood = -8365.0197 Prob > chi2 = 0.0000

----------------------------------------------------------------------------------------------------------------

_t | Haz. ratio Std. err. z P>|z| [95% conf. interval]

-----------------------------------------------+----------------------------------------------------------------

perio_numberTeeth | 1.016182 .011643 1.40 0.161 .9936166 1.03926

|

cohort |

30-39 | 4.359409 1.108178 5.79 0.000 2.648799 7.174742

40-49 | 9.494074 2.213135 9.66 0.000 6.012175 14.99248

50-59 | 15.38392 3.543405 11.87 0.000 9.79509 24.16161

60-69 | 24.76856 5.673947 14.01 0.000 15.80916 38.80546

≥70 | 57.61747 13.34707 17.50 0.000 36.59094 90.72663

|

Kon |

Female | .7767747 .0543381 -3.61 0.000 .6772524 .8909218

|

Education |

Up to Lower secondary education | 1.069294 .0893387 0.80 0.423 .9077785 1.259547

Post-secondary ≥2 years to Tertiary education | .7216985 .0657007 -3.58 0.000 .6037618 .8626726

|

No_5p_rank | 1.060114 .0144753 4.28 0.000 1.032119 1.088868

----------------------------------------------------------------------------------------------------------------

### Cox regression model T1D with and without periodontitis (outcome: death)

Cox regression with Breslow method for ties

No. of subjects = 28,041 Number of obs = 28,041

No. of failures = 1,942

Time at risk = 257,631

LR chi2(10) = 3452.82

Log likelihood = -17964.927 Prob > chi2 = 0.0000

----------------------------------------------------------------------------------------------------------------

_t | Haz. ratio Std. err. z P>|z| [95% conf. interval]

-----------------------------------------------+----------------------------------------------------------------

perio_numberTeeth | .9836942 .0088997 -1.82 0.069 .9664049 1.001293

|

cohort |

30-39 | 1.602791 .2781665 2.72 0.007 1.140643 2.252185

40-49 | 3.567883 .5159224 8.80 0.000 2.687357 4.736919

50-59 | 7.620365 1.041572 14.86 0.000 5.829498 9.961399

60-69 | 17.70468 2.338839 21.75 0.000 13.66602 22.93688

≥70 | 57.21464 7.531548 30.74 0.000 44.20362 74.05537

|

Kon |

Female | .7941407 .037119 -4.93 0.000 .7246218 .8703291

|

Education |

Up to Lower secondary education | 1.290631 .0670965 4.91 0.000 1.165602 1.429071

Post-secondary ≥2 years to Tertiary education | .5812027 .0398194 -7.92 0.000 .5081712 .6647299

|

No_5p_rank | 1.024305 .0105135 2.34 0.019 1.003905 1.04512

----------------------------------------------------------------------------------------------------------------

### Cox regression model T2D with and without periodontitis (outcome: retinopathy)

Cox regression with Breslow method for ties

No. of subjects = 187,388 Number of obs = 187,388

No. of failures = 63,032

Time at risk = 1,100,711

LR chi2(10) = 445.34

Log likelihood = -739355.17 Prob > chi2 = 0.0000

----------------------------------------------------------------------------------------------------------------

_t | Haz. ratio Std. err. z P>|z| [95% conf. interval]

-----------------------------------------------+----------------------------------------------------------------

perio_numberTeeth | 1.010753 .0012132 8.91 0.000 1.008378 1.013133

|

cohort |

30-39 | 1.118563 .0505462 2.48 0.013 1.023755 1.222151

40-49 | 1.121759 .0467656 2.76 0.006 1.033745 1.217267

50-59 | 1.108062 .0453983 2.50 0.012 1.022562 1.200711

60-69 | 1.140419 .0464305 3.23 0.001 1.052953 1.23515

≥70 | 1.133936 .0464989 3.07 0.002 1.046366 1.228835

|

Kon |

Female | .8818343 .0072081 -15.38 0.000 .8678192 .8960758

|

Education |

Up to Lower secondary education | 1.070588 .0097495 7.49 0.000 1.051649 1.089868

Post-secondary ≥2 years to Tertiary education | .9870097 .0111459 -1.16 0.247 .9654042 1.009099

|

No_5p_rank | 1.005033 .0017969 2.81 0.005 1.001517 1.008561

----------------------------------------------------------------------------------------------------------------

### Cox regression model T2D with and without periodontitis (outcome: albuminuria)

Cox regression with Breslow method for ties

No. of subjects = 190,677 Number of obs = 190,677

No. of failures = 62,289

Time at risk = 1,133,619

LR chi2(10) = 2929.32

Log likelihood = -730118.12 Prob > chi2 = 0.0000

----------------------------------------------------------------------------------------------------------------

_t | Haz. ratio Std. err. z P>|z| [95% conf. interval]

-----------------------------------------------+----------------------------------------------------------------

perio_numberTeeth | 1.013774 .0012358 11.22 0.000 1.011355 1.016199

|

cohort |

30-39 | .9438327 .0445672 -1.22 0.221 .8604028 1.035352

40-49 | .9537114 .0409939 -1.10 0.270 .8766562 1.037539

50-59 | 1.01491 .0426801 0.35 0.725 .9346134 1.102106

60-69 | 1.179912 .0491987 3.97 0.000 1.087319 1.280389

≥70 | 1.511186 .0632176 9.87 0.000 1.392225 1.640311

|

Kon |

Female | .775244 .0064238 -30.72 0.000 .7627553 .7879372

|

Education |

Up to Lower secondary education | 1.056859 .009585 6.10 0.000 1.038239 1.075814

Post-secondary ≥2 years to Tertiary education | .9251649 .0107399 -6.70 0.000 .9043527 .9464561

|

No_5p_rank | 1.011912 .0017727 6.76 0.000 1.008444 1.015393

----------------------------------------------------------------------------------------------------------------

### Cox regression model T2D with and without periodontitis (outcome: ischemic heart disease)

Cox regression with Breslow method for ties

No. of subjects = 214,426 Number of obs = 214,426

No. of failures = 29,867

Time at risk = 1,430,544

LR chi2(10) = 11313.80

Log likelihood = -351090.75 Prob > chi2 = 0.0000

----------------------------------------------------------------------------------------------------------------

_t | Haz. ratio Std. err. z P>|z| [95% conf. interval]

-----------------------------------------------+----------------------------------------------------------------

perio_numberTeeth | .9972616 .0019334 -1.41 0.157 .9934793 1.001058

|

cohort |

30-39 | 2.804704 .6618986 4.37 0.000 1.766066 4.454174

40-49 | 6.523594 1.469616 8.32 0.000 4.194987 10.14479

50-59 | 12.06568 2.704273 11.11 0.000 7.776293 18.72109

60-69 | 18.85524 4.221187 13.12 0.000 12.15823 29.24108

≥70 | 34.58766 7.742333 15.83 0.000 22.30399 53.63643

|

Kon |

Female | .5671999 .0070172 -45.83 0.000 .5536119 .5811214

|

Education |

Up to Lower secondary education | 1.11077 .0141924 8.22 0.000 1.083299 1.138938

Post-secondary ≥2 years to Tertiary education | .8976175 .0159019 -6.10 0.000 .8669852 .9293322

|

No_5p_rank | 1.023651 .0025445 9.40 0.000 1.018676 1.02865

----------------------------------------------------------------------------------------------------------------

### Cox regression model T2D with and without periodontitis (outcome: stroke)

Cox regression with Breslow method for ties

No. of subjects = 228,866 Number of obs = 228,866

No. of failures = 13,561

Time at risk = 1,597,660

LR chi2(10) = 5845.19

Log likelihood = -159640.01 Prob > chi2 = 0.0000

----------------------------------------------------------------------------------------------------------------

_t | Haz. ratio Std. err. z P>|z| [95% conf. interval]

-----------------------------------------------+----------------------------------------------------------------

perio_numberTeeth | 1.0033 .0029277 1.13 0.259 .9975784 1.009055

|

cohort |

30-39 | 1.601217 .4847648 1.55 0.120 .8846081 2.898342

40-49 | 3.374163 .9483577 4.33 0.000 1.945017 5.853406

50-59 | 5.441543 1.516019 6.08 0.000 3.15193 9.394369

60-69 | 10.17227 2.826458 8.35 0.000 5.900699 17.53606

≥70 | 22.38004 6.215626 11.19 0.000 12.98542 38.57142

|

Kon |

Female | .7868412 .0139859 -13.49 0.000 .7599013 .8147361

|

Education |

Up to Lower secondary education | 1.06998 .0202184 3.58 0.000 1.031077 1.11035

Post-secondary ≥2 years to Tertiary education | .919094 .0242919 -3.19 0.001 .8726948 .9679601

|

No_5p_rank | 1.014322 .0037696 3.83 0.000 1.006961 1.021738

----------------------------------------------------------------------------------------------------------------

### Cox regression model T2D with and without periodontitis (outcome: death)

Cox regression with Breslow method for ties

No. of subjects = 233,478 Number of obs = 233,478

No. of failures = 34,743

Time at risk = 1,661,307

LR chi2(10) = 28917.92

Log likelihood = -400568.53 Prob > chi2 = 0.0000

----------------------------------------------------------------------------------------------------------------

_t | Haz. ratio Std. err. z P>|z| [95% conf. interval]

-----------------------------------------------+----------------------------------------------------------------

perio_numberTeeth | .969708 .0021067 -14.16 0.000 .9655878 .9738458

|

cohort |

30-39 | 1.243774 .2525168 1.07 0.283 .8354619 1.851639

40-49 | 1.710792 .319924 2.87 0.004 1.185822 2.468168

50-59 | 3.448823 .633629 6.74 0.000 2.405943 4.943749

60-69 | 6.929528 1.267967 10.58 0.000 4.841173 9.918744

≥70 | 25.68848 4.695292 17.76 0.000 17.95384 36.75525

|

Kon |

Female | .8076779 .0089182 -19.34 0.000 .7903863 .8253478

|

Education |

Up to Lower secondary education | 1.219443 .0142017 17.04 0.000 1.191923 1.247598

Post-secondary ≥2 years to Tertiary education | .8255735 .0148705 -10.64 0.000 .7969362 .8552398

|

No_5p_rank | .9942871 .0024717 -2.30 0.021 .9894546 .9991433

----------------------------------------------------------------------------------------------------------------

## Number of teeth

### Cox regression model T1D with and without periodontitis (outcome: retinopathy)

Cox regression with Breslow method for ties

No. of subjects = 18,035 Number of obs = 18,035

No. of failures = 12,414

Time at risk = 90,129

LR chi2(10) = 85.33

Log likelihood = -115359.55 Prob > chi2 = 0.0000

----------------------------------------------------------------------------------------------------------------

_t | Haz. ratio Std. err. z P>|z| [95% conf. interval]

-----------------------------------------------+----------------------------------------------------------------

maximum_AT | 1.000448 .0016928 0.26 0.791 .9971355 1.003771

|

cohort |

30-39 | 1.123103 .0294676 4.42 0.000 1.066807 1.182369

40-49 | 1.142784 .0298067 5.12 0.000 1.085832 1.202723

50-59 | 1.156058 .0336376 4.98 0.000 1.091974 1.223902

60-69 | 1.203416 .0396272 5.62 0.000 1.128202 1.283645

≥70 | 1.212586 .0583872 4.00 0.000 1.103383 1.332596

|

Kon |

Female | .9874234 .0179921 -0.69 0.487 .9527818 1.023324

|

Education |

Up to Lower secondary education | .9815353 .0285025 -0.64 0.521 .9272315 1.039019

Post-secondary ≥2 years to Tertiary education | .9349322 .0187431 -3.36 0.001 .8989088 .9723992

|

No_5p_rank | 1.012638 .004662 2.73 0.006 1.003542 1.021817

----------------------------------------------------------------------------------------------------------------

### Cox regression model T1D with and without periodontitis (outcome: albuminuria)

Cox regression with Breslow method for ties

No. of subjects = 25,190 Number of obs = 25,190

No. of failures = 5,168

Time at risk = 207,422

LR chi2(10) = 1206.81

Log likelihood = -50959.636 Prob > chi2 = 0.0000

----------------------------------------------------------------------------------------------------------------

_t | Haz. ratio Std. err. z P>|z| [95% conf. interval]

-----------------------------------------------+----------------------------------------------------------------

maximum_AT | .9860528 .0021266 -6.51 0.000 .9818935 .9902297

|

cohort |

30-39 | 1.204934 .0594879 3.78 0.000 1.093803 1.327356

40-49 | 1.522843 .0688805 9.30 0.000 1.393651 1.664011

50-59 | 1.889293 .08682 13.84 0.000 1.726567 2.067356

60-69 | 2.509461 .1186498 19.46 0.000 2.287362 2.753126

≥70 | 3.354264 .1975277 20.55 0.000 2.988624 3.764638

|

Kon |

Female | .934621 .0264624 -2.39 0.017 .8841685 .9879524

|

Education |

Up to Lower secondary education | 1.17494 .0449728 4.21 0.000 1.09002 1.266476

Post-secondary ≥2 years to Tertiary education | .753338 .0256921 -8.31 0.000 .7046285 .8054146

|

No_5p_rank | 1.042107 .0064912 6.62 0.000 1.029462 1.054907

----------------------------------------------------------------------------------------------------------------

### Cox regression model T1D with and without periodontitis (outcome: ischemic heart disease)

Cox regression with Breslow method for ties

No. of subjects = 27,697 Number of obs = 27,697

No. of failures = 2,024

Time at risk = 246,890

LR chi2(10) = 2647.02

Log likelihood = -19173.856 Prob > chi2 = 0.0000

----------------------------------------------------------------------------------------------------------------

_t | Haz. ratio Std. err. z P>|z| [95% conf. interval]

-----------------------------------------------+----------------------------------------------------------------

maximum_AT | .9808004 .002974 -6.39 0.000 .9749888 .9866467

|

cohort |

30-39 | 5.796203 1.388515 7.34 0.000 3.624375 9.269451

40-49 | 20.40805 4.492557 13.70 0.000 13.25626 31.41825

50-59 | 44.57033 9.699038 17.45 0.000 29.09472 68.27749

60-69 | 63.85931 13.911 19.08 0.000 41.66774 97.86974

≥70 | 99.92518 22.15475 20.77 0.000 64.70713 154.3113

|

Kon |

Female | .8295041 .037724 -4.11 0.000 .7587659 .9068372

|

Education |

Up to Lower secondary education | 1.143256 .0627595 2.44 0.015 1.026636 1.273124

Post-secondary ≥2 years to Tertiary education | .8076512 .0473533 -3.64 0.000 .7199746 .9060049

|

No_5p_rank | 1.025776 .0108083 2.42 0.016 1.00481 1.04718

----------------------------------------------------------------------------------------------------------------

### Cox regression model T1D with and without periodontitis (outcome: stroke)

Cox regression with Breslow method for ties

No. of subjects = 28,354 Number of obs = 28,354

No. of failures = 887

Time at risk = 257,784

LR chi2(10) = 1000.82

Log likelihood = -8505.135 Prob > chi2 = 0.0000

----------------------------------------------------------------------------------------------------------------

_t | Haz. ratio Std. err. z P>|z| [95% conf. interval]

-----------------------------------------------+----------------------------------------------------------------

maximum_AT | .9802701 .0044536 -4.39 0.000 .9715799 .989038

|

cohort |

30-39 | 4.289747 1.090463 5.73 0.000 2.60648 7.060071

40-49 | 9.396143 2.187399 9.62 0.000 5.953768 14.82884

50-59 | 15.06378 3.465448 11.79 0.000 9.596517 23.64582

60-69 | 23.18563 5.320482 13.70 0.000 14.78737 36.35356

≥70 | 50.58813 11.8318 16.78 0.000 31.9864 80.0077

|

Kon |

Female | .765518 .0530869 -3.85 0.000 .6682308 .8769691

|

Education |

Up to Lower secondary education | 1.047542 .0871855 0.56 0.577 .8898709 1.233149

Post-secondary ≥2 years to Tertiary education | .7251791 .0657847 -3.54 0.000 .6070556 .8662875

|

No_5p_rank | 1.055459 .0144349 3.95 0.000 1.027543 1.084133

----------------------------------------------------------------------------------------------------------------

### Cox regression model T1D with and without periodontitis (outcome: death)

Cox regression with Breslow method for ties

No. of subjects = 28,459 Number of obs = 28,459

No. of failures = 1,958

Time at risk = 261,529

LR chi2(10) = 3582.47

Log likelihood = -18091.619 Prob > chi2 = 0.0000

----------------------------------------------------------------------------------------------------------------

_t | Haz. ratio Std. err. z P>|z| [95% conf. interval]

-----------------------------------------------+----------------------------------------------------------------

maximum_AT | .9692833 .0027148 -11.14 0.000 .9639769 .9746188

|

cohort |

30-39 | 1.589974 .2740163 2.69 0.007 1.134211 2.228877

40-49 | 3.345542 .4834353 8.36 0.000 2.520385 4.440852

50-59 | 6.972799 .9520126 14.22 0.000 5.33569 9.112209

60-69 | 15.1103 2.00172 20.50 0.000 11.65497 19.59003

≥70 | 45.21701 6.02971 28.58 0.000 34.81719 58.72323

|

Kon |

Female | .7856174 .0365653 -5.18 0.000 .7171224 .8606546

|

Education |

Up to Lower secondary education | 1.208235 .0630475 3.63 0.000 1.090774 1.338346

Post-secondary ≥2 years to Tertiary education | .6015012 .0411528 -7.43 0.000 .5260172 .6878172

|

No_5p_rank | 1.022077 .0103816 2.15 0.032 1.001931 1.042628

----------------------------------------------------------------------------------------------------------------

### Cox regression model T2D with and without periodontitis (outcome: retinopathy)

Cox regression with Breslow method for ties

No. of subjects = 191,425 Number of obs = 191,425

No. of failures = 64,261

Time at risk = 1,125,121

LR chi2(10) = 397.45

Log likelihood = -755175.07 Prob > chi2 = 0.0000

----------------------------------------------------------------------------------------------------------------

_t | Haz. ratio Std. err. z P>|z| [95% conf. interval]

-----------------------------------------------+----------------------------------------------------------------

maximum_AT | .9972122 .0005452 -5.11 0.000 .9961442 .9982813

|

cohort |

30-39 | 1.125653 .0504856 2.64 0.008 1.030927 1.229082

40-49 | 1.127315 .0466921 2.89 0.004 1.039416 1.222647

50-59 | 1.114094 .0453643 2.65 0.008 1.028637 1.206651

60-69 | 1.141098 .0462346 3.26 0.001 1.053985 1.235412

≥70 | 1.119742 .0458535 2.76 0.006 1.033383 1.213318

|

Kon |

Female | .8765366 .0070724 -16.33 0.000 .862784 .8905085

|

Education |

Up to Lower secondary education | 1.06704 .0096693 7.16 0.000 1.048256 1.086161

Post-secondary ≥2 years to Tertiary education | .9895169 .0110861 -0.94 0.347 .9680254 1.011486

|

No_5p_rank | 1.004595 .001782 2.58 0.010 1.001108 1.008094

----------------------------------------------------------------------------------------------------------------

### Cox regression model T2D with and without periodontitis (outcome: albuminuria)

Cox regression with Breslow method for ties

No. of subjects = 194,636 Number of obs = 194,636

No. of failures = 63,478

Time at risk = 1,158,230

LR chi2(10) = 3114.40

Log likelihood = -745345.88 Prob > chi2 = 0.0000

----------------------------------------------------------------------------------------------------------------

_t | Haz. ratio Std. err. z P>|z| [95% conf. interval]

-----------------------------------------------+----------------------------------------------------------------

maximum_AT | .9911666 .0005274 -16.67 0.000 .9901335 .9922009

|

cohort |

30-39 | .9420343 .0441261 -1.27 0.202 .8594 1.032614

40-49 | .9504828 .0405735 -1.19 0.234 .874196 1.033427

50-59 | 1.004797 .0419791 0.11 0.909 .9257982 1.090537

60-69 | 1.143982 .0474565 3.24 0.001 1.05465 1.240881

≥70 | 1.4212 .0593456 8.42 0.000 1.309518 1.542408

|

Kon |

Female | .7673043 .0062771 -32.38 0.000 .7550994 .7797065

|

Education |

Up to Lower secondary education | 1.046385 .0094474 5.02 0.000 1.028031 1.065066

Post-secondary ≥2 years to Tertiary education | .9372047 .0107971 -5.63 0.000 .91628 .9586072

|

No_5p_rank | 1.010607 .0017592 6.06 0.000 1.007165 1.014061

----------------------------------------------------------------------------------------------------------------

### Cox regression model T2D with and without periodontitis (outcome: ischemic heart disease)

Cox regression with Breslow method for ties

No. of subjects = 218,894 Number of obs = 218,894

No. of failures = 30,500

Time at risk = 1,460,702

LR chi2(10) = 12051.17

Log likelihood = -358918.02 Prob > chi2 = 0.0000

----------------------------------------------------------------------------------------------------------------

_t | Haz. ratio Std. err. z P>|z| [95% conf. interval]

-----------------------------------------------+----------------------------------------------------------------

maximum_AT | .9830805 .0007098 -23.63 0.000 .9816903 .9844727

|

cohort |

30-39 | 2.7891 .6572857 4.35 0.000 1.757389 4.426497

40-49 | 6.453572 1.453535 8.28 0.000 4.150348 10.03496

50-59 | 11.65989 2.613203 10.96 0.000 7.51491 18.09109

60-69 | 17.68353 3.959134 12.83 0.000 11.40237 27.42477

≥70 | 30.8158 6.900156 15.31 0.000 19.86898 47.79376

|

Kon |

Female | .5621629 .0068627 -47.18 0.000 .5488719 .5757757

|

Education |

Up to Lower secondary education | 1.076098 .013682 5.77 0.000 1.049613 1.103251

Post-secondary ≥2 years to Tertiary education | .9254976 .0162466 -4.41 0.000 .8941964 .9578945

|

No_5p_rank | 1.020962 .0025255 8.39 0.000 1.016024 1.025924

----------------------------------------------------------------------------------------------------------------

### Cox regression model T2D with and without periodontitis (outcome: stroke)

Cox regression with Breslow method for ties

No. of subjects = 233,625 Number of obs = 233,625

No. of failures = 13,821

Time at risk = 1,631,469

LR chi2(10) = 6123.16

Log likelihood = -162907.98 Prob > chi2 = 0.0000

----------------------------------------------------------------------------------------------------------------

_t | Haz. ratio Std. err. z P>|z| [95% conf. interval]

-----------------------------------------------+----------------------------------------------------------------

maximum_AT | .9846575 .0010494 -14.51 0.000 .9826029 .9867165

|

cohort |

30-39 | 1.389533 .395415 1.16 0.248 .7955087 2.427127

40-49 | 2.920228 .765288 4.09 0.000 1.747218 4.880749

50-59 | 4.594836 1.192433 5.88 0.000 2.762929 7.641353

60-69 | 8.328333 2.15519 8.19 0.000 5.015172 13.83026

≥70 | 17.49221 4.525999 11.06 0.000 10.5342 29.04609

|

Kon |

Female | .7815262 .0137054 -14.06 0.000 .7551205 .8088553

|

Education |

Up to Lower secondary education | 1.042235 .0196104 2.20 0.028 1.004499 1.081388

Post-secondary ≥2 years to Tertiary education | .944835 .0248071 -2.16 0.031 .8974437 .9947288

|

No_5p_rank | 1.012082 .003742 3.25 0.001 1.004775 1.019443

----------------------------------------------------------------------------------------------------------------

### Cox regression model T2D with and without periodontitis (outcome: death)

Cox regression with Breslow method for ties

No. of subjects = 238,326 Number of obs = 238,326

No. of failures = 35,217

Time at risk = 1,696,385

LR chi2(10) = 30879.76

Log likelihood = -405980.73 Prob > chi2 = 0.0000

----------------------------------------------------------------------------------------------------------------

_t | Haz. ratio Std. err. z P>|z| [95% conf. interval]

-----------------------------------------------+----------------------------------------------------------------

maximum_AT | .9733441 .0006178 -42.57 0.000 .972134 .9745557

|

cohort |

30-39 | 1.09742 .2169289 0.47 0.638 .7449281 1.616707

40-49 | 1.509957 .2736432 2.27 0.023 1.058534 2.153894

50-59 | 2.905974 .5171075 5.99 0.000 2.050329 4.118698

60-69 | 5.56341 .985909 9.68 0.000 3.930945 7.873813

≥70 | 19.38888 3.433034 16.74 0.000 13.70368 27.43267

|

Kon |

Female | .8084089 .0088358 -19.46 0.000 .7912752 .8259135

|

Education |

Up to Lower secondary education | 1.166461 .0135591 13.25 0.000 1.140186 1.193342

Post-secondary ≥2 years to Tertiary education | .8767266 .015739 -7.33 0.000 .8464151 .9081237

|

No_5p_rank | .9915055 .0024545 -3.45 0.001 .9867064 .996328

----------------------------------------------------------------------------------------------------------------

# Diabetes and periodontitis

## Table A9. Prevalence of periodontitis by age category and gender (comparing T1D to no diabetes)

|  | | No Diabetes | Type 1 Diabetes | Crude RR  (95%CI) | Adjusted RRs*  (95%CI) |
| --- | --- | --- | --- | --- | --- |
| 18-29  years | Female | 236 / 7 097  (3.3%) | 183 / 3 585  (5.1%) | 1.54  (1.27, 1.85) | **1.48**  **(1.22, 1.79)** |
|  | Male | 422 / 8 755  (4.8%) | 232 / 4 424  (5.2%) | 1.09  (0.93, 1.27) | 1.08  (0.92, 1.26) |
|  | Total | 658 / 15 852  (4.2%) | 415 / 8 009  (5.2%) | 1.25  (1.11, 1.41) | **1.23**  **(1.09, 1.38)** |
| 30-39  years | Female | 245 / 4 426  (5.5%) | 181 / 2 281  (7.9%) | 1.43  (1.19, 1.73) | **1.41**  **(1.17, 1.69)** |
|  | Male | 505 / 5 518  (9.2%) | 284 / 2 779  (10.2%) | 1.12  (0.97, 1.28) | 1.11  (0.97, 1.28) |
|  | Total | 750 / 9 944  (7.5%) | 465 / 5 060  (9.2%) | 1.22  (1.09, 1.36) | **1.21**  **(1.08, 1.35)** |
| 40-49  years | Female | 451 / 4 896  (9.2%) | 314 / 2 478  (12.7%) | 1.38  (1.20, 1.58) | **1.35**  **(1.18, 1.55)** |
|  | Male | 761 / 6 141  (12.4%) | 476 / 3 167  (15.0%) | 1.21  (1.09, 1.35) | **1.20**  **(1.08, 1.34)** |
|  | Total | 1 212 / 11 037  (11.0%) | 790 / 5 645  (14.0%) | 1.27  (1.17, 1.39) | **1.26**  **(1.16, 1.37)** |
| 50-59  years | Female | 660 / 4 177  (15.8%) | 357 / 2 050  (17.4%) | 1.10  (0.98, 1.24) | 1.08  (0.96, 1.22) |
|  | Male | 906 / 4 892  (18.5%) | 523 / 2 503  (20.9%) | 1.13  (1.02, 1.24) | **1.12**  **(1.02, 1.24)** |
|  | Total | 1 566 / 9 069  (17.3%) | 880 / 4 553  (19.3%) | 1.12  (1.04, 1.21) | **1.10**  **(1.02, 1.19)** |
| 60-69  years | Female | 583 / 3 383  (17.2%) | 289 / 1 581  (18.3%) | 1.06  (0.93, 1.21) | 1.05  (0.92, 1.20) |
|  | Male | 914 / 4 023  (22.7%) | 468 / 1 924  (24.3%) | 1.07  (0.97, 1.18) | 1.07  (0.97, 1.18) |
|  | Total | 1 497 / 7 406  (20.2%) | 757 / 3 505  (21.6%) | 1.07  (0.99, 1.15) | 1.06  (0.98, 1.15) |
| ≥70  years | Female | 277 / 1 919  (14.4%) | 131 / 846  (15.5%) | 1.07  (0.89, 1.30) | 1.03  (0.85, 1.26) |
|  | Male | 373 / 1 795  (20.8%) | 137 / 764  (17.9%) | 0.86  (0.72, 1.03) | 0.86  (0.72, 1.03) |
|  | Total | 650 / 3 714  (17.5%) | 268 / 1 610  (16.6%) | 0.95  (0.84, 1.08) | 0.94  (0.82, 1.07) |
| Overall | Female | 2 452 / 25 898  (9.5%) | 1 455 / 12 821  (11.3%) | 1.20  (1.13, 1.27) | **1.19**  **(1.12, 1.27)** |
|  | Male | 3 881 / 31 124  (12.5%) | 2 120 / 15 561  (13.6%) | 1.09  (1.04, 1.15) | **1.10**  **(1.04, 1.15)** |
|  | Total | 6 333 / 57 022  (11.1%) | 3 575 / 28 382  (12.6%) | 1.13  (1.09, 1.18) | **1.13**  **(1.09, 1.18)** |
| Prevalence is presented as frequency / n (%)  *Adjusted RRs originate from logistic regression models, which included diabetes, age category (and its interaction with diabetes), gender, level of education and number of years in the 5^th^ lowest level of income.  Statistically significant adjusted RRs are highlighted in **bold** | | | | | |

## Figure A1. Prevalence of periodontitis (2010-2020) in subjects with T1D and matched controls without diabetes, females and males by age category.

## Logistic regression model T1D versus matched controls without diabetes (outcome: periodontitis)

Logistic regression Number of obs = 85,042

LR chi2(15) = 3660.30

Prob > chi2 = 0.0000

Log likelihood = -28693.942 Pseudo R2 = 0.0600

---------------------------------------------------------------------------------------------------------

everParod2 | Odds ratio Std. err. z P>|z| [95% conf. interval]

----------------------------------------+----------------------------------------------------------------

cohort |

18-29 | 1 (base)

30-39 | 1.906142 .1057874 11.62 0.000 1.709681 2.125178

40-49 | 2.751468 .1393275 19.99 0.000 2.491505 3.038556

50-59 | 4.556775 .2242699 30.81 0.000 4.13775 5.018235

60-69 | 5.342634 .2683126 33.37 0.000 4.841804 5.895269

≥70 | 4.247262 .2572959 23.87 0.000 3.771758 4.782711

|

Group |

No Diabetes | 1 (base)

Type 1 Diabetes | 1.239947 .0804209 3.32 0.001 1.091932 1.408026

|

cohort#Group |

30-39#Type 1 Diabetes | .995091 .0893285 -0.05 0.956 .8345477 1.186518

40-49#Type 1 Diabetes | 1.05057 .0855291 0.61 0.545 .8956262 1.232318

50-59#Type 1 Diabetes | .9109861 .0729593 -1.16 0.244 .7786468 1.065818

60-69#Type 1 Diabetes | .8698582 .071534 -1.70 0.090 .7403698 1.021994

≥70#Type 1 Diabetes | .7469267 .077117 -2.83 0.005 .6100916 .9144521

|

Kon |

Male | 1 (base)

Female | .7728429 .0173834 -11.46 0.000 .7395121 .807676

|

Education |

Up to Lower secondary education | 1.301469 .0382068 8.98 0.000 1.228699 1.378549

Upper secondary to Post-secondary ed.. | 1 (base)

Post-secondary ≥2 years to Tertiary .. | .6977537 .0189023 -13.28 0.000 .6616722 .7358027

|

No_5p_rank | 1.017834 .0057163 3.15 0.002 1.006691 1.029099

_cons | .0529482 .00226 -68.84 0.000 .048699 .0575682

---------------------------------------------------------------------------------------------------------

Note: _cons estimates baseline odds.

Goodness-of-fit test after logistic model

Variable: everParod2

Table collapsed on quantiles of estimated probabilities

+----------------------------------------------------------+

| Group | Prob | Obs_1 | Exp_1 | Obs_0 | Exp_0 | Total |

|-------+--------+-------+--------+-------+--------+-------|

| 1 | 0.0388 | 262 | 274.8 | 8270 | 8257.2 | 8532 |

| 2 | 0.0503 | 389 | 400.3 | 8402 | 8390.7 | 8791 |

| 3 | 0.0645 | 421 | 469.8 | 7877 | 7828.2 | 8298 |

| 4 | 0.0878 | 629 | 640.1 | 8129 | 8117.9 | 8758 |

| 5 | 0.1012 | 837 | 799.0 | 7583 | 7621.0 | 8420 |

|-------+--------+-------+--------+-------+--------+-------|

| 6 | 0.1272 | 1041 | 1030.1 | 7628 | 7638.9 | 8669 |

| 7 | 0.1572 | 1335 | 1253.5 | 7561 | 7642.5 | 8896 |

| 8 | 0.1794 | 1444 | 1352.6 | 6612 | 6703.4 | 8056 |

| 9 | 0.2172 | 1574 | 1608.3 | 6581 | 6546.7 | 8155 |

| 10 | 0.3215 | 1935 | 2038.4 | 6532 | 6428.6 | 8467 |

+----------------------------------------------------------+

Number of observations = 85,042

Number of groups = 10

Hosmer–Lemeshow chi2(8) = 30.06

Prob > chi2 = 0.0002

### Figure A2. Probability estimates for periodontitis and their contrasts (based on logistic regression, stratified by gender), females and males by age category.

## Table A10. Prevalence of periodontitis by age category and gender (comparing T2D to no diabetes)

|  | | No Diabetes | Type 2 Diabetes | Crude RR  (95%CI) | Adjusted RRs*  (95%CI) |
| --- | --- | --- | --- | --- | --- |
| 18-29  years | Female | 201 / 5 056  (4.0%) | 185 / 2 077  (8.9%) | 2.24  (1.85, 2.72) | **2.00**  **(1.65, 2.44)** |
|  | Male | 321 / 5 190  (6.2%) | 205 / 2 070  (9.9%) | 1.60  (1.35, 1.89) | **1.49**  **(1.25, 1.77)** |
|  | Total | 522 / 10 246  (5.1%) | 390 / 4 147  (9.4%) | 1.85  (1.63, 2.09) | **1.71**  **(1.50, 1.94)** |
| 30-39  years | Female | 818 / 12 162  (6.7%) | 790 / 5 131  (15.4%) | 2.29  ( 2.09, 2.51) | **2.06**  **(1.88, 2.26)** |
|  | Male | 1 523 / 15 527  (9.8%) | 1 140 / 6 162  (18.5%) | 1.89  (1.76, 2.02) | **1.82**  **(1.70, 1.96)** |
|  | Total | 2 341 / 27 689  (8.5%) | 1 930 / 11 293  (17.1%) | 2.02  (1.91, 2.14) | **1.92**  **(1.81, 2.03)** |
| 40-49  years | Female | 3 138 / 29 796  (10.5%) | 2 506 / 13 513  (18.5%) | 1.76  (1.68, 1.85) | **1.66**  **(1.58, 1.74)** |
|  | Male | 5 945 / 44 305  (13.4%) | 4 378 / 19 338  (22.6%) | 1.69  (1.63, 1.75) | **1.65**  **(1.60, 1.71)** |
|  | Total | 9 083 / 74 101  (12.3%) | 6 884 / 32 851  (21.0%) | 1.71  (1.66, 1.76) | **1.66**  **(1.61, 1.71)** |
| 50-59  years | Female | 7 351 / 48 542  (15.1%) | 4 859 / 22 844  (21.3%) | 1.40  (1.36, 1.45) | **1.34**  **(1.30, 1.38)** |
|  | Male | 13 903 / 73 468  (18.9%) | 8 992 / 33 939  (26.5%) | 1.40  (1.37, 1.43) | **1.38**  **(1.35, 1.41)** |
|  | Total | 21 254 / 122 010  (17.4%) | 13 851 / 56 783  (24.4%) | 1.40  (1.37, 1.43) | **1.37**  **(1.34, 1.40)** |
| 60-69  years | Female | 12 024 / 68 957  (17.4%) | 6 569 / 32 969  (19.9%) | 1.14  (1.11, 1.17) | **1.11**  **(1.08, 1.14)** |
|  | Male | 21 904 / 95 645  (22.9%) | 12 613 / 45 847  (27.5%) | 1.20  (1.18, 1.22) | **1.18**  **(1.16, 1.21)** |
|  | Total | 33 928 / 164 602  (20.6%) | 19 182 / 78 816  (24.3%) | 1.18  (1.16, 1.20) | **1.16**  **(1.14, 1.18)** |
| ≥70 years | Female | 9 420 / 66 445  (14.2%) | 4 718 / 31 786  (14.8%) | 1.05  (1.01, 1.08) | 1.03  (0.99, 1.06) |
|  | Male | 12 343 / 64 112  (19.3%) | 6 362 / 30 813  (20.6%) | 1.07  (1.04, 1.10) | **1.07**  **(1.04, 1.10)** |
|  | Total | 21 763 / 130 557  (16.7%) | 11 080 / 62 599  (17.7%) | 1.06  (1.04, 1.08) | **1.05**  **(1.03, 1.07)** |
| Overall | Female | 32 952 / 230 958  (14.3%) | 19 627 / 108 320  (18.1%) | 1.27  (1.25, 1.29) | **1.22**  **(1.20, 1.24)** |
|  | Male | 55 939 / 298 247  (18.8%) | 33 690 / 138 169  (24.4%) | 1.30  (1.28, 1.32) | **1.27**  **(1.26, 1.29)** |
|  | Total | 88 891 / 529 205  (16.8%) | 53 317 / 246 489  (21.6%) | 1.29  (1.28, 1.30) | **1.26**  **(1.24 ,1.27)** |
| Prevalence is presented as frequency / n (%)  *Adjusted RRs originate from logistic regression models, which included diabetes, age category (and its interaction with diabetes), gender, level of education and number of years in the 5^th^ lowest level of income. Statistically significant adjusted RRs are highlighted in **bold** | | | | | |

Figure A3. Prevalence of periodontitis (2010-2020) in T2D and matched controls without diabetes, females and males by age category.

## Logistic regression model T2D versus matched controls without diabetes (outcome: periodontitis)

Logistic regression Number of obs = 770,672

LR chi2(15) = 14615.94

Prob > chi2 = 0.0000

Log likelihood = -359642.23 Pseudo R2 = 0.0199

---------------------------------------------------------------------------------------------------------

everParod2 | Odds ratio Std. err. z P>|z| [95% conf. interval]

----------------------------------------+----------------------------------------------------------------

cohort |

18-29 | 1 (base)

30-39 | 1.694295 .0849703 10.51 0.000 1.535679 1.869293

40-49 | 2.46151 .1146873 19.33 0.000 2.246685 2.696876

50-59 | 3.689637 .1691672 28.47 0.000 3.372536 4.036553

60-69 | 4.523635 .2064842 33.07 0.000 4.136508 4.946992

≥70 | 3.47321 .159431 27.12 0.000 3.174375 3.800177

|

Group |

No Diabetes | 1 (base)

Type 2 Diabetes | 1.780343 .1265509 8.11 0.000 1.548811 2.046487

|

cohort#Group |

30-39#Type 2 Diabetes | 1.187102 .093213 2.18 0.029 1.017772 1.384604

40-49#Type 2 Diabetes | 1.032759 .0756357 0.44 0.660 .8946641 1.19217

50-59#Type 2 Diabetes | .8374202 .0604124 -2.46 0.014 .7270041 .9646061

60-69#Type 2 Diabetes | .6795849 .0488068 -5.38 0.000 .5903529 .7823043

≥70#Type 2 Diabetes | .59578 .0430404 -7.17 0.000 .5171224 .686402

|

Kon |

Male | 1 (base)

Female | .7201552 .0044327 -53.34 0.000 .7115196 .7288957

|

Education |

Up to Lower secondary education | 1.083912 .0076531 11.41 0.000 1.069015 1.099016

Upper secondary to Post-secondary ed.. | 1 (base)

Post-secondary ≥2 years to Tertiary .. | .79023 .0063003 -29.53 0.000 .7779775 .8026754

|

No_5p_rank | 1.011269 .001527 7.42 0.000 1.008281 1.014267

_cons | .06719 .0030501 -59.48 0.000 .0614701 .0734421

---------------------------------------------------------------------------------------------------------

Note: _cons estimates baseline odds.

Goodness-of-fit test after logistic model

Variable: everParod2

Table collapsed on quantiles of estimated probabilities

+-------------------------------------------------------------+

| Group | Prob | Obs_1 | Exp_1 | Obs_0 | Exp_0 | Total |

|-------+--------+-------+---------+-------+---------+--------|

| 1 | 0.1156 | 7124 | 7164.3 | 72857 | 72816.7 | 79981 |

| 2 | 0.1439 | 10929 | 11079.6 | 71920 | 71769.4 | 82849 |

| 3 | 0.1541 | 13509 | 13648.4 | 76859 | 76719.6 | 90368 |

| 4 | 0.1638 | 10172 | 9607.8 | 49601 | 50165.2 | 59773 |

| 5 | 0.1892 | 15294 | 15110.6 | 68288 | 68471.4 | 83582 |

|-------+--------+-------+---------+-------+---------+--------|

| 6 | 0.1987 | 18657 | 18423.2 | 75394 | 75627.8 | 94051 |

| 7 | 0.2096 | 9476 | 10024.6 | 39642 | 39093.4 | 49118 |

| 8 | 0.2331 | 25220 | 24971.6 | 86971 | 87219.4 | 112191 |

| 9 | 0.2478 | 10411 | 10768.8 | 34082 | 33724.2 | 44493 |

| 10 | 0.3216 | 20360 | 20353.0 | 53906 | 53913.0 | 74266 |

+-------------------------------------------------------------+

Number of observations = 770,672

Number of groups = 10

Hosmer–Lemeshow chi2(8) = 106.76

Prob > chi2 = 0.0000

### Figure A4. Probability estimates for periodontitis and their contrasts (based on logistic regression, stratified by gender), females and males by age category.

## Figure A5. Prevalence of periodontitis (2010-2020) in subjects with T1D with good/poor glycemic control and matched controls without diabetes, females and males by age category.

## Logistic regression model T1D with good/poor glycemic control versus matched controls without diabetes (outcome: periodontitis)

Logistic regression Number of obs = 25,302

LR chi2(21) = 1220.09

Prob > chi2 = 0.0000

Log likelihood = -8804.1101 Pseudo R2 = 0.0648

---------------------------------------------------------------------------------------------------------

everParod2 | Odds ratio Std. err. z P>|z| [95% conf. interval]

----------------------------------------+----------------------------------------------------------------

cohort |

18-29 | 1 (base)

30-39 | 2.077607 .2086139 7.28 0.000 1.706451 2.529491

40-49 | 2.803505 .2583964 11.18 0.000 2.340168 3.358581

50-59 | 4.977558 .4519379 17.68 0.000 4.166117 5.947046

60-69 | 5.991909 .5632574 19.05 0.000 4.983675 7.204117

≥70 | 4.381787 .5487186 11.80 0.000 3.428133 5.600732

|

Diabetes_Control |

No Diabetes | 1 (base)

Good glycemic control | .8910273 .2241488 -0.46 0.646 .544203 1.458885

Poor glycemic control | 1.68618 .2003936 4.40 0.000 1.335805 2.128456

|

cohort#Diabetes_Control |

30-39#Good glycemic control | .6976811 .2478233 -1.01 0.311 .3477777 1.399627

30-39#Poor glycemic control | .9515281 .1535588 -0.31 0.758 .6935115 1.305538

40-49#Good glycemic control | .7706925 .2594015 -0.77 0.439 .3984573 1.490667

40-49#Poor glycemic control | 1.085081 .1581132 0.56 0.575 .8155095 1.443762

50-59#Good glycemic control | .5968739 .1955601 -1.58 0.115 .3140469 1.134412

50-59#Poor glycemic control | .8155658 .1189835 -1.40 0.162 .6127404 1.085529

60-69#Good glycemic control | .7439913 .2367735 -0.93 0.353 .3987263 1.388228

60-69#Poor glycemic control | .6779316 .1052371 -2.50 0.012 .500094 .9190097

≥70#Good glycemic control | 1.148765 .5454753 0.29 0.770 .4529495 2.913484

≥70#Poor glycemic control | .581413 .1208858 -2.61 0.009 .3868167 .873905

|

Kon |

Male | 1 (base)

Female | .8120334 .0325718 -5.19 0.000 .7506387 .8784495

|

Education |

Up to Lower secondary education | 1.29912 .0681355 4.99 0.000 1.172211 1.439768

Upper secondary to Post-secondary ed.. | 1 (base)

Post-secondary ≥2 years to Tertiary .. | .7045188 .0350536 -7.04 0.000 .6390587 .7766841

|

No_5p_rank | 1.018275 .0095895 1.92 0.054 .9996521 1.037244

_cons | .0503224 .0039796 -37.80 0.000 .043097 .0587593

---------------------------------------------------------------------------------------------------------

Note: _cons estimates baseline odds.

Goodness-of-fit test after logistic model

Variable: everParod2

Table collapsed on quantiles of estimated probabilities

+---------------------------------------------------------+

| Group | Prob | Obs_1 | Exp_1 | Obs_0 | Exp_0 | Total |

|-------+--------+-------+-------+-------+--------+-------|

| 1 | 0.0393 | 99 | 91.9 | 2743 | 2750.1 | 2842 |

| 2 | 0.0505 | 101 | 105.5 | 2176 | 2171.5 | 2277 |

| 3 | 0.0686 | 133 | 151.4 | 2339 | 2320.6 | 2472 |

| 4 | 0.0893 | 178 | 199.3 | 2354 | 2332.7 | 2532 |

| 5 | 0.1028 | 263 | 250.1 | 2312 | 2324.9 | 2575 |

|-------+--------+-------+-------+-------+--------+-------|

| 6 | 0.1253 | 330 | 306.9 | 2226 | 2249.1 | 2556 |

| 7 | 0.1647 | 368 | 362.6 | 2121 | 2126.4 | 2489 |

| 8 | 0.1967 | 479 | 467.1 | 2121 | 2132.9 | 2600 |

| 9 | 0.2298 | 502 | 509.1 | 1928 | 1920.9 | 2430 |

| 10 | 0.3574 | 649 | 658.2 | 1880 | 1870.8 | 2529 |

+---------------------------------------------------------+

Number of observations = 25,302

Number of groups = 10

Hosmer–Lemeshow chi2(8) = 9.10

Prob > chi2 = 0.3341

### Table A11. Risk ratios (T1D with good/poor glycemic control versus matched controls without diabetes)

|  | **Poor glycemic control versus no diabetes** | **Good glycemic control versus no diabetes** |
| --- | --- | --- |
| **Age 18-29 years** | 1.64 (1.31, 2.04) | 0.90 (0.56, 1.44) |
| **Age 30-39 years** | 1.52 (1.26, 1.84) | 0.64 (0.40, 1.02) |
| **Age 40-49 years** | 1.67 (1.45, 1.92) | 0.71 (0.48, 1.06) |
| **Age 50-59 years** | 1.29 (1.13, 1.46) | 0.58 (0.40, 0.84) |
| **Age 60-69 years** | 1.11 (0.95, 1.29) | 0.71 (0.52, 0.99) |
| **Age ≥70 years** | 0.98 (0.74, 1.30) | 1.02 (0.53, 1.96) |
| **Overall** | 1.37 (1.28, 1.47) | 0.71 (0.60, 0.84) |

### Figure A6. Probability estimates for periodontitis (based on logistic regression, stratified by gender), females and males by age category.

## Logistic regression model T1D according to median yearly HbA1c (outcome: periodontitis)

Logistic regression Number of obs = 28,130

LR chi2(39) = 1300.37

Prob > chi2 = 0.0000

Log likelihood = -10004.17 Pseudo R2 = 0.0610

-----------------------------------------------------------------------------------------------------------------------

everParod2 | Odds ratio Std. err. z P>|z| [95% conf. interval]

------------------------------------------------------+----------------------------------------------------------------

cohort |

18-29 | 1 (base)

30-39 | 1.347304 .3515783 1.14 0.253 .8078765 2.246913

40-49 | 2.238241 .5546257 3.25 0.001 1.377156 3.637732

50-59 | 3.025721 .7403349 4.52 0.000 1.87308 4.887663

60-69 | 4.237412 1.023362 5.98 0.000 2.639552 6.802542

≥70 | 2.101838 .86216 1.81 0.070 .9406804 4.696307

|

hba1c_cat |

1 | 1 (base)

2 | .6268012 .2004069 -1.46 0.144 .3349465 1.172963

3 | .8992921 .2107056 -0.45 0.651 .5681499 1.423438

4 | 1.237589 .2463357 1.07 0.284 .8378169 1.828116

5 | 1.312637 .2780011 1.28 0.199 .8667025 1.988012

6 | 1.790957 .3417398 3.05 0.002 1.232151 2.603194

|

cohort#hba1c_cat |

30-39#2 | 1.97987 .8740026 1.55 0.122 .8334482 4.703215

30-39#3 | 1.304794 .4467174 0.78 0.437 .6669898 2.552493

30-39#4 | 1.289706 .3834317 0.86 0.392 .7201565 2.309693

30-39#5 | 1.417811 .441117 1.12 0.262 .7705267 2.608848

30-39#6 | 1.561617 .4479442 1.55 0.120 .8900414 2.739924

40-49#2 | 2.033833 .8381166 1.72 0.085 .9068716 4.561257

40-49#3 | 1.303966 .4180345 0.83 0.408 .6956362 2.444277

40-49#4 | 1.121462 .3128591 0.41 0.681 .6491147 1.937528

40-49#5 | 1.408848 .4089475 1.18 0.238 .7976003 2.488531

40-49#6 | 1.299398 .3508807 0.97 0.332 .765402 2.205946

50-59#2 | 1.83508 .7502632 1.48 0.138 .8234656 4.089446

50-59#3 | 1.625424 .5102988 1.55 0.122 .8784835 3.007459

50-59#4 | 1.424631 .3906296 1.29 0.197 .8323487 2.438368

50-59#5 | 1.440485 .4141522 1.27 0.204 .8199372 2.530677

50-59#6 | 1.234967 .330794 0.79 0.431 .7305592 2.087638

60-69#2 | 2.002088 .812141 1.71 0.087 .9040573 4.433743

60-69#3 | 1.471687 .456859 1.24 0.213 .8008943 2.704306

60-69#4 | 1.241339 .3379624 0.79 0.427 .7280232 2.116583

60-69#5 | 1.044195 .3004958 0.15 0.881 .5940525 1.835432

60-69#6 | .8694577 .2346676 -0.52 0.604 .5122809 1.475668

≥70#2 | 4.337202 2.476687 2.57 0.010 1.416267 13.28233

≥70#3 | 2.05831 .9889806 1.50 0.133 .8026454 5.278344

≥70#4 | 1.73783 .7662025 1.25 0.210 .7323455 4.123811

≥70#5 | 1.526618 .6913648 0.93 0.350 .6284111 3.70866

≥70#6 | 1.132499 .4943542 0.29 0.776 .4813649 2.66441

|

Kon |

Male | 1 (base)

Female | .8095731 .0305403 -5.60 0.000 .7518745 .8716994

|

Education |

Up to Lower secondary education | 1.272783 .0629612 4.88 0.000 1.155175 1.402365

Upper secondary to Post-secondary education <2 years | 1 (base)

Post-secondary ≥2 years to Tertiary education | .7786017 .0358293 -5.44 0.000 .7114514 .85209

|

No_5p_rank | 1.00337 .0093442 0.36 0.718 .985222 1.021853

_cons | .0496615 .0085614 -17.42 0.000 .0354223 .0696247

-----------------------------------------------------------------------------------------------------------------------

Note: _cons estimates baseline odds.

## Figure A7. Prevalence of periodontitis (2010-2020) in T2D with good/poor glycemic control and matched controls without diabetes, females and males by age category.

## Logistic regression model T2D with good/poor glycemic control versus matched controls without diabetes (outcome: periodontitis)

Logistic regression Number of obs = 163,981

LR chi2(21) = 3014.26

Prob > chi2 = 0.0000

Log likelihood = -78940.853 Pseudo R2 = 0.0187

---------------------------------------------------------------------------------------------------------

everParod2 | Odds ratio Std. err. z P>|z| [95% conf. interval]

----------------------------------------+----------------------------------------------------------------

cohort |

18-29 | 1 (base)

30-39 | 1.702383 .2547548 3.56 0.000 1.269633 2.282633

40-49 | 2.502785 .3530183 6.50 0.000 1.898285 3.299785

50-59 | 3.742596 .522319 9.46 0.000 2.846943 4.920023

60-69 | 4.41784 .6148524 10.67 0.000 3.363134 5.803311

≥70 | 3.759718 .5251601 9.48 0.000 2.859294 4.943695

|

Diabetes_Control |

No Diabetes | 1 (base)

Good glycemic control | 1.818708 .4517575 2.41 0.016 1.11771 2.959355

Poor glycemic control | 1.969524 .5222958 2.56 0.011 1.171203 3.312001

|

cohort#Diabetes_Control |

30-39#Good glycemic control | .9029824 .2431458 -0.38 0.705 .5326924 1.530672

30-39#Poor glycemic control | 1.677247 .4816164 1.80 0.072 .9553819 2.944537

40-49#Good glycemic control | .9188281 .2324033 -0.33 0.738 .5596753 1.508455

40-49#Poor glycemic control | 1.323811 .3583087 1.04 0.300 .778818 2.250174

50-59#Good glycemic control | .7706369 .192798 -1.04 0.298 .4719502 1.258356

50-59#Poor glycemic control | .8994431 .241372 -0.39 0.693 .5315544 1.521948

60-69#Good glycemic control | .684664 .1707218 -1.52 0.129 .4199808 1.116158

60-69#Poor glycemic control | .6711535 .1799221 -1.49 0.137 .3968557 1.13504

≥70#Good glycemic control | .6113378 .15288 -1.97 0.049 .3744703 .9980334

≥70#Poor glycemic control | .5596064 .1509048 -2.15 0.031 .3298721 .9493353

|

Kon |

Male | 1 (base)

Female | .7093146 .0091818 -26.53 0.000 .691545 .7275407

|

Education |

Up to Lower secondary education | 1.08593 .0161646 5.54 0.000 1.054706 1.118079

Upper secondary to Post-secondary ed.. | 1 (base)

Post-secondary ≥2 years to Tertiary .. | .7836844 .013284 -14.38 0.000 .7580759 .810158

|

No_5p_rank | 1.005197 .0032256 1.62 0.106 .9988949 1.011539

_cons | .0686563 .0095335 -19.29 0.000 .0522979 .0901314

---------------------------------------------------------------------------------------------------------

Note: _cons estimates baseline odds.

Goodness-of-fit test after logistic model

Variable: everParod2

Table collapsed on quantiles of estimated probabilities

+-----------------------------------------------------------+

| Group | Prob | Obs_1 | Exp_1 | Obs_0 | Exp_0 | Total |

|-------+--------+-------+--------+-------+---------+-------|

| 1 | 0.1250 | 1677 | 1731.1 | 15469 | 15414.9 | 17146 |

| 2 | 0.1542 | 2819 | 2791.9 | 16542 | 16569.1 | 19361 |

| 3 | 0.1659 | 2110 | 2046.2 | 10645 | 10708.8 | 12755 |

| 4 | 0.1771 | 3325 | 3465.2 | 16829 | 16688.8 | 20154 |

| 5 | 0.1921 | 2967 | 2868.7 | 12406 | 12504.3 | 15373 |

|-------+--------+-------+--------+-------+---------+-------|

| 6 | 0.2052 | 3106 | 3065.0 | 11944 | 11985.0 | 15050 |

| 7 | 0.2214 | 3229 | 3294.5 | 12030 | 11964.5 | 15259 |

| 8 | 0.2327 | 4020 | 3983.1 | 13379 | 13415.9 | 17399 |

| 9 | 0.2648 | 4158 | 4149.4 | 12659 | 12667.6 | 16817 |

| 10 | 0.3470 | 4243 | 4258.8 | 10424 | 10408.2 | 14667 |

+-----------------------------------------------------------+

Number of observations = 163,981

Number of groups = 10

Hosmer–Lemeshow chi2(8) = 18.44

Prob > chi2 = 0.0181

### Table A12. Risk ratios (T2D with good/poor glycemic control versus matched controls without diabetes)

|  | **Poor glycemic control versus no diabetes** | **Good glycemic control versus no diabetes** |
| --- | --- | --- |
| **Age 18-29 years** | 1.87 (1.16, 2.99) | 1.74 (1.11, 2.71) |
| **Age 30-39 years** | 2.72 (2.29, 3.23) | 1.55 (1.30, 1.85) |
| **Age 40-49 years** | 2.16 (1.99, 2.34) | 1.54 (1.42, 1.66) |
| **Age 50-59 years** | 1.55 (1.46, 1.65) | 1.31 (1.25, 1.37) |
| **Age 60-69 years** | 1.24 (1.17, 1.31) | 1.18 (1.15, 1.22) |
| **Age ≥70 years** | 1.08 (1.00, 1.17) | 1.09 (1.04, 1.14) |
| **Overall** | 1.38 (1.33, 1.43) | 1.23 (1.20, 1.26) |

### Figure A8. Probability estimates for periodontitis (based on logistic regression, stratified by gender), females and males by age category.

## Logistic regression model T2D according to median yearly HbA1c (outcome: periodontitis)

Logistic regression Number of obs = 240,307

LR chi2(39) = 3300.89

Prob > chi2 = 0.0000

Log likelihood = -124115.08 Pseudo R2 = 0.0131

-----------------------------------------------------------------------------------------------------------------------

everParod2 | Odds ratio Std. err. z P>|z| [95% conf. interval]

------------------------------------------------------+----------------------------------------------------------------

cohort |

18-29 | 1 (base)

30-39 | 2.191313 .2366705 7.26 0.000 1.773255 2.707931

40-49 | 3.12743 .3180317 11.21 0.000 2.562288 3.81722

50-59 | 3.797523 .3810246 13.30 0.000 3.119572 4.622807

60-69 | 4.112625 .4107689 14.16 0.000 3.381436 5.001924

≥70 | 2.878174 .2897382 10.50 0.000 2.36281 3.505947

|

hba1c_cat |

1 | 1 (base)

2 | 1.361426 .2833762 1.48 0.138 .9053563 2.047241

3 | 1.277057 .2466087 1.27 0.205 .8746564 1.86459

4 | 1.664921 .2939852 2.89 0.004 1.177855 2.353398

5 | 1.389114 .31373 1.46 0.146 .8922673 2.162624

6 | 1.931245 .2802244 4.54 0.000 1.453208 2.566534

|

cohort#hba1c_cat |

30-39#2 | .9111096 .2071896 -0.41 0.682 .5834509 1.422777

30-39#3 | 1.026223 .2147926 0.12 0.902 .6808974 1.546684

30-39#4 | .7509925 .1459219 -1.47 0.141 .5131479 1.099079

30-39#5 | 1.021556 .2525195 0.09 0.931 .6292928 1.658332

30-39#6 | .8148126 .1323178 -1.26 0.207 .5926942 1.120172

40-49#2 | .7894536 .1684166 -1.11 0.268 .5196814 1.199268

40-49#3 | .8076952 .1597715 -1.08 0.280 .5481129 1.190214

40-49#4 | .6563455 .1191153 -2.32 0.020 .4598902 .9367222

40-49#5 | .8847942 .2054342 -0.53 0.598 .5613165 1.394687

40-49#6 | .6598311 .09962 -2.75 0.006 .4908175 .8870447

50-59#2 | .7628093 .1606973 -1.29 0.199 .5047746 1.152748

50-59#3 | .8344131 .1629973 -0.93 0.354 .5689895 1.223652

50-59#4 | .682295 .122144 -2.14 0.033 .4803864 .9690668

50-59#5 | .8966097 .2056626 -0.48 0.634 .5719478 1.405563

50-59#6 | .6497179 .0967614 -2.90 0.004 .4852402 .8699472

60-69#2 | .7045081 .1478398 -1.67 0.095 .4669418 1.062941

60-69#3 | .7557112 .1470993 -1.44 0.150 .5160233 1.106732

60-69#4 | .5895348 .1051306 -2.96 0.003 .4156391 .8361852

60-69#5 | .7738327 .1769478 -1.12 0.262 .4943196 1.211397

60-69#6 | .5301904 .0789174 -4.26 0.000 .3960343 .7097918

≥70#2 | .7108733 .1499902 -1.62 0.106 .4701036 1.074956

≥70#3 | .7534346 .1473708 -1.45 0.148 .5135133 1.105451

≥70#4 | .5665932 .1015507 -3.17 0.002 .3987587 .8050678

≥70#5 | .6535014 .1502837 -1.85 0.064 .4163882 1.025639

≥70#6 | .4900729 .0738813 -4.73 0.000 .3647011 .6585433

|

Kon |

Male | 1 (base)

Female | .7079629 .0072989 -33.50 0.000 .6938009 .722414

|

Education |

Up to Lower secondary education | 1.099737 .0125347 8.34 0.000 1.075441 1.124581

Upper secondary to Post-secondary education <2 years | 1 (base)

Post-secondary ≥2 years to Tertiary education | .8818745 .0126065 -8.79 0.000 .8575092 .9069322

|

No_5p_rank | 1.005333 .0023074 2.32 0.020 1.00082 1.009865

_cons | .0892719 .008861 -24.34 0.000 .0734895 .1084437

-----------------------------------------------------------------------------------------------------------------------

Note: _cons estimates baseline odds.

# Diabetes and tooth loss

## Figure A9. Prevalence of tooth loss (2010-2020) in T1D and matched controls without diabetes, females and males by age category.

## Poisson regression model T1D versus matched controls without diabetes (outcome: tooth loss)

Poisson regression Number of obs = 86,273

LR chi2(16) = 22796.97

Prob > chi2 = 0.0000

Log likelihood = -108311.34 Pseudo R2 = 0.0952

-----------------------------------------------------------------------------------------------------------------------

total_ex_any | IRR Std. err. z P>|z| [95% conf. interval]

------------------------------------------------------+----------------------------------------------------------------

cohort |

18-29 | 1 (base)

30-39 | 1.26139 .0270857 10.81 0.000 1.209404 1.31561

40-49 | 1.602032 .0306066 24.67 0.000 1.543153 1.663157

50-59 | 2.240376 .0414724 43.58 0.000 2.160549 2.323153

60-69 | 2.379442 .0455687 45.26 0.000 2.291784 2.470452

≥70 | 2.266803 .0511388 36.28 0.000 2.168756 2.369282

|

Group |

No Diabetes | 1 (base)

Type 1 Diabetes | 1.516422 .0325377 19.40 0.000 1.453972 1.581555

|

cohort#Group |

30-39#Type 1 Diabetes | .9399376 .0305802 -1.90 0.057 .8818725 1.001826

40-49#Type 1 Diabetes | .9119992 .0263441 -3.19 0.001 .8618002 .9651222

50-59#Type 1 Diabetes | .7462661 .0213745 -10.22 0.000 .705527 .7893577

60-69#Type 1 Diabetes | .7936694 .0232492 -7.89 0.000 .7493852 .8405706

≥70#Type 1 Diabetes | .7563702 .0271983 -7.77 0.000 .7048976 .8116015

|

Kon |

Male | 1 (base)

Female | .96253 .008354 -4.40 0.000 .946295 .9790435

|

Education |

Up to Lower secondary education | 1.28227 .0142967 22.30 0.000 1.254553 1.3106

Upper secondary to Post-secondary education <2 years | 1 (base)

Post-secondary ≥2 years to Tertiary education | .673014 .0075058 -35.51 0.000 .6584625 .6878871

|

maximum_AT | .9495241 .0005248 -93.71 0.000 .948496 .9505533

No_5p_rank | 1.048622 .0019705 25.27 0.000 1.044767 1.052491

_cons | .2125596 .004399 -74.83 0.000 .2041103 .2213586

ln(years_followup) | 1 (exposure)

-----------------------------------------------------------------------------------------------------------------------

Note: _cons estimates baseline incidence rate.

## Multinomial logistic regression model T1D versus matched controls without diabetes (outcome: tooth loss, categorical - no extraction, 1-4 extractions, ≥5 extractions)

Multinomial logistic regression Number of obs = 86,273

LR chi2(34) = 10382.74

Prob > chi2 = 0.0000

Log likelihood = -55087.187 Pseudo R2 = 0.0861

-----------------------------------------------------------------------------------------------------------------------

ex | RRR Std. err. z P>|z| [95% conf. interval]

------------------------------------------------------+----------------------------------------------------------------

0 | (base outcome)

------------------------------------------------------+----------------------------------------------------------------

1 |

cohort |

18-29 | 1 (base)

30-39 | 1.301534 .0422248 8.12 0.000 1.221351 1.386981

40-49 | 1.671736 .050689 16.95 0.000 1.575282 1.774096

50-59 | 2.266928 .0711586 26.07 0.000 2.131664 2.410776

60-69 | 2.649696 .0890743 28.99 0.000 2.480741 2.830159

≥70 | 2.500326 .1094496 20.94 0.000 2.294753 2.724314

|

Group |

No Diabetes | 1 (base)

Type 1 Diabetes | 1.3219 .0453647 8.13 0.000 1.235911 1.413872

|

cohort#Group |

30-39#Type 1 Diabetes | 1.05507 .0560363 1.01 0.313 .9507645 1.17082

40-49#Type 1 Diabetes | .9453756 .0473404 -1.12 0.262 .856998 1.042867

50-59#Type 1 Diabetes | .8588963 .0449838 -2.90 0.004 .775104 .9517471

60-69#Type 1 Diabetes | .884129 .0495536 -2.20 0.028 .7921502 .9867876

≥70#Type 1 Diabetes | .7268355 .053996 -4.29 0.000 .6283492 .8407583

|

Kon |

Male | 1 (base)

Female | 1.03078 .0166276 1.88 0.060 .9987001 1.06389

|

Education |

Up to Lower secondary education | 1.209385 .0289868 7.93 0.000 1.153886 1.267554

Upper secondary to Post-secondary education <2 years | 1 (base)

Post-secondary ≥2 years to Tertiary education | .6775641 .0124952 -21.11 0.000 .6535112 .7025022

|

maximum_AT | .9705566 .0013878 -20.90 0.000 .9678404 .9732804

No_5p_rank | 1.051587 .004375 12.09 0.000 1.043047 1.060197

years_followup | 1.160388 .0027748 62.21 0.000 1.154962 1.16584

_cons | .1990989 .0092145 -34.87 0.000 .1818337 .2180034

------------------------------------------------------+----------------------------------------------------------------

2 |

cohort |

18-29 | 1 (base)

30-39 | 2.547523 .4383782 5.43 0.000 1.818207 3.569382

40-49 | 4.469039 .6815012 9.82 0.000 3.314447 6.025834

50-59 | 9.077092 1.326862 15.09 0.000 6.81586 12.08851

60-69 | 9.303989 1.377228 15.07 0.000 6.960968 12.43566

≥70 | 9.197442 1.434235 14.23 0.000 6.775354 12.48539

|

Group |

No Diabetes | 1 (base)

Type 1 Diabetes | 3.482451 .5878178 7.39 0.000 2.501538 4.848004

|

cohort#Group |

30-39#Type 1 Diabetes | .6289339 .1438846 -2.03 0.043 .4016715 .9847794

40-49#Type 1 Diabetes | .6201846 .1237597 -2.39 0.017 .4194316 .9170243

50-59#Type 1 Diabetes | .4203357 .0813704 -4.48 0.000 .2876186 .6142931

60-69#Type 1 Diabetes | .4214018 .0827304 -4.40 0.000 .286805 .6191644

≥70#Type 1 Diabetes | .3115711 .0666433 -5.45 0.000 .2048754 .4738322

|

Kon |

Male | 1 (base)

Female | .8638233 .0413851 -3.06 0.002 .7864018 .948867

|

Education |

Up to Lower secondary education | 1.670877 .0937169 9.15 0.000 1.496931 1.865035

Upper secondary to Post-secondary education <2 years | 1 (base)

Post-secondary ≥2 years to Tertiary education | .4326162 .030539 -11.87 0.000 .376717 .4968101

|

maximum_AT | .9040575 .0024005 -37.99 0.000 .8993648 .9087747

No_5p_rank | 1.086892 .0106627 8.49 0.000 1.066193 1.107993

years_followup | 1.169838 .0084214 21.79 0.000 1.153448 1.186461

_cons | .0236564 .003619 -24.47 0.000 .0175278 .0319277

-----------------------------------------------------------------------------------------------------------------------

Note: _cons estimates baseline relative risk for each outcome.

### Figure A10. Estimated annual incidence rate of tooth loss (based on Poisson regression, stratified by gender) and probability estimates for tooth loss (based on multinomial logistic regression, stratified by gender) in T1D and controls without diabetes, females and males by age category.

## Figure A11. Prevalence of tooth loss (2010-2020) in T2D and matched controls without diabetes, females and males by age category.

## Poisson regression model T2D versus matched controls without diabetes (outcome: tooth loss)

Poisson regression Number of obs = 786,305

LR chi2(16) = 195753.70

Prob > chi2 = 0.0000

Log likelihood = -1321387.9 Pseudo R2 = 0.0690

-----------------------------------------------------------------------------------------------------------------------

total_ex_any | IRR Std. err. z P>|z| [95% conf. interval]

------------------------------------------------------+----------------------------------------------------------------

cohort |

18-29 | 1 (base)

30-39 | 1.167662 .0221978 8.15 0.000 1.124955 1.211989

40-49 | 1.485801 .0257138 22.88 0.000 1.436248 1.537063

50-59 | 1.969596 .0333798 40.00 0.000 1.905248 2.036118

60-69 | 2.262443 .0381273 48.45 0.000 2.188935 2.338419

≥70 | 2.221351 .037609 47.14 0.000 2.148848 2.2963

|

Group |

No Diabetes | 1 (base)

Type 2 Diabetes | 2.268554 .0545928 34.04 0.000 2.164038 2.378117

|

cohort#Group |

30-39#Type 2 Diabetes | 1.079502 .0295356 2.80 0.005 1.023138 1.138971

40-49#Type 2 Diabetes | .8986358 .0224803 -4.27 0.000 .8556379 .9437944

50-59#Type 2 Diabetes | .6866333 .0168448 -15.32 0.000 .6543992 .7204551

60-69#Type 2 Diabetes | .5554402 .0135447 -24.11 0.000 .5295175 .582632

≥70#Type 2 Diabetes | .4888739 .0119711 -29.23 0.000 .4659651 .5129091

|

Kon |

Male | 1 (base)

Female | .89955 .0021068 -45.20 0.000 .8954303 .9036887

|

Education |

Up to Lower secondary education | 1.091425 .0028975 32.95 0.000 1.085761 1.097119

Upper secondary to Post-secondary education <2 years | 1 (base)

Post-secondary ≥2 years to Tertiary education | .8248373 .0027077 -58.66 0.000 .8195474 .8301613

|

maximum_AT | .9534952 .0001343 -338.10 0.000 .953232 .9537585

No_5p_rank | 1.031566 .000526 60.95 0.000 1.030535 1.032597

_cons | .2218592 .0037908 -88.12 0.000 .2145523 .2294149

ln(years_followup) | 1 (exposure)

-----------------------------------------------------------------------------------------------------------------------

Note: _cons estimates baseline incidence rate.

## Multinomial logistic regression model T2D versus matched controls without diabetes (outcome: tooth loss, categorical - no extraction, 1-4 extractions, ≥5 extractions)

Multinomial logistic regression Number of obs = 786,305

LR chi2(34) = 103807.63

Prob > chi2 = 0.0000

Log likelihood = -594097.74 Pseudo R2 = 0.0803

-----------------------------------------------------------------------------------------------------------------------

ex | RRR Std. err. z P>|z| [95% conf. interval]

------------------------------------------------------+----------------------------------------------------------------

0 | (base outcome)

------------------------------------------------------+----------------------------------------------------------------

1 |

cohort |

18-29 | 1 (base)

30-39 | 1.245118 .036196 7.54 0.000 1.176158 1.318121

40-49 | 1.56397 .0415777 16.82 0.000 1.484566 1.647622

50-59 | 2.040037 .0531182 27.38 0.000 1.938539 2.146849

60-69 | 2.482914 .0642487 35.15 0.000 2.360129 2.612087

≥70 | 2.518754 .0659597 35.28 0.000 2.392737 2.651408

|

Group |

No Diabetes | 1 (base)

Type 2 Diabetes | 2.242427 .0965605 18.75 0.000 2.060938 2.439898

|

cohort#Group |

30-39#Type 2 Diabetes | .9435305 .0470504 -1.17 0.244 .8556767 1.040404

40-49#Type 2 Diabetes | .8059612 .0366247 -4.75 0.000 .737282 .881038

50-59#Type 2 Diabetes | .6569772 .0291958 -9.45 0.000 .6021757 .7167659

60-69#Type 2 Diabetes | .54013 .0238036 -13.98 0.000 .495434 .5888584

≥70#Type 2 Diabetes | .4657739 .0206617 -17.22 0.000 .4269881 .5080828

|

Kon |

Male | 1 (base)

Female | .9333044 .0047563 -13.54 0.000 .9240286 .9426733

|

Education |

Up to Lower secondary education | 1.055868 .0065147 8.81 0.000 1.043176 1.068714

Upper secondary to Post-secondary education <2 years | 1 (base)

Post-secondary ≥2 years to Tertiary education | .8275829 .0053284 -29.39 0.000 .8172051 .8380925

|

maximum_AT | .9771219 .0003803 -59.46 0.000 .9763768 .9778676

No_5p_rank | 1.023342 .0013389 17.64 0.000 1.020722 1.02597

years_followup | 1.186804 .000855 237.72 0.000 1.18513 1.188481

_cons | .1658509 .0046024 -64.74 0.000 .1570713 .1751212

------------------------------------------------------+----------------------------------------------------------------

2 |

cohort |

18-29 | 1 (base)

30-39 | 1.443491 .2028295 2.61 0.009 1.095996 1.901162

40-49 | 2.805293 .3595741 8.05 0.000 2.182096 3.606473

50-59 | 5.101211 .6441001 12.91 0.000 3.982878 6.533555

60-69 | 6.765135 .8512576 15.19 0.000 5.286518 8.657316

≥70 | 6.384472 .8044898 14.71 0.000 4.987322 8.17302

|

Group |

No Diabetes | 1 (base)

Type 2 Diabetes | 5.224025 .8305096 10.40 0.000 3.825447 7.133921

|

cohort#Group |

30-39#Type 2 Diabetes | 1.269003 .225058 1.34 0.179 .8964004 1.796485

40-49#Type 2 Diabetes | .8353278 .1361947 -1.10 0.270 .6068398 1.149846

50-59#Type 2 Diabetes | .4850014 .0779466 -4.50 0.000 .3539507 .6645737

60-69#Type 2 Diabetes | .3214676 .0514615 -7.09 0.000 .2348953 .4399468

≥70#Type 2 Diabetes | .2349253 .0376644 -9.03 0.000 .1715776 .3216613

|

Kon |

Male | 1 (base)

Female | .8122571 .0092595 -18.24 0.000 .7943101 .8306096

|

Education |

Up to Lower secondary education | 1.199257 .0150163 14.51 0.000 1.170183 1.229052

Upper secondary to Post-secondary education <2 years | 1 (base)

Post-secondary ≥2 years to Tertiary education | .6166008 .0106884 -27.89 0.000 .5960038 .6379095

|

maximum_AT | .9130944 .0005956 -139.38 0.000 .9119279 .9142625

No_5p_rank | 1.060133 .0025078 24.69 0.000 1.055229 1.06506

years_followup | 1.216426 .0020701 115.12 0.000 1.212375 1.22049

_cons | .0259885 .0032903 -28.83 0.000 .0202775 .0333079

-----------------------------------------------------------------------------------------------------------------------

Note: _cons estimates baseline relative risk for each outcome.

### Figure A12. Estimated annual incidence rate of tooth loss (based on Poisson regression, stratified by gender) and probability estimates for tooth loss (based on multinomial logistic regression, stratified by gender) in T2D and controls without diabetes, females and males by age category.

## Figure A13. Prevalence of tooth loss (2010-2020) in T1D with good/poor glycemic control and matched controls without diabetes, females and males by age category.

## Poisson regression model T1D with good/poor glycemic control versus matched controls without diabetes (outcome: tooth loss)

Poisson regression Number of obs = 25,683

LR chi2(22) = 8178.69

Prob > chi2 = 0.0000

Log likelihood = -34021.236 Pseudo R2 = 0.1073

-----------------------------------------------------------------------------------------------------------------------

total_ex_any | IRR Std. err. z P>|z| [95% conf. interval]

------------------------------------------------------+----------------------------------------------------------------

cohort |

18-29 | 1 (base)

30-39 | 1.214734 .0474491 4.98 0.000 1.125206 1.311385

40-49 | 1.597012 .0548706 13.63 0.000 1.493009 1.70826

50-59 | 2.14045 .0733573 22.21 0.000 2.001395 2.289166

60-69 | 2.453886 .0878675 25.07 0.000 2.287573 2.63229

≥70 | 2.223956 .1035115 17.17 0.000 2.030056 2.436376

|

Diabetes_Control |

No Diabetes | 1 (base)

Good glycemic control | .8175985 .0748869 -2.20 0.028 .6832434 .9783737

Poor glycemic control | 2.510705 .0917036 25.20 0.000 2.337252 2.697031

|

cohort#Diabetes_Control |

30-39#Good glycemic control | 1.053379 .1406832 0.39 0.697 .8107801 1.368567

30-39#Poor glycemic control | .9130371 .0501537 -1.66 0.098 .8198443 1.016823

40-49#Good glycemic control | .8761499 .1151447 -1.01 0.314 .677193 1.13356

40-49#Poor glycemic control | .7828119 .0377901 -5.07 0.000 .7121408 .8604962

50-59#Good glycemic control | 1.123144 .1337226 0.98 0.329 .8893867 1.41834

50-59#Poor glycemic control | .5775416 .0285144 -11.12 0.000 .5242733 .6362223

60-69#Good glycemic control | 1.07822 .1308024 0.62 0.535 .8500518 1.367633

60-69#Poor glycemic control | .5355006 .0282457 -11.84 0.000 .4829056 .593824

≥70#Good glycemic control | 1.212922 .2485858 0.94 0.346 .8116723 1.812528

≥70#Poor glycemic control | .4888312 .0331556 -10.55 0.000 .4279815 .5583323

|

Kon |

Male | 1 (base)

Female | .9758769 .0147643 -1.61 0.107 .9473642 1.005248

|

Education |

Up to Lower secondary education | 1.259997 .024233 12.02 0.000 1.213385 1.308399

Upper secondary to Post-secondary education <2 years | 1 (base)

Post-secondary ≥2 years to Tertiary education | .672721 .0136437 -19.55 0.000 .6465043 .7000007

|

maximum_AT | .9521084 .0009116 -51.26 0.000 .9503234 .9538968

No_5p_rank | 1.040695 .0032134 12.92 0.000 1.034416 1.047012

_cons | .1984573 .0073211 -43.84 0.000 .1846146 .2133379

ln(years_followup) | 1 (exposure)

-----------------------------------------------------------------------------------------------------------------------

Note: _cons estimates baseline incidence rate.

## Multinomial logistic regression model T1D with good/poor glycemic control versus matched controls without diabetes (outcome: tooth loss, categorical - no extraction, 1-4 extractions, ≥5 extractions)

Multinomial logistic regression Number of obs = 25,683

LR chi2(46) = 3479.58

Prob > chi2 = 0.0000

Log likelihood = -16840.474 Pseudo R2 = 0.0936

-----------------------------------------------------------------------------------------------------------------------

ex | RRR Std. err. z P>|z| [95% conf. interval]

------------------------------------------------------+----------------------------------------------------------------

0 | (base outcome)

------------------------------------------------------+----------------------------------------------------------------

1 |

cohort |

18-29 | 1 (base)

30-39 | 1.266244 .0738468 4.05 0.000 1.129472 1.419577

40-49 | 1.575817 .0853031 8.40 0.000 1.417189 1.752199

50-59 | 2.006312 .1161044 12.03 0.000 1.791182 2.24728

60-69 | 2.609146 .1655708 15.11 0.000 2.304003 2.954703

≥70 | 2.557975 .2350858 10.22 0.000 2.136329 3.06284

|

Diabetes_Control |

No Diabetes | 1 (base)

Good glycemic control | .8492123 .10621 -1.31 0.191 .6645956 1.085113

Poor glycemic control | 2.172536 .1407825 11.97 0.000 1.913411 2.466753

|

cohort#Diabetes_Control |

30-39#Good glycemic control | 1.166842 .2159403 0.83 0.404 .8118675 1.677021

30-39#Poor glycemic control | .9815739 .0980355 -0.19 0.852 .807065 1.193816

40-49#Good glycemic control | .9688348 .183778 -0.17 0.867 .668014 1.405122

40-49#Poor glycemic control | .8090039 .0751418 -2.28 0.022 .6743562 .9705365

50-59#Good glycemic control | 1.380647 .2604817 1.71 0.087 .9538707 1.998369

50-59#Poor glycemic control | .6673799 .0664819 -4.06 0.000 .5490092 .8112722

60-69#Good glycemic control | 1.04791 .2072231 0.24 0.813 .7112131 1.544003

60-69#Poor glycemic control | .5954403 .0664688 -4.64 0.000 .4784305 .7410671

≥70#Good glycemic control | .8768436 .3240015 -0.36 0.722 .4250065 1.809042

≥70#Poor glycemic control | .3998611 .0622367 -5.89 0.000 .2947292 .5424942

|

Kon |

Male | 1 (base)

Female | 1.044006 .0306951 1.46 0.143 .9855457 1.105935

|

Education |

Up to Lower secondary education | 1.187827 .0513745 3.98 0.000 1.091284 1.29291

Upper secondary to Post-secondary education <2 years | 1 (base)

Post-secondary ≥2 years to Tertiary education | .6807665 .0233056 -11.23 0.000 .6365872 .728012

|

maximum_AT | .9714477 .0025184 -11.17 0.000 .9665243 .9763962

No_5p_rank | 1.046308 .0075347 6.29 0.000 1.031644 1.06118

years_followup | 1.160549 .0050675 34.10 0.000 1.150659 1.170524

_cons | .200077 .0168114 -19.15 0.000 .1696974 .2358952

------------------------------------------------------+----------------------------------------------------------------

2 |

cohort |

18-29 | 1 (base)

30-39 | 2.346646 .7920318 2.53 0.011 1.211024 4.54718

40-49 | 5.200822 1.509589 5.68 0.000 2.944438 9.186319

50-59 | 10.25389 2.901789 8.23 0.000 5.888489 17.85557

60-69 | 10.35553 3.008838 8.05 0.000 5.859391 18.30173

≥70 | 10.51542 3.338251 7.41 0.000 5.644197 19.59076

|

Diabetes_Control |

No Diabetes | 1 (base)

Good glycemic control | 1.17e-06 .0009043 -0.02 0.986 0 .

Poor glycemic control | 10.97976 3.235812 8.13 0.000 6.162233 19.56356

|

cohort#Diabetes_Control |

30-39#Good glycemic control | 769379.4 5.96e+08 0.02 0.986 0 .

30-39#Poor glycemic control | .6057881 .2388404 -1.27 0.204 .2797195 1.311954

40-49#Good glycemic control | 506603.7 3.92e+08 0.02 0.986 0 .

40-49#Poor glycemic control | .3792475 .1288195 -2.85 0.004 .1948919 .7379917

50-59#Good glycemic control | 625201.2 4.84e+08 0.02 0.986 0 .

50-59#Poor glycemic control | .202724 .0681051 -4.75 0.000 .1049408 .3916211

60-69#Good glycemic control | 646829.2 5.01e+08 0.02 0.986 0 .

60-69#Poor glycemic control | .1821069 .0640142 -4.85 0.000 .0914349 .3626942

≥70#Good glycemic control | 480722.2 3.72e+08 0.02 0.987 0 .

≥70#Poor glycemic control | .1564079 .060838 -4.77 0.000 .0729738 .3352361

|

Kon |

Male | 1 (base)

Female | .8564325 .0695117 -1.91 0.056 .7304762 1.004108

|

Education |

Up to Lower secondary education | 1.634085 .1547012 5.19 0.000 1.357345 1.967248

Upper secondary to Post-secondary education <2 years | 1 (base)

Post-secondary ≥2 years to Tertiary education | .3979255 .0511511 -7.17 0.000 .3093033 .5119398

|

maximum_AT | .9108848 .0042162 -20.17 0.000 .9026585 .9191861

No_5p_rank | 1.072559 .0169635 4.43 0.000 1.039821 1.106327

years_followup | 1.171413 .0144879 12.79 0.000 1.143358 1.200155

_cons | .017953 .0052453 -13.76 0.000 .0101261 .0318297

-----------------------------------------------------------------------------------------------------------------------

Note: _cons estimates baseline relative risk for each outcome.

### Figure A14. Probability estimates for tooth loss (based on multinomial logistic regression) in T1D with good/poor glycemic control and matched controls without diabetes, by age category.

### Figure A15. Estimated annual incidence rate of tooth loss (based on Poisson regression, stratified by gender) in T1D with good/poor glycemic control and matched controls without diabetes, females and males by age category.

## Figure A16. Prevalence of tooth loss (2010-2020) in T2D with good/poor glycemic control and matched controls without diabetes, females and males by age category.

## Poisson regression model T2D with good/poor glycemic control versus matched controls without diabetes (outcome: tooth loss)

Poisson regression Number of obs = 167,362

LR chi2(22) = 46014.65

Prob > chi2 = 0.0000

Log likelihood = -281481.23 Pseudo R2 = 0.0756

-----------------------------------------------------------------------------------------------------------------------

total_ex_any | IRR Std. err. z P>|z| [95% conf. interval]

------------------------------------------------------+----------------------------------------------------------------

cohort |

18-29 | 1 (base)

30-39 | 1.309136 .076325 4.62 0.000 1.167773 1.467613

40-49 | 1.65734 .0900619 9.30 0.000 1.489897 1.843601

50-59 | 2.200899 .1180298 14.71 0.000 1.981307 2.444828

60-69 | 2.639027 .1410083 18.16 0.000 2.376635 2.930388

≥70 | 2.554277 .1369385 17.49 0.000 2.299502 2.837279

|

Diabetes_Control |

No Diabetes | 1 (base)

Good glycemic control | 1.775797 .1660569 6.14 0.000 1.478415 2.132996

Poor glycemic control | 4.112478 .3335796 17.43 0.000 3.507997 4.821121

|

cohort#Diabetes_Control |

30-39#Good glycemic control | 1.043508 .1061688 0.42 0.676 .8548555 1.273793

30-39#Poor glycemic control | 1.136255 .1013559 1.43 0.152 .9539972 1.353332

40-49#Good glycemic control | .9541886 .0910499 -0.49 0.623 .7914281 1.150421

40-49#Poor glycemic control | .8999251 .0748191 -1.27 0.205 .7646067 1.059192

50-59#Good glycemic control | .7290541 .0687531 -3.35 0.001 .6060209 .8770651

50-59#Poor glycemic control | .6092117 .0501004 -6.03 0.000 .5185219 .7157632

60-69#Good glycemic control | .598434 .0562053 -5.47 0.000 .4978183 .7193855

60-69#Poor glycemic control | .3976949 .0326706 -11.22 0.000 .3385509 .4671712

≥70#Good glycemic control | .5563044 .0523649 -6.23 0.000 .4625822 .6690154

≥70#Poor glycemic control | .3156598 .026092 -13.95 0.000 .268448 .3711748

|

Kon |

Male | 1 (base)

Female | .8919428 .0044515 -22.91 0.000 .8832606 .9007104

|

Education |

Up to Lower secondary education | 1.077024 .0060828 13.14 0.000 1.065168 1.089012

Upper secondary to Post-secondary education <2 years | 1 (base)

Post-secondary ≥2 years to Tertiary education | .8427826 .0059322 -24.30 0.000 .8312355 .8544901

|

maximum_AT | .9544066 .0002876 -154.88 0.000 .9538431 .9549704

No_5p_rank | 1.035262 .0010944 32.78 0.000 1.03312 1.037409

_cons | .1891663 .0101728 -30.96 0.000 .1702428 .2101932

ln(years_followup) | 1 (exposure)

-----------------------------------------------------------------------------------------------------------------------

Note: _cons estimates baseline incidence rate.

## Multinomial logistic regression model T2D with good/poor glycemic control versus matched controls without diabetes (outcome: tooth loss, categorical - no extraction, 1-4 extractions, ≥5 extractions)

Multinomial logistic regression Number of obs = 167,362

LR chi2(46) = 24109.49

Prob > chi2 = 0.0000

Log likelihood = -126462.81 Pseudo R2 = 0.0870

-----------------------------------------------------------------------------------------------------------------------

ex | RRR Std. err. z P>|z| [95% conf. interval]

------------------------------------------------------+----------------------------------------------------------------

0 | (base outcome)

------------------------------------------------------+----------------------------------------------------------------

1 |

cohort |

18-29 | 1 (base)

30-39 | 1.460426 .128547 4.30 0.000 1.229013 1.735411

40-49 | 1.749323 .1436888 6.81 0.000 1.489199 2.054884

50-59 | 2.319812 .1877886 10.40 0.000 1.979467 2.718677

60-69 | 2.869114 .2312241 13.08 0.000 2.449902 3.360058

≥70 | 2.89644 .235315 13.09 0.000 2.470077 3.396398

|

Diabetes_Control |

No Diabetes | 1 (base)

Good glycemic control | 1.942406 .3026937 4.26 0.000 1.431178 2.63625

Poor glycemic control | 3.685406 .6321375 7.60 0.000 2.633198 5.158071

|

cohort#Diabetes_Control |

30-39#Good glycemic control | .8490337 .1468017 -0.95 0.344 .6049903 1.19152

30-39#Poor glycemic control | .9271084 .1831143 -0.38 0.702 .6295188 1.365376

40-49#Good glycemic control | .8055841 .1294998 -1.34 0.179 .5878655 1.103936

40-49#Poor glycemic control | .8471019 .1516159 -0.93 0.354 .5964664 1.203055

50-59#Good glycemic control | .6644168 .1050324 -2.59 0.010 .4873948 .9057332

50-59#Poor glycemic control | .5909294 .1041304 -2.99 0.003 .4183515 .8346988

60-69#Good glycemic control | .5592127 .0878569 -3.70 0.000 .4110042 .7608653

60-69#Poor glycemic control | .3777197 .0663496 -5.54 0.000 .2677004 .5329546

≥70#Good glycemic control | .5173066 .0816275 -4.18 0.000 .3796943 .7047936

≥70#Poor glycemic control | .3301097 .0583363 -6.27 0.000 .2334725 .4667463

|

Kon |

Male | 1 (base)

Female | .9386101 .0103329 -5.75 0.000 .9185749 .9590823

|

Education |

Up to Lower secondary education | 1.047035 .0139158 3.46 0.001 1.020112 1.074667

Upper secondary to Post-secondary education <2 years | 1 (base)

Post-secondary ≥2 years to Tertiary education | .8231902 .0115203 -13.90 0.000 .8009176 .8460822

|

maximum_AT | .9758209 .0008217 -29.07 0.000 .9742118 .9774327

No_5p_rank | 1.02764 .0028959 9.68 0.000 1.02198 1.033332

years_followup | 1.193758 .0018672 113.23 0.000 1.190104 1.197423

_cons | .1429248 .0119464 -23.27 0.000 .1213276 .1683664

------------------------------------------------------+----------------------------------------------------------------

2 |

cohort |

18-29 | 1 (base)

30-39 | 1.175118 .4214759 0.45 0.653 .5818133 2.373446

40-49 | 1.988471 .6516826 2.10 0.036 1.046055 3.779932

50-59 | 3.655348 1.180903 4.01 0.000 1.940609 6.885245

60-69 | 5.295101 1.70495 5.18 0.000 2.817079 9.952896

≥70 | 4.903662 1.581848 4.93 0.000 2.605768 9.227952

|

Diabetes_Control |

No Diabetes | 1 (base)

Good glycemic control | 1.757159 1.060454 0.93 0.350 .5384023 5.734761

Poor glycemic control | 12.68289 5.428544 5.93 0.000 5.481327 29.3461

|

cohort#Diabetes_Control |

30-39#Good glycemic control | 2.312016 1.491553 1.30 0.194 .6529116 8.187045

30-39#Poor glycemic control | 1.875612 .8921567 1.32 0.186 .7383445 4.764606

40-49#Good glycemic control | 2.092873 1.279347 1.21 0.227 .6315588 6.935406

40-49#Poor glycemic control | 1.030948 .4521737 0.07 0.945 .436415 2.435419

50-59#Good glycemic control | .9856135 .5979369 -0.02 0.981 .3001323 3.236686

50-59#Poor glycemic control | .4908538 .212539 -1.64 0.100 .2100793 1.146888

60-69#Good glycemic control | .6920849 .4187056 -0.61 0.543 .2114412 2.265318

60-69#Poor glycemic control | .2136246 .0923163 -3.57 0.000 .0915821 .4983011

≥70#Good glycemic control | .6083179 .3683955 -0.82 0.412 .1856289 1.993498

≥70#Poor glycemic control | .1418421 .0615486 -4.50 0.000 .0605967 .3320178

|

Kon |

Male | 1 (base)

Female | .7940541 .0193155 -9.48 0.000 .7570848 .8328287

|

Education |

Up to Lower secondary education | 1.191304 .0318404 6.55 0.000 1.130505 1.255374

Upper secondary to Post-secondary education <2 years | 1 (base)

Post-secondary ≥2 years to Tertiary education | .65336 .0239938 -11.59 0.000 .6079856 .7021207

|

maximum_AT | .9123714 .0012799 -65.37 0.000 .9098662 .9148835

No_5p_rank | 1.071333 .0052444 14.08 0.000 1.061104 1.081662

years_followup | 1.225355 .0045031 55.30 0.000 1.216561 1.234213

_cons | .0327817 .0106056 -10.56 0.000 .0173879 .0618037

-----------------------------------------------------------------------------------------------------------------------

Note: _cons estimates baseline relative risk for each outcome.

### Figure A17. Probability estimates for tooth loss (based on multinomial logistic regression, stratified by gender) in T2D with good/poor glycemic control and matched controls without diabetes, by age category.

### Figure A18. Estimated annual incidence rate of tooth loss (based on Poisson regression, stratified by gender) in T2D with good/poor glycemic control and matched controls without diabetes, females and males by age category.

# Diabetes-related complications in T1D

## Table A13. Diabetes-related complications in individuals with Type 1 Diabetes (comparing periodontitis to no periodontitis).

|  |  | **Retinopathy (2010-2020)** | | | **Albuminuria (2010-2020)** | | | **Ischemic heart disease (2010-2020)** | | | **Stroke (2010-2020)** | | | **Mortality (2010-2020)** | | |
| --- | --- | --- | --- | --- | --- | --- | --- | --- | --- | --- | --- | --- | --- | --- | --- | --- |
| Age  Gender | | No periodontitis | Periodontitis | Crude RR (95%CI) | No periodontitis | Periodontitis | Crude RR (95%CI) | No periodontitis | Periodontitis | Crude RR  (95%CI) | No periodontitis | Periodontitis | Crude RR  (95%CI) | No periodontitis | Periodontitis | Crude RR  (95%CI) |
| 18-29 | Female | 2 471 / 3 325 (74.3%) | 148 / 181  (81.8%) | **1.10**  **(1.02, 1.18)** | 584 / 3 328  (17.5%) | 47 / 179  (26.3%) | **1.50**  **(1.16, 1.93)** | 10 / 3 402  (0.3%) | 1 / 183  (0.5%) | 1.86  (0.24, 14.44) | 15 / 3 402  (0.4%) | 1 / 183  (0.5%) | 1.24  (0.16, 9.33) | 19 / 3 402  (0.6%) | 2 / 183  (1.1%) | 1.96  (0.46, 8.34) |
|  | Male | 2 871 / 4 093 (70.1%) | 165 / 223  (74.0%) | 1.05  (0.97, 1.14) | 551 / 4 098  (13.4%) | 44 / 224  (19.6%) | **1.46**  **(1.11, 1.93)** | 12 / 4 192  (0.3%) | 1 / 232  (0.4%) | 1.51  (0.20, 11.53) | 6 / 4 192  (0.1%) | 2 / 232  (0.9%) | **6.02**  **(1.22, 29.68)** | 48 / 4 192  (1.1%) | 1 / 232  (0.4%) | 0.38  (0.05, 2.72) |
|  | Total | 5 342 / 7 418 (72.0%) | 313 / 404  (77.5%) | **1.08**  **(1.02, 1.14)** | 1 135 / 7 426  (15.3%) | 91 / 403  (22.6%) | **1.48**  **(1.22, 1.78)** | 22 / 7 594  (0.3%) | 2 / 415  (0.5%) | 1.66  (0.39, 7.05) | 21 / 7 594  (0.3%) | 3 / 415  (0.7%) | 2.61  (0.78, 8.73) | 67 / 7 594  (0.9%) | 3 / 415  (0.7%) | 0.82  (0.26, 2.59) |
| 30-39 | Female | 1 653 / 2 076  (79.6%) | 134 / 176  (76.1%) | 0.96  (0.88, 1.04) | 441 / 2 074  (21.3%) | 52 / 178  (29.2%) | **1.37**  **(1.08, 1.75)** | 40 / 2 100  (1.9%) | 6 / 181  (3.3%) | 1.74  (0.75, 4.05) | 25 / 2 100  (1.2%) | 4 / 181  (2.2%) | 1.86  (0.65, 5.28) | 28 / 2 100  (1.3%) | 3 / 181  (1.7%) | 1.24  (0.38, 4.05) |
|  | Male | 1 958 / 2 452  (79.9%) | 232 / 280  (82.9%) | 1.04  (0.98, 1.10) | 521 / 2 457  (21.2%) | 80 / 281  (28.5%) | **1.34**  **(1.10, 1.64)** | 42 / 2 495  (1.7%) | 7 / 284  (2.5%) | 1.46  (0.66, 3.23) | 32 / 2 495  (1.3%) | 4 / 284  (1.4%) | 1.10  (0.39, 3.08) | 35 / 2 495  (1.4%) | 3 / 284  (1.1%) | 0.75  (0.23, 2.43) |
|  | Total | 3 611 / 4 528 (79.7%) | 366 / 456  (80.3%) | 1.01  (0.96, 1.06) | 962 / 4 531  (21.2%) | 132 / 459  (28.8%) | **1.35**  **(1.16, 1.58)** | 82 / 4 595  (1.8%) | 13 / 465  (2.8%) | 1.57  (0.88, 2.79) | 57 / 4 595  (1.2%) | 8 / 465  (1.7%) | 1.39  (0.67, 2.89) | 63 / 4 595  (1.4%) | 6 / 465  (1.3%) | 0.94  (0.41, 2.16) |
| 40-49 | Female | 1 740 / 2 129  (81.7%) | 251 / 313  (80.2%) | 0.98  (0.93, 1.04) | 576 / 2 130  (27.0%) | 110 / 313  (35.1%) | **1.30**  **(1.10, 1.53)** | 168 / 2 164  (7.8%) | 24 / 314  (7.6%) | 0.98  (0.65, 1.48) | 55 / 2 164  (2.5%) | 12 / 314  (3.8%) | 1.50  (0.81, 2.78) | 59 / 2 164  (2.7%) | 8 / 314  (2.5%) | 0.93  (0.45, 1.94) |
|  | Male | 2 195 / 2 649  (82.9%) | 393 / 468  (84.0%) | 1.01  (0.97, 1.06) | 777 / 2 652  (29.3%) | 166 / 470  (35.3%) | **1.21**  **(1.05, 1.38)** | 177 / 2 691  (6.6%) | 45 / 476  (9.5%) | **1.44**  **(1.05, 1.96)** | 83 / 2 691  (3.1%) | 16 / 476  (3.4%) | 1.09  (0.64, 1.84) | 104 / 2 691  (3.9%) | 16 / 476  (3.4%) | 0.87  (0.52, 1.46) |
|  | Total | 3 935 / 4 778  (82.4%) | 644 / 781  (82.5%) | 1.00  (0.97, 1.04) | 1 353 / 4 782  (28.3%) | 276 / 783  (35.2%) | **1.25**  **(1.12, 1.38)** | 345 / 4 855  (7.1%) | 69 / 790  (8.7%) | 1.23  (0.96, 1.57) | 138 / 4 855  (2.8%) | 28 / 790  (3.5%) | 1.25  (0.84, 1.86) | 163 / 4 855  (3.4%) | 24 / 790  (3.0%) | 0.90  (0.59, 1.38) |
| 50-59 | Female | 1 334 / 1 672  (79.8%) | 307 / 352  (87.2%) | **1.09**  **(1.04, 1.15)** | 546 / 1 669  (32.7%) | 116 / 352  (33.0%) | 1.01  (0.85, 1.19) | 260 / 1 693  (15.4%) | 49 / 357  (13.7%) | 0.89  (0.67, 1.19) | 74 / 1 693  (4.4%) | 14 / 357  (3.9%) | 0.90  (0.51, 1.57) | 92 / 1 693  (5.4%) | 23 / 357  (6.4%) | 1.19  (0.76, 1.85) |
|  | Male | 1 642 / 1 941  (84.6%) | 459 / 516  (89.0%) | **1.05**  **(1.01, 1.09)** | 734 / 1 948  (37.7%) | 224 / 518  (43.2%) | **1.15**  **(1.02, 1.29)** | 370 / 1 980  (18.7%) | 92 / 523  (17.6%) | 0.94  (0.77, 1.16) | 110 / 1 980  (5.6%) | 29 / 523  (5.5%) | 1.00  (0.67, 1.49) | 159 / 1 980  (8.0%) | 52 / 523  (9.9%) | 1.24  (0.92, 1.67) |
|  | Total | 2 976 / 3 613 (82.4%) | 766 / 868  (88.2%) | **1.07**  **(1.04, 1.10)** | 1 280 / 3 617  (35.4%) | 340 / 870  (39.1%) | **1.10**  **(1.01, 1.21)** | 630 / 3 673  (17.2%) | 141 / 880  (16.0%) | 0.93  (0.79, 1.10) | 184 / 3 673  (5.0%) | 43 / 880  (4.9%) | 0.98  (0.71, 1.35) | 251 / 3 673  (6.8%) | 75 / 880  (8.5%) | 1.25  (0.97, 1.60) |
| 60-69 | Female | 1 045 / 1 256  (83.2%) | 237 / 286  (82.9%) | 1.00  (0.94, 1.06) | 469 / 1 266  (37.0%) | 95 / 286  (33.2%) | 0.90  (0.75, 1.07) | 261 / 1 292  (20.2%) | 48 / 289  (16.6%) | 0.82  (0.62, 1.09) | 73 / 1 292  (5.7%) | 19 / 289  (6.6%) | 1.16  (0.71, 1.90) | 194 / 1 292  (15.0%) | 42 / 289  (14.5%) | 0.97  (0.71, 1.32) |
|  | Male | 1 228 / 1 412  (87.0%) | 388 / 456  (85.1%) | 0.98  (0.94, 1.02) | 733 / 1 431  (51.2%) | 219 / 462  (47.4%) | 0.93  (0.83, 1.03) | 433 / 1 456  (29.7%) | 120 / 468  (25.6%) | 0.86  (0.73, 1.03) | 132 / 1 456  (9.1%) | 40 / 468  (8.5%) | 0.94  (0.67, 1.32) | 272 / 1 456  (18.7%) | 75 / 468  (16.0%) | 0.86  (0.68, 1.08) |
|  | Total | 2 273 / 2 668  (85.2%) | 625 / 742  (84.2%) | 0.99  (0.95, 1.02) | 1 202 / 2 697  (44.6%) | 314 / 748  (42.0%) | 0.94  (0.86, 1.03) | 694 / 2 748  (25.3%) | 168 / 757  (22.2%) | 0.88  (0.76, 1.02) | 205 / 2 748  (7.5%) | 59 / 757  (7.8%) | 1.04  (0.79, 1.38) | 466 / 2 748  (17.0%) | 117 / 757  (15.5%) | 0.91  (0.76, 1.10) |
| ≥70 | Female | 528 / 643  (82.1%) | 102 / 127  (80.3%) | 0.98  (0.89, 1.07) | 319 / 658  (48.5%) | 55 / 128  (43.0%) | 0.89  (0.72, 1.10) | 241 / 715  (33.7%) | 42 / 131  (32.1%) | 0.95  (0.73, 1.25) | 103 / 715  (14.4%) | 14 / 131  (10.7%) | 0.74  (0.44, 1.26) | 317 / 715  (44.3%) | 51 / 131  (38.9%) | 0.88  (0.70, 1.10) |
|  | Male | 453 / 561  (80.7%) | 107 / 128  (83.6%) | 1.04  (0.95, 1.13) | 341 / 580  (58.8%) | 80 / 130  (61.5%) | 1.05  (0.90, 1.22) | 249 / 627  (39.7%) | 55 / 137  (40.1%) | 1.01  (0.81, 1.27) | 94 / 627  (15.0%) | 26 / 137  (19.0%) | 1.27  (0.85, 1.88) | 321 / 627  (51.2%) | 67 / 137  (48.9%) | 0.96  (0.79, 1.15) |
|  | Total | 981 / 1 204  (81.5%) | 209 / 255  (82.0%) | 1.01  (0.94, 1.07) | 660 / 1 238  (53.3%) | 135 / 258  (52.3%) | 0.98  (0.86, 1.12) | 490 / 1 342  (36.5%) | 97 / 268  (36.2%) | 0.99  (0.83, 1.18) | 197 / 1 342  (14.7%) | 40 / 268  (14.9%) | 1.02  (0.74, 1.39) | 638 / 1 342  (47.5%) | 118 / 268  (44.0%) | 0.93  (0.80, 1.07) |
| Overall | Female | 8 771 / 11 101  (79.0%) | 1 179 / 1 435  (82.2%) | **1.04**  **(1.01, 1.07)** | 2 935 / 11 125  (26.4%) | 475 / 1 436  (33.1%) | **1.25**  **(1.16, 1.36)** | 980 / 11 366  (8.6%) | 170 / 1 455  (11.7%) | **1.36**  **(1.16, 1.58)** | 345 / 11 366  (3.0%) | 64 / 1 455  (4.4%) | **1.45**  **(1.12, 1.88)** | 709 / 11 366  (6.2%) | 129 / 1 455  (8.9%) | **1.42**  **(1.19, 1.70)** |
|  | Male | 10 347 / 13 108  (78.9%) | 1 744 / 2 071  (84.2%) | **1.07**  **(1.05, 1.09)** | 3 657 / 13 166  (27.8%) | 813 / 2 085  (39.0%) | **1.40**  **(1.32, 1.49)** | 1283 / 13 441  (9.5%) | 320 / 2 120  (15.1%) | **1.58**  **(1.41, 1.77)** | 457 / 13 441  (3.4%) | 117 / 2 120  (5.5%) | **1.62**  **(1.33, 1.98)** | 939 / 13 441  (7.0%) | 214 / 2 120  (10.1%) | **1.44**  **(1.25, 1.66)** |
|  | Total | 19 118 / 24 209  (79.0%) | 2 923 / 3 506  (83.4%) | **1.06**  **(1.04, 1.07)** | 6 592 / 24 291  (27.1%) | 1288 / 3 521  (36.6%) | **1.35**  **(1.28,1.41)** | 2263 / 24 807  (9.1%) | 490 / 3 575  (13.7%) | **1.50**  **(1.37, 1.65)** | 802 / 24 807  (3.2%) | 181 / 3 575  (5.1%) | **1.57**  **(1.34, 1.83)** | 1 648 / 24 807  (6.6%) | 343 / 3 575  (9.6%) | **1.44**  **(1.29, 1.61)** |
| Adjusted HR* (95%CI) | | | | **1.08**  **(1.02, 1.14)** | Adjusted HR* (95%CI) | | **1.14**  **(1.06, 1.23)** | Adjusted HR* (95%CI) | | 0.96  (0.86, 1.08) | Adjusted HR* (95%CI) | | 1.05  (0.89, 1.25) | Adjusted HR* (95%CI) | | 0.91  (0.81, 1.02) |
| Prevalence is presented as frequency / n (%)  *Adjusted HRs originate from cox regression models, which included periodontitis, age category, gender, level of education and number of years in the 5Th lowest percentile of income.  Statistically significant crude RRs and adjusted HRs are highlighted in **bold** | | | | | | | | | | | | | | | | |

## Retinopathy

### Figure A19. Prevalence of retinopathy (2010-2020) in T1D with and without periodontitis, females and males by age category (excluding individuals deceased over the observation period).

### Cox regression model T1D with and without periodontitis (outcome: retinopathy)

Cox regression with Breslow method for ties

No. of subjects = 17,788 Number of obs = 17,788

No. of failures = 12,239

Time at risk = 88,930

LR chi2(10) = 90.05

Log likelihood = -113562.24 Prob > chi2 = 0.0000

---------------------------------------------------------------------------------------------------------

_t | Haz. ratio Std. err. z P>|z| [95% conf. interval]

----------------------------------------+----------------------------------------------------------------

everParod2 |

Yes | 1.077569 .030458 2.64 0.008 1.019496 1.13895

|

cohort |

30-39 | 1.119987 .0295901 4.29 0.000 1.063467 1.17951

40-49 | 1.136841 .0299639 4.87 0.000 1.079604 1.197113

50-59 | 1.147288 .0336357 4.69 0.000 1.083222 1.215144

60-69 | 1.180817 .0390356 5.03 0.000 1.106735 1.259858

≥70 | 1.198709 .0568038 3.82 0.000 1.09239 1.315377

|

Kon |

Female | .9894082 .0181565 -0.58 0.562 .9544545 1.025642

|

Education |

Up to Lower secondary education | .981754 .0286358 -0.63 0.528 .927203 1.039515

Post-secondary ≥2 years to Tertiary .. | .9400824 .0189617 -3.06 0.002 .9036431 .977991

|

No_5p_rank | 1.012706 .0047084 2.72 0.007 1.003519 1.021976

---------------------------------------------------------------------------------------------------------

### Figure A20. Retinopathy in T1D with and without periodontitis. Cohort-adjusted rates by age and IRR for females and males, based on age-period-cohort models (truncated at 30-60 years and period 2011-2019).

## Albuminuria

### Figure A21. Prevalence of albuminuria (2010-2020) in T1D with and without periodontitis, females and males by age category (excluding individuals deceased over the observation period).

### Cox regression model T1D with and without periodontitis (outcome: albuminuria)

Cox regression with Breslow method for ties

No. of subjects = 24,817 Number of obs = 24,817

No. of failures = 5,083

Time at risk = 204,357

LR chi2(10) = 1171.81

Log likelihood = -50053.262 Prob > chi2 = 0.0000

---------------------------------------------------------------------------------------------------------

_t | Haz. ratio Std. err. z P>|z| [95% conf. interval]

----------------------------------------+----------------------------------------------------------------

everParod2 |

Yes | 1.141491 .0441582 3.42 0.001 1.058142 1.231405

|

cohort |

30-39 | 1.202359 .0598248 3.70 0.000 1.090641 1.325521

40-49 | 1.529729 .0697801 9.32 0.000 1.398898 1.672796

50-59 | 1.91287 .0887314 13.98 0.000 1.746631 2.094931

60-69 | 2.608472 .1230409 20.33 0.000 2.378128 2.861127

≥70 | 3.641806 .208591 22.57 0.000 3.255088 4.074468

|

Kon |

Female | .9505759 .027122 -1.78 0.076 .8988768 1.005248

|

Education |

Up to Lower secondary education | 1.193817 .0459271 4.60 0.000 1.107112 1.287313

Post-secondary ≥2 years to Tertiary .. | .7503793 .0257584 -8.37 0.000 .7015546 .802602

|

No_5p_rank | 1.045092 .0065476 7.04 0.000 1.032337 1.058004

---------------------------------------------------------------------------------------------------------

### Figure A22. Albuminuria in T1D with and without periodontitis. Cohort-adjusted rates by age and IRR for females and males, based on age-period-cohort models (truncated at 30-60 years and period 2011-2019).

## Ischemic heart disease

### Figure A23. Prevalence of ischemic heart disease (2010-2020) in T1D with and without periodontitis, females and males by age category (excluding individuals deceased over the observation period).

### Cox regression model T1D with and without periodontitis (outcome: ischemic heart disease)

Cox regression with Breslow method for ties

No. of subjects = 27,290 Number of obs = 27,290

No. of failures = 1,982

Time at risk = 243,289

LR chi2(10) = 2573.16

Log likelihood = -18756.165 Prob > chi2 = 0.0000

---------------------------------------------------------------------------------------------------------

_t | Haz. ratio Std. err. z P>|z| [95% conf. interval]

----------------------------------------+----------------------------------------------------------------

everParod2 |

Yes | .9588335 .056007 -0.72 0.472 .8551122 1.075136

|

cohort |

30-39 | 5.78838 1.390131 7.31 0.000 3.615209 9.267885

40-49 | 20.77068 4.575729 13.77 0.000 13.48755 31.98661

50-59 | 46.07698 10.0328 17.59 0.000 30.0707 70.60323

60-69 | 68.86273 14.99341 19.44 0.000 44.94206 105.5153

≥70 | 114.1858 25.20605 21.46 0.000 74.08201 175.9994

|

Kon |

Female | .8268193 .0380352 -4.13 0.000 .7555335 .904831

|

Education |

Up to Lower secondary education | 1.186337 .065407 3.10 0.002 1.064825 1.321715

Post-secondary ≥2 years to Tertiary .. | .7924792 .0469291 -3.93 0.000 .705637 .890009

|

No_5p_rank | 1.028323 .0109145 2.63 0.009 1.007152 1.049939

---------------------------------------------------------------------------------------------------------

### Figure A24. Ischemic heart disease in T1D with and without periodontitis. Cohort-adjusted rates by age and IRR for females and males, based on age-period-cohort models (truncated at 30-60 years and period 2011-2019).

## Stroke

### Figure A25. Prevalence of stroke (2010-2020) in T1D with and without periodontitis, females and males by age category (excluding individuals deceased over the observation period).

### Cox regression model T1D with and without periodontitis (outcome: stroke)

Cox regression with Breslow method for ties

No. of subjects = 27,938 Number of obs = 27,938

No. of failures = 873

Time at risk = 253,965

LR chi2(10) = 968.82

Log likelihood = -8365.7897 Prob > chi2 = 0.0000

---------------------------------------------------------------------------------------------------------

_t | Haz. ratio Std. err. z P>|z| [95% conf. interval]

----------------------------------------+----------------------------------------------------------------

everParod2 |

Yes | 1.051505 .0916025 0.58 0.564 .8864582 1.247281

|

cohort |

30-39 | 4.373842 1.111817 5.81 0.000 2.657604 7.1984

40-49 | 9.557269 2.227679 9.68 0.000 6.052425 15.0917

50-59 | 15.52832 3.576795 11.91 0.000 9.886865 24.38879

60-69 | 24.99873 5.729264 14.04 0.000 15.95283 39.17402

≥70 | 57.88885 13.41648 17.51 0.000 36.75514 91.17415

|

Kon |

Female | .7738809 .0540874 -3.67 0.000 .6748119 .8874942

|

Education |

Up to Lower secondary education | 1.070656 .0894718 0.82 0.414 .9089027 1.261196

Post-secondary ≥2 years to Tertiary .. | .7193453 .0654684 -3.62 0.000 .6018229 .8598172

|

No_5p_rank | 1.060291 .0144745 4.29 0.000 1.032298 1.089044

---------------------------------------------------------------------------------------------------------

### Figure A26. Stroke in T1D with and without periodontitis. Cohort-adjusted rates by age and IRR for females and males, based on age-period-cohort models (truncated at 30-60 years and period 2011-2019).

## Mortality

### Figure A27. Mortality (2010-2020) in T1D with and without periodontitis, females and males by age category.

### Cox regression model T1D with and without periodontitis (outcome: death)

Cox regression with Breslow method for ties

No. of subjects = 28,041 Number of obs = 28,041

No. of failures = 1,942

Time at risk = 257,631

LR chi2(10) = 3451.87

Log likelihood = -17965.405 Prob > chi2 = 0.0000

---------------------------------------------------------------------------------------------------------

_t | Haz. ratio Std. err. z P>|z| [95% conf. interval]

----------------------------------------+----------------------------------------------------------------

everParod2 |

Yes | .9101192 .0547377 -1.57 0.117 .8089172 1.023982

|

cohort |

30-39 | 1.600409 .2777436 2.71 0.007 1.138961 2.248811

40-49 | 3.559205 .5146056 8.78 0.000 2.680912 4.725236

50-59 | 7.596664 1.038113 14.84 0.000 5.811698 9.929853

60-69 | 17.66509 2.333664 21.74 0.000 13.63537 22.88572

≥70 | 57.19521 7.530414 30.73 0.000 44.18644 74.03383

|

Kon |

Female | .7956467 .0371664 -4.89 0.000 .7260371 .8719303

|

Education |

Up to Lower secondary education | 1.290286 .0670712 4.90 0.000 1.165304 1.428673

Post-secondary ≥2 years to Tertiary .. | .5818712 .0398608 -7.90 0.000 .5087633 .6654846

|

No_5p_rank | 1.024102 .0105159 2.32 0.020 1.003697 1.044921

---------------------------------------------------------------------------------------------------------

### Figure A28. Mortality in T1D with and without periodontitis. Cohort-adjusted rates by age and IRR for females and males, based on age-period-cohort models (truncated at 50-70 years and period 2011-2019) (left). Main cause of mortality in T1D, females and males (right).

# Diabetes-related complications in T2D

## Table A14. Diabetes-related complications in individuals with Type 2 Diabetes (comparing periodontitis to no periodontitis).

|  |  | **Retinopathy (2010-2020)** | | | **Albuminuria (2010-2020)** | | | **Ischemic heart disease (2010-2020)** | | | **Stroke (2010-2020)** | | | **Mortality (2010-2020)** | | |
| --- | --- | --- | --- | --- | --- | --- | --- | --- | --- | --- | --- | --- | --- | --- | --- | --- |
| Age  Gender | | No periodontitis | Periodontitis | Crude RR (95%CI) | No periodontitis | Periodontitis | Crude RR (95%CI) | No periodontitis | Periodontitis | Crude RR (95%CI) | No periodontitis | Periodontitis | Crude RR (95%CI) | No periodontitis | Periodontitis | Crude RR (95%CI) |
| 18-29 | Female | 309 / 1 369  (22.6%) | 31 / 141  (22.0%) | 0.97  (0.70, 1.35) | 396 / 1 541  (25.7%) | 39 / 166  (23.5%) | 0.91  (0.69, 1.22) | 11 / 1 892  (0.6%) | 3 / 185  (1.6%) | 2.79  (0.79, 9.91) | 10 / 1 892  (0.5%) | 2 / 185  (1.1%) | 2.05  (0.45, 9.27) | 11 / 1 892  (0.6%) | 2 / 185  (1.1%) | 1.86  (0.42, 8.33) |
|  | Male | 379 / 1 379  (27.5%) | 58 / 158  (36.7%) | **1.34**  **(1.07, 1.67)** | 427 / 1 555  (27.5%) | 50 / 182  (27.5%) | 1.00  (0.78, 1.28) | 15 / 1 865  (0.8%) | 3 / 205  (1.5%) | 1.82  (0.53, 6.23) | 13 / 1 865  (0.7%) | 0 / 205  (0.0%) | - | 18 / 1 865  (1.0%) | 4 / 205  (2.0%) | 2.02  (0.69, 5.92) |
|  | Total | 688 / 2 748  (25.0%) | 89 / 299  (29.8%) | 1.19  (0.99, 1.43) | 823 / 3 096  (26.6%) | 89 / 348  (25.6%) | 0.96  (0.80, 1.16) | 26 / 3 757  (0.7%) | 6 / 390  (1.5%) | 2.22  (0.92, 5.37) | 23 / 3 757  (0.6%) | 2 / 390  (0.5%) | 0.84  (0.20, 3.54) | 29 / 3 757  (0.8%) | 6 / 390  (1.5%) | 1.99  (0.83, 4.77) |
| 30-39 | Female | 927 / 3 435  (27.0%) | 202 / 652  (31.0%) | **1.15**  **(1.01, 1.30)** | 1 000 / 3 776  (26.5%) | 230 / 689  (33.4%) | **1.26**  **(1.12, 1.42)** | 78 / 4 341  (1.8%) | 18 / 790  (2.3%) | 1.27  (0.76, 2.11) | 52 / 4 341  (1.2%) | 9 / 790  (1.1%) | 0.95  (0.47, 1.92) | 47 / 4 341  (1.1%) | 11 / 790  (1.4%) | 1.29  (0.67, 2.47) |
|  | Male | 1 333 / 4 085  (32.6%) | 349 / 909  (38.4%) | **1.18**  **(1.07, 1.29)** | 1 207 / 4 424  (27.3%) | 315 / 1 029  (30.6%) | **1.12**  **(1.01, 1.24)** | 206 / 5 022  (4.1%) | 73 / 1 140  (6.4%) | **1.56**  **(1.20, 2.02)** | 79 / 5 022  (1.6%) | 14 / 1140  (1.2%) | 0.78  (0.44, 1.37) | 68 / 5 022  (1.4%) | 8 / 1140  (0.7%) | 0.52  (0.25, 1.08) |
|  | Total | 2 260 / 7 520  (30.1%) | 551 / 1 561  (35.3%) | **1.17**  **(1.09, 1.27)** | 2 207 / 8 200  (26.9%) | 545 / 1 718  (31.7%) | **1.18**  **(1.09, 1.27)** | 284 / 9 363  (3.0%) | 91 / 1 930  (4.7%) | **1.55**  **(1.23, 1.96)** | 131 / 9 363  (1.4%) | 23 / 1 930  (1.2%) | 0.85  (0.55, 1.32) | 115 / 9 363  (1.2%) | 19 / 1 930  (1.0%) | 0.80  (0.49, 1.30) |
| 40-49 | Female | 2 792 / 9 232  (30.2%) | 759 / 2 158  (35.2%) | **1.16**  **(1.09, 1.24)** | 2 725 / 9 830  (27.7%) | 769 / 2 292  (33.6%) | **1.21**  **(1.13, 1.29)** | 563 / 11 007  (5.1%) | 164 / 2 506  (6.5%) | **1.28**  **(1.08, 1.51)** | 284 / 11 007  (2.6%) | 63 / 2 506  (2.5%) | 0.97  (0.74, 1.28) | 164 / 11 007  (1.5%) | 36 / 2 506  (1.4%) | 0.96  (0.67, 1.38) |
|  | Male | 4 523 / 12 780  (35.4%) | 1 513 / 3 792  (39.9%) | **1.13**  **(1.08, 1.18)** | 4 170 / 13 548  (30.8%) | 1 369 / 4 004  (34.2%) | **1.11**  **(1.06, 1.17)** | 1 442 / 14 960  (9.6%) | 511 / 4 378  (11.7%) | **1.21**  **(1.10, 1.33)** | 394 / 14 960  (2.6%) | 119 / 4 378  (2.7%) | 1.03  (0.84, 1.26) | 352 / 14 960  (2.4%) | 98 / 4 378  (2.2%) | 0.95  (0.76, 1.19) |
|  | Total | 7 315 / 22 012  (33.2%) | 2 272 / 5 950  (38.2%) | **1.15**  **(1.11, 1.19)** | 6 895 / 23 378  (29.5%) | 2 138 / 6 296  (34.0%) | **1.15**  **(1.11, 1.20)** | 2 005 / 25 967  (7.7%) | 675 / 6 884  (9.8%) | **1.27**  **(1.17, 1.38)** | 678 / 25 967  (2.6%) | 182 / 6 884  (2.6%) | 1.01  (0.86, 1.19) | 516 / 25 967  (2.0%) | 134 / 6 884  (1.9%) | 0.98  (0.81, 1.18) |
| 50-59 | Female | 5 313 / 15 659  (33.9%) | 1 592 / 4 219  (37.7%) | **1.11**  **(1.06, 1.16)** | 4 652 / 16 374  (28.4%) | 1 546 / 4 478  (34.5%) | **1.22**  **(1.16, 1.27)** | 1 761 / 17 985  (9.8%) | 512 / 4 859  (10.5%) | 1.08  (0.98, 1.18) | 635 / 17 985  (3.5%) | 194 / 4 859  (4.0%) | 1.13  (0.97, 1.32) | 683 / 17 985  (3.8%) | 185 / 4 859  (3.8%) | 1.00  (0.85, 1.18) |
|  | Male | 8 207 / 22 000  (37.3%) | 3 335 / 8 002  (41.7%) | **1.12**  **(1.08, 1.15)** | 8141 / 22896  (35.6%) | 3 306 / 8 348  (39.6%) | **1.11**  **(1.08, 1.15)** | 4 562 / 24 947  (18.3%) | 1 839 / 8 992  (20.5%) | **1.12**  **(1.07, 1.17)** | 1 217 / 24 947  (4.9%) | 475 / 8 992  (5.3%) | 1.08  (0.98, 1.20) | 1 284 / 24 947  (5.1%) | 434 / 8 992  (4.8%) | 0.94  (0.84, 1.04) |
|  | Total | 13 520 / 37 659  (35.9%) | 4 927 / 12 221  (40.3%) | **1.12**  **(1.09, 1.15)** | 12 793 / 39 270  (32.6%) | 4 852 / 12 826  (37.8%) | **1.16**  **(1.13, 1.19)** | 6 323 / 42 932  (14.7%) | 2 351 / 13 851  (17.0%) | **1.15**  **(1.10, 1.20)** | 1 852 / 42 932  (4.3%) | 669 / 13 851  (4.8%) | **1.12**  **(1.03, 1.22)** | 1 967 / 42 932  (4.6%) | 619 / 13 851  (4.5%) | 0.98  (0.89, 1.07) |
| 60-69 | Female | 8 927 / 23 420  (38.1%) | 2 347 / 5 831  (40.3%) | **1.06**  **(1.02, 1.09)** | 8 340 / 24 287  (34.3%) | 2 313 / 6 086  (38.0%) | **1.11**  **(1.07, 1.15)** | 4 147 / 26 400  (15.7%) | 977 / 6 569  (14.9%) | 0.95  (0.89, 1.01) | 1 709 / 26 400  (6.5%) | 453 / 6 569  (6.9%) | 1.07  (0.96, 1.18) | 2 377 / 26 400  (9.0%) | 557 / 6 569  (8.5%) | 0.94  (0.86, 1.03) |
|  | Male | 12 709 / 29 781  (42.7%) | 5 044 / 11 374  (44.3%) | **1.04**  **(1.01, 1.06)** | 13 669 / 30 740  (44.5%) | 5 546/ 11 774  (47.1%) | **1.06**  **(1.04, 1.08)** | 9 297 / 33 234  (28.0%) | 3 527 / 12 613  (28.0%) | 1.00  (0.97, 1.03) | 2 914 / 33 234  (8.8%) | 1 144 / 12 613  (9.1%) | 1.03  (0.97, 1.10) | 3 906 / 33 234  (11.8%) | 1 432 / 12 613  (11.4%) | 0.97  (0.91, 1.02) |
|  | Total | 21 636 / 53 201  (40.7%) | 7 391 / 17 205  (43.0%) | **1.06**  **(1.04, 1.08)** | 22 009 / 55 027  (40.0%) | 7 859 / 17 860  (44.0%) | **1.10**  **(1.08, 1.12)** | 13 444 / 59 634  (22.5%) | 4 504 / 19 182  (23.5%) | **1.04**  **(1.01, 1.07)** | 4 623 / 59 634  (7.8%) | 1 597 / 19 182  (8.3%) | **1.07**  **(1.02, 1.13)** | 6 283 / 59 634  (10.5%) | 1 989 / 19 182  (10.4%) | 0.98  (0.94, 1.03) |
| ≥70 | Female | 9 236 / 21 798  (42.4%) | 1 730 / 3 952  (43.8%) | 1.03  (0.99, 1.07) | 10 293 / 23 112  (44.5%) | 1 914 / 4 185  (45.7%) | 1.03  (0.99, 1.06) | 7 551 / 27 068  (27.9%) | 1 141 / 4 718  (24.2%) | **0.87**  **(0.82, 0.92)** | 3 648 / 27 068  (13.5%) | 643 / 4 718  (13.6%) | 1.01  (0.94, 1.09) | 10 079 / 27 068  (37.2%) | 1 389 / 4 718  (29.4%) | **0.79**  **(0.75, 0.83)** |
|  | Male | 8 801 / 20 185  (43.6%) | 2 527 / 5 486  (46.1%) | **1.06**  **(1.02, 1.09)** | 11 919 / 21 379  (55.8%) | 3 300 / 5 788  (57.0%) | 1.02  (1.00, 1.05) | 10 030 / 24 451  (41.0%) | 2 413 / 6 362  (37.9%) | **0.92**  **(0.89, 0.96)** | 3 832 / 24 451  (15.7%) | 982 / 6 362  (15.4%) | 0.98  (0.92, 1.05) | 10 538 / 24 451  (43.1%) | 2 217 / 6 362  (34.8%) | **0.81**  **(0.78, 0.84)** |
|  | Total | 18 037 / 41 983  (43.0%) | 4 257 / 9438  (45.1%) | **1.05**  **(1.02, 1.08)** | 22 212 / 44 491  (49.9%) | 5 214 / 9 973  (52.3%) | **1.05**  **(1.03, 1.07)** | 17 581 / 51 519  (34.1%) | 3 554 / 11 080  (32.1%) | **0.94**  **(0.91, 0.97)** | 7 480 / 51 519  (14.5%) | 1 625 / 11 080  (14.7%) | 1.01  (0.96, 1.06) | 20 617 / 51 519  (40.0%) | 3 606 / 11 080  (32.5%) | **0.81**  **(0.79, 0.84)** |
| Overall | Female | 27 504 / 74 913  (36.7%) | 6 661 / 16 953  (39.3%) | **1.07**  **(1.05, 1.09)** | 27 406 / 78 920  (34.7%) | 6 811 / 17 896  (38.1%) | **1.10**  **(1.07, 1.12)** | 14 111 / 88 693  (15.9%) | 2 815 / 19 627  (14.3%) | **0.90**  **(0.87, 0.94)** | 6 338 / 88 693  (7.1%) | 1 364 / 19 627  (6.9%) | 0.97  (0.92, 1.03) | 13 361 / 88 693  (15.1%) | 2 180 / 19 627  (11.1%) | **0.74**  **(0.71, 0.77)** |
|  | Male | 35 952 / 90 210  (39.9%) | 12 826 / 29 721  (43.2%) | **1.08**  **(1.07, 1.10)** | 39 533 / 94 542  (41.8%) | 13 886 / 31 125  (44.6%) | **1.07**  **(1.05, 1.08)** | 25 552 / 104 479  (24.5%) | 8 366 / 33690  (24.8%) | 1.02  (0.99, 1.04) | 8 449 / 104 479  (8.1%) | 2 734 / 33 690  (8.1%) | 1.00  (0.96, 1.05) | 16 166 / 104 479  (15.5%) | 4 193 / 33 690  (12.4%) | **0.80**  **(0.78, 0.83)** |
|  | Total | 63 456 / 165 123  (38.4%) | 19 487 / 46 674  (41.8%) | **1.09**  **(1.07, 1.10)** | 66 939 / 173 462  (38.6%) | 20 697 / 49 021  (42.2%) | **1.09**  **(1.08, 1.11)** | 39 663 / 193 172  (20.5%) | 11 181 / 53317  (21.0%) | 1.02  (1.00, 1.04) | 14 787 / 193 172  (7.7%) | 4 098 / 53 317  (7.7%) | 1.00  (0.97, 1.04) | 29 527 / 193 172  (15.3%) | 6 373 / 53 317  (12.0%) | **0.78**  **(0.76, 0.80)** |
| Adjusted HR* (95%CI) | | | | **1.08**  **(1.06, 1.10)** | Adjusted HR* (95%CI) | | **1.09**  **(1.07, 1.11)** | Adjusted HR* (95%CI) | | **0.96**  **(0.94, 0.99)** | Adjusted HR* (95%CI) | | 0.99  (0.95, 1.03) | Adjusted HR* (95%CI) | | **0.81**  **(0.79, 0.83)** |
| Prevalence is presented as frequency / n (%)  *Adjusted HRs originate from cox regression models, which included periodontitis, age category, gender, level of education and number of years in the 5^th^ lowest percentile of income.  Statistically significant crude RRs and adjusted HRs are highlighted in **bold** | | | | | | | | | | | | | | | | |

## Retinopathy

### Figure A29. Prevalence of retinopathy (2010-2020) in T2D with and without periodontitis, females and males by age category (excluding individuals deceased over the observation period).

### Cox regression model T2D with and without periodontitis (outcome: retinopathy)

Cox regression with Breslow method for ties

No. of subjects = 187,388 Number of obs = 187,388

No. of failures = 63,032

Time at risk = 1,100,711

LR chi2(10) = 441.26

Log likelihood = -739357.21 Prob > chi2 = 0.0000

---------------------------------------------------------------------------------------------------------

_t | Haz. ratio Std. err. z P>|z| [95% conf. interval]

----------------------------------------+----------------------------------------------------------------

everParod2 |

Yes | 1.084212 .0102077 8.59 0.000 1.064389 1.104405

|

cohort |

30-39 | 1.119513 .0505874 2.50 0.012 1.024627 1.223185

40-49 | 1.121695 .0467643 2.75 0.006 1.033683 1.2172

50-59 | 1.106595 .0453456 2.47 0.013 1.021194 1.199137

60-69 | 1.137167 .0463119 3.16 0.002 1.049925 1.231657

≥70 | 1.130179 .0463529 2.98 0.003 1.042884 1.22478

|

Kon |

Female | .8812678 .0071989 -15.47 0.000 .8672706 .8954909

|

Education |

Up to Lower secondary education | 1.070908 .0097521 7.52 0.000 1.051964 1.090194

Post-secondary ≥2 years to Tertiary .. | .9871461 .0111478 -1.15 0.252 .9655369 1.009239

|

No_5p_rank | 1.005081 .0017967 2.83 0.005 1.001565 1.008608

---------------------------------------------------------------------------------------------------------

### Figure A30. Retinopathy in T2D with and without periodontitis. Cohort-adjusted rates by age and IRR for females and males, based on age-period-cohort models (truncated at 30-60 years and period 2011-2019).

## Albuminuria

### Figure A31. Prevalence of albuminuria (2010-2020) in T2D with and without periodontitis, females and males by age category (excluding individuals deceased over the observation period).

### Cox regression model T2D with and without periodontitis (outcome: albuminuria)

Cox regression with Breslow method for ties

No. of subjects = 190,677 Number of obs = 190,677

No. of failures = 62,289

Time at risk = 1,133,619

LR chi2(10) = 2887.58

Log likelihood = -730138.99 Prob > chi2 = 0.0000

---------------------------------------------------------------------------------------------------------

_t | Haz. ratio Std. err. z P>|z| [95% conf. interval]

----------------------------------------+----------------------------------------------------------------

everParod2 |

Yes | 1.088601 .0103184 8.96 0.000 1.068564 1.109014

|

cohort |

30-39 | .946996 .0447136 -1.15 0.249 .8632917 1.038816

40-49 | .9560376 .0410938 -1.05 0.296 .8787947 1.04007

50-59 | 1.01626 .0427423 0.38 0.701 .9358462 1.103583

60-69 | 1.179034 .0491755 3.95 0.000 1.086486 1.279465

≥70 | 1.507396 .0630713 9.81 0.000 1.388711 1.636223

|

Kon |

Female | .7739548 .0064105 -30.94 0.000 .7614919 .7866218

|

Education |

Up to Lower secondary education | 1.057499 .0095914 6.16 0.000 1.038866 1.076465

Post-secondary ≥2 years to Tertiary .. | .9248584 .0107364 -6.73 0.000 .904053 .9461425

|

No_5p_rank | 1.011989 .0017723 6.80 0.000 1.008521 1.015469

---------------------------------------------------------------------------------------------------------

### Figure A32. Albuminuria in T2D with and without periodontitis. Cohort-adjusted rates by age and IRR for females and males, based on age-period-cohort models (truncated at 30-60 years and period 2011-2019).

## Ischemic heart disease

### Figure A33. Prevalence of ischemic heart disease (2010-2020) in T2D with and without periodontitis, females and males by age category (excluding individuals deceased over the observation period).

### Cox regression model T2D with and without periodontitis (outcome: ischemic heart disease)

Cox regression with Breslow method for ties

No. of subjects = 214,426 Number of obs = 214,426

No. of failures = 29,867

Time at risk = 1,430,544

LR chi2(10) = 11318.87

Log likelihood = -351088.21 Prob > chi2 = 0.0000

---------------------------------------------------------------------------------------------------------

_t | Haz. ratio Std. err. z P>|z| [95% conf. interval]

----------------------------------------+----------------------------------------------------------------

everParod2 |

Yes | .9634705 .0135089 -2.65 0.008 .937354 .9903146

|

cohort |

30-39 | 2.808062 .6626881 4.38 0.000 1.768184 4.459497

40-49 | 6.536366 1.472487 8.33 0.000 4.203209 10.16463

50-59 | 12.09876 2.711692 11.12 0.000 7.797605 18.77243

60-69 | 18.91549 4.234728 13.13 0.000 12.19702 29.33468

≥70 | 34.67288 7.761491 15.84 0.000 22.35884 53.76883

|

Kon |

Female | .5665987 .0070064 -45.94 0.000 .5530314 .5804989

|

Education |

Up to Lower secondary education | 1.110698 .0141919 8.22 0.000 1.083228 1.138865

Post-secondary ≥2 years to Tertiary .. | .8973458 .0158973 -6.11 0.000 .8667225 .9290511

|

No_5p_rank | 1.023663 .0025446 9.41 0.000 1.018688 1.028662

---------------------------------------------------------------------------------------------------------

### Figure A34. Ischemic heart disease in T2D with and without periodontitis. Cohort-adjusted rates by age and IRR for females and males, based on age-period-cohort models (truncated at 30-60 years and period 2011-2019).

## Stroke

### Figure A35. Prevalence of stroke (2010-2020) in T2D with and without periodontitis, females and males by age category (excluding individuals deceased over the observation period).

### Cox regression model T2D with and without periodontitis (outcome: stroke)

Cox regression with Breslow method for ties

No. of subjects = 228,866 Number of obs = 228,866

No. of failures = 13,561

Time at risk = 1,597,660

LR chi2(10) = 5844.14

Log likelihood = -159640.54 Prob > chi2 = 0.0000

---------------------------------------------------------------------------------------------------------

_t | Haz. ratio Std. err. z P>|z| [95% conf. interval]

----------------------------------------+----------------------------------------------------------------

everParod2 |

Yes | .9902574 .0207852 -0.47 0.641 .9503459 1.031845

|

cohort |

30-39 | 1.606414 .486334 1.57 0.117 .8874833 2.907733

40-49 | 3.389064 .9525362 4.34 0.000 1.953618 5.879223

50-59 | 5.468745 1.523599 6.10 0.000 3.167685 9.441335

60-69 | 10.21857 2.839374 8.36 0.000 5.927506 17.61605

≥70 | 22.43727 6.231618 11.20 0.000 13.01851 38.67039

|

Kon |

Female | .7849306 .0139436 -13.63 0.000 .7580719 .8127409

|

Education |

Up to Lower secondary education | 1.069953 .0202194 3.58 0.000 1.031049 1.110325

Post-secondary ≥2 years to Tertiary .. | .9186518 .0242805 -3.21 0.001 .8722745 .9674949

|

No_5p_rank | 1.014373 .0037697 3.84 0.000 1.007011 1.021788

---------------------------------------------------------------------------------------------------------

### Figure A36. Stroke in T2D with and without periodontitis. Cohort-adjusted rates by age and IRR for females and males, based on age-period-cohort models (truncated at 30-60 years and period 2011-2019).

## Mortality

### Figure A37. Mortality (2010-2020) in T2D with and without periodontitis, females and males by age category.

### Cox regression model T2D with and without periodontitis (outcome: death)

Cox regression with Breslow method for ties

No. of subjects = 233,478 Number of obs = 233,478

No. of failures = 34,743

Time at risk = 1,661,307

LR chi2(10) = 28931.02

Log likelihood = -400561.99 Prob > chi2 = 0.0000

---------------------------------------------------------------------------------------------------------

_t | Haz. ratio Std. err. z P>|z| [95% conf. interval]

----------------------------------------+----------------------------------------------------------------

everParod2 |

Yes | .811194 .0114349 -14.84 0.000 .7890887 .8339185

|

cohort |

30-39 | 1.240035 .2517562 1.06 0.289 .8329525 1.846069

40-49 | 1.706707 .319157 2.86 0.004 1.182995 2.462266

50-59 | 3.446902 .6332709 6.74 0.000 2.40461 4.94098

60-69 | 6.946668 1.271102 10.59 0.000 4.853149 9.943275

≥70 | 25.79855 4.71542 17.78 0.000 18.03075 36.91276

|

Kon |

Female | .8082466 .0089208 -19.29 0.000 .7909499 .8259215

|

Education |

Up to Lower secondary education | 1.219202 .0141982 17.02 0.000 1.191689 1.24735

Post-secondary ≥2 years to Tertiary .. | .8254675 .0148687 -10.65 0.000 .7968337 .8551302

|

No_5p_rank | .9942444 .0024722 -2.32 0.020 .9894107 .9991017

---------------------------------------------------------------------------------------------------------

### Figure A38. Mortality in T2D with and without periodontitis. Cohort-adjusted rates by age and IRR for females and males, based on age-period-cohort models (truncated at 50-70 years and period 2011-2019) (left). Main cause of mortality in T2D, females and males (right).
