# Supplementary material for: Periodontitis in patients with diabetes and its association with diabetes-related complications. A register-based cohort study
Source: BMJ Open. 2024 Jul 4;14(7):e087557. doi: 10.1136/bmjopen-2024-087557 (PMC11227830; doi:10.1136/bmjopen-2024-087557)
Supplement: Supplementary file 3 [file bmjopen-2024-087557.reviewer_comments.pdf]

PEER REVIEW HISTORY

BMJ Open publishes all reviews undertaken for accepted manuscripts. Reviewers are asked to complete a checklist review form (**http://bmjopen.bmj.com/site/about/resources/checklist.pdf**) and are provided with free text boxes to elaborate on their assessment. These free text comments are reproduced below.

ARTICLE DETAILS

**TITLE (PROVISIONAL)**	Periodontitis in patients with diabetes and its association with diabetes-related complications. A register-based cohort study.
**AUTHORS**	Trullenque-Eriksson, Anna; Tomasi, Cristiano; Eeg-Olofsson, Katarina; Berglundh, Tord; Petzold, Max; Derks, Jan

VERSION 1 – REVIEW

**REVIEWER**	Zhong, Wenjie<br>Chongqing Medical University Stomatology College
**REVIEW RETURNED**	22-Apr-2024

**GENERAL COMMENTS**	This study explores the relationship between diabetes and periodontitis based on extensive population data from Sweden over a long period, which is of significant scientific importance for understanding the interaction between these two diseases.<br><br>The research design is reasonable, and the methods for data collection and analysis are scientifically reliable, leading to solid findings. Additionally, the paper adheres to writing standards, with clear and fluent language, conforming to academic norms.<br><br>However, before meeting BMJ Open's standards, several minor concerns must be addressed:<br><br>1. It is important to note that tooth loss can also result from other factors such as caries and impacted third molars, so it is not directly equivalent to the severity of periodontitis.<br>2. In the Methods section:<br>1) Please provide the full name of "PPI".<br>2) Informed consent should be stated.<br>3. In the Discussion section:<br>There seems to be an incomplete sentence.<br>"In a study on 1114 cases and 7253 controls without diabetes, Sun et al. (2019) reported an adjusted HR of 1.7 for periodontitis in young individuals with T1D (20-40 years), based.20" However, clarification is needed regarding the "based" mentioned.<br>4. Regarding the figures:<br>1) Inconsistencies in font and unclear text color in Figure 1.<br>2) Small font size for axis titles, values, and captions in Figure 2, as well as the display issue with "≥70".<br>3) Lack of statistical significance indicators.<br>4) Font size in Figure 3 and 4 is too small.

**REVIEWER**	Yadalam, Pradeep Kumar<br>Saveetha Dental College and Hospital, Saveetha University
**REVIEW RETURNED**	03-May-2024

GENERAL COMMENTS	The article is well written, and the knowledge gap and methods were addressed well. but the results and discussion need to be explained better and need to be compared with previous studies, and in the results, all stats were not explained. Include all. Thanks

VERSION 1 – AUTHOR RESPONSE

Reviewer Reports:

Reviewer: 1
Dr. Wenjie Zhong, Chongqing Medical University Stomatology College

Comments to the Author:
This study explores the relationship between diabetes and periodontitis based on extensive population data from Sweden over a long period, which is of significant scientific importance for understanding the interaction between these two diseases.

The research design is reasonable, and the methods for data collection and analysis are scientifically reliable, leading to solid findings. Additionally, the paper adheres to writing standards, with clear and fluent language, conforming to academic norms.

However, before meeting BMJ Open's standards, several minor concerns must be addressed:

1. It is important to note that tooth loss can also result from other factors such as caries and impacted third molars, so it is not directly equivalent to the severity of periodontitis.

We acknowledge the referee's concern and have modified the text as follows:

"The occurrence of periodontitis and tooth loss (registered tooth extractions, regardless of reason for extraction) was assessed annually over the 10-year study period in the SKaPa register. A periodontitis case was defined by the presence of ≥3 teeth with probing depths of ≥6 mm, assessed by a dental professional during a routine clinical examination any time between 2010 and 2020 (Appendix p 6). Third molars were not considered for either outcome."

2. In the Methods section:
1) Please provide the full name of "PPI".

We have adjusted the text to meet both this comment and the comment by the editor:

"Patient and public involvement

There was no direct patient involvement in this study. No funds or time were allocated to PPI."

2) Informed consent should be stated.

No specific informed consent was obtained from the included individuals for this registry-based study, as approved by the ethics committee and in accordance with Swedish law (the Patient Data Act (2008:355) 7 kap).

3. In the Discussion section:
There seems to be an incomplete sentence.
"In a study on 1114 cases and 7253 controls without diabetes, Sun et al. (2019) reported an adjusted HR of 1.7 for periodontitis in young individuals with T1D (20-40 years), based.20" However, clarification is needed regarding the "based" mentioned.

Thank you for noting this typo, which has now been corrected.

4. Regarding the figures:
1) Inconsistencies in font and unclear text color in Figure 1.

Figure 1 has been modified to meet the criticism above.

2) Small font size for axis titles, values, and captions in Figure 2, as well as the display issue with "≥70".

Please see the updated version of Figure 2.

3) Lack of statistical significance indicators.

Due to the large sample size, even very small differences were statistically significant (see Appendix for full models). In the main text and figures, 95% confidence intervals are presented for all estimates. We feel that this form of presentation puts an emphasis on meaningful differences.

4) Font size in Figure 3 and 4 is too small.
Please see the updated version of Figure 3. In the revised version of our manuscript, we now suggest to remove Figure 4 as it becomes superfluous when adding Tables 2 and 3 to meet the criticism from reviewer 2 (see below).

Reviewer: 2
Dr. Pradeep Kumar Yadalam, Saveetha Dental College and Hospital, Saveetha University

Comments to the Author:
The article is well written, and the knowledge gap and methods were addressed well. but the results and discussion need to be explained better and need to be compared with previous studies, and in the results, all stats were not explained. Include all. Thanks

As requested, we have elaborated further in the results section.

As the data on diabetes-related complications are likely to be a cumbersome read, we now include two additional tables (previously in the Appendix) in the main document. Thereby, Figure 4 becomes redundant and was removed. The manuscript now provides a better/wider overview of the results. An updated version of the Appendix has also been uploaded.

The remaining results, not specifically highlighted in the main text, are presented in the Appendix.

We believe that critical studies relevant to the given context are mentioned across the introduction and discussion sections.

We have also made changes in referencing and figure/table citation order to meet the comments by the editorial office.
